# The asymmetric synthesis of an acyclic *N*-stereogenic amine

**DOI:** 10.1038/s41586-025-09905-z

**Published:** 2025-11-19

**Authors:** Chendan Zhu, Sayantani Das, Marie Sophie Sterling, Nobuya Tsuji, Spencer J. Léger, Frank Neese, Chandra Kanta De, Benjamin List

**Affiliations:** 1 Max-Planck-Institut für Kohlenforschung, Mülheim an der Ruhr, Germany; 2 Institute for Chemical Reaction Design and Discovery (ICReDD), Hokkaido University, Sapporo, Japan

**Keywords:** Asymmetric catalysis, Synthetic chemistry methodology

## Abstract

Most molecules in chemistry and biology are chiral, leading to mirror-image variants, so-called enantiomers. However, although the selective chemical synthesis of molecules in which the stereogenicity arises from a carbon atom is well-established, enantioselective approaches to nitrogen-stereogenic molecules are much less common^1–3^, and in case of acyclic, *N*-stereogenic amines, even unknown, because of their rapid pyramidal inversion. Here we describe the catalytic asymmetric synthesis of stable, acyclic *N*-stereogenic amines by the addition of enol silanes to nitronium ions that ion pair to a confined chiral anion. In the produced so-called anomeric amines, the commonly observed isomerization is slowed down by two *N*-oxy-substituents, which hamper nitrogen inversion. The important stereogenicity-creating step challenges previously established stereochemical descriptors of enantiodifferentiation. Computational studies provide further insight into the origin of the observed stereocontrol. Our work opens up a new avenue to investigate the fascinating and previously underexplored chemistry of enantiopure anomeric amines.

## Abstract (web summary)

Catalytic asymmetric synthesis of stable, acyclic *N*-stereogenic amines by the addition of enol silanes to nitronium ions that ion pair to a confined chiral anion is described.

## Main

Despite the wealth of studies concerning enantiopure tetrahedral, *C*-stereogenic molecules and related structures, chiral pyramidal molecules have been less investigated. Their routine chemical synthesis can be realized only in the case of *P*-stereogenic phosphines^1^ and *S*-stereogenic sulfoxides^2^ and sulfonium salts^3^. Recently, one of the first asymmetric syntheses of bridged, helically chiral *O*-stereogenic oxonium ions^4,5^ has been described. By contrast, *N*-stereogenic, tertiary amines are generally considered configurationally unstable^6^ under standard laboratory conditions^7^ and bridging^8,9^ or quaternarization to the corresponding tetrahedral ammonium ions is usually required to avoid racemization by the so-called umbrella inversion^10–14^. During recent decades, marked progress has been made in synthesizing *N*-stereogenic cyclic amines, with or without additional stereogenic elements^15–25^.

**Fig. 1:**
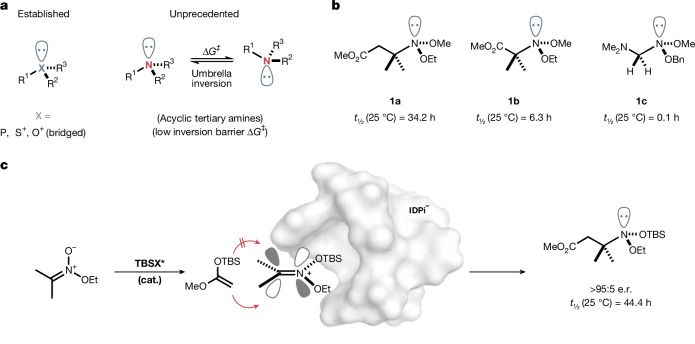
Asymmetric synthesis of pyramidal molecules. **a**, Approaches to *P*-stereogenic phosphines, *S*-stereogenic sulfoxides and bridged *O*-stereogenic oxonium ions have been established. By contrast, acyclic *N*-stereogenic amines have not been synthesized previously. **b**, Examples of configurationally stabilized acyclic, tertiary amines. **c**, Catalytic asymmetric addition of a silyl ketene acetal to an in situ-generated silylnitronium ion (this work).

To the best of our knowledge, however, the asymmetric synthesis of an acyclic *N*-stereogenic amine has not previously been accomplished and is reported here with the catalytic asymmetric addition of enol silanes to nitronium ions (Fig. 1c). Already in 1890, Hantzsch and Werner predicted that chiral trivalent amines may exist in optically active form^26^. However, attempts to obtain these enantiomers were unsuccessful. Later, Meisenheimer concluded that chiral amines cannot be resolved because of their rapid pyramidal inversion^11^. Our investigation was inspired by the realization that *N*-substituents have a marked effect on the inversion barrier of acyclic tertiary amines. It has been predicted that electronegative and Lewis basic *N*-substituents, such as oxygen, strongly enhance the pyramidal inversion barrier^6^. This effect is particularly pronounced in bis-oxy-substituted^27,28^, so-called anomeric amines, as we could confirm with initial computations^29,30^ (Supplementary Information). In 1980, a previous study successfully isolated anomeric amines **1a** and **1b** by resolution and determined their inversion energy barrier and half-life^27^.

We have recently described an imidodiphosphorimidate (IDPi)-catalysed addition of silyl ketene acetals (SKA) to in situ-generated *bis*-silyl nitronium ions, furnishing enantiopure β^3^-amino acid derivatives^31^. The question arose whether it would be possible to add SKAs to silyl nitronium ions that are formally derived from symmetric carbonyl compounds, such that no *C*-stereogenic centre would be created. With two different oxygen substituents on nitrogen, the enantioselective creation of configurationally stable, only *N*-stereogenic amines may be realized. We describe here a catalytic asymmetric approach to enantiopure acyclic *N*-stereogenic amines (Fig. 1c).

## Reaction development

**Fig. 2:**
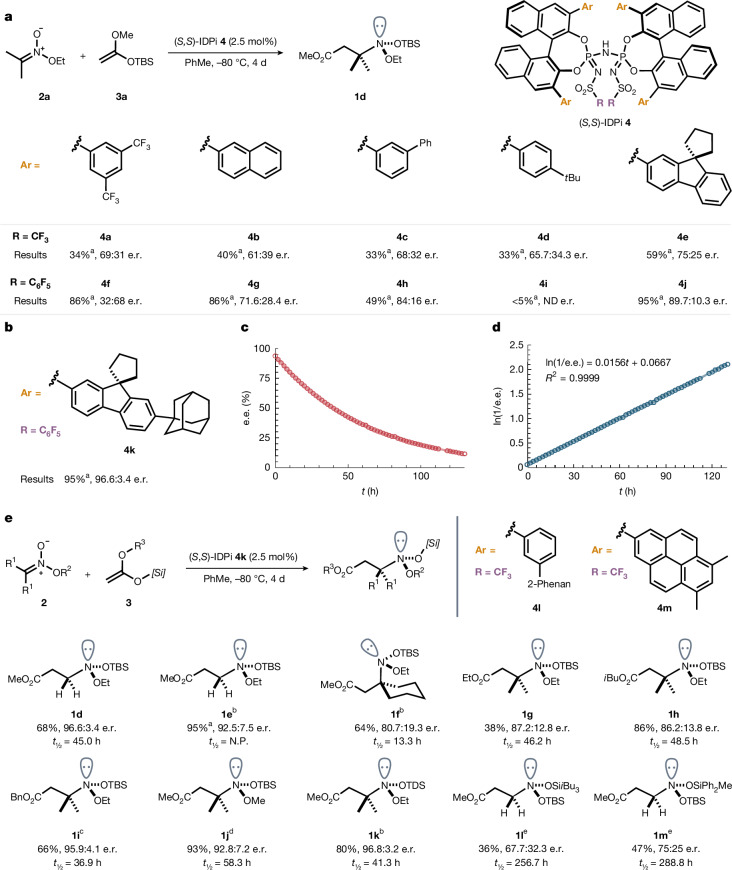
Reaction development, configurational stability study and substituent effect on nitrogen. **a**, ^a^Optimization of the catalytic reaction was carried out as mentioned above, and the yield was determined by ^1^H NMR using an internal standard. **b**, Under the optimized condition result for product **1c**. **c**,**d**, Configurational stability study of product **1d** using HPLC method at 25 °C, and the obtained data were plotted as e.e. compared with time. **e**, Evaluation of substituent effect around the nitrogen centre of the anomeric amines. ^b^Using catalyst **4h**. ^c^Using catalyst **4l**. ^d^Using catalyst **4m**. ^e^Using catalyst **4d**. 2-Phenan, 2-Phenanthryl; TBS, *tert*-butyldimethylsilyl; TDS, thexyldimethylsilyl; Bn, benzyl; PhMe, toluene; ND, not determined; NP, not possible.

We started our study by synthesizing alkyl nitronate **2a** (ref. ^32^), which can be prepared from the sodium salt of 2-nitropropane and the corresponding Meerwein salt at low temperature (Supplementary Information). At the onset of our studies, we examined a series of highly acidic and confined IDPi Brønsted acid catalysts^33^, varying the aryl substituents (Ar) at the 3,3′-positions and the sulfur substituents (R) at the inner core, in the reaction of isopropyl nitronate **2a** with *tert*-butyldimethylsilyl (TBS) ketene acetal **3a** to give amine **1d** (Fig. 2a). Chiral Brønsted acids **4a**–**4e**, with the strongly electron-withdrawing triflyl (Tf) group were active and furnished the desired product with promising enantioselectivity. When the Tf group was replaced with a pentafluoro sulfonyl (C_6_F_5_SO_2_) group, catalysts **4f**–**4j** gave even higher enantioselectivity. Among them, spiro-fluorenyl substituted catalyst **4j** was found to be the best and delivered the product in 95% yield with 90:10 enantiomeric ratio (e.r.). Thus, catalyst **4j** was considered for further modifications. A systematic investigation of different alkyl groups at the 7-position of the spiro-fluorenyl wing was conducted (Supplementary Information), revealing catalyst **4k** as optimal, furnishing product **1d** in 68% yield with excellent enantioselectivity (96.6:3.4 e.r.) (Fig. 2b). The configurational stability of amine **1d** was investigated by monitoring^34^ the e.r. over time at 25 °C (Fig. 2c,d). The half-life (*t*_1/2_) of amine **1d** was determined to be 44.4 h, corresponding to an activation free energy (Δ*G*^‡^ at 25 °C) of 25.2 kcal mol^−1^. Amine **1d** exhibited a higher half-life than the previously reported anomeric amine **1a**. Although the precise cause of this enhanced stability remains unclear, we speculate that either the presence of the silicon atom or the increased steric bulk of that substituent compared with a methoxy group may be responsible. Our observation prompted us to further explore the influence of different nitrogen substituents. In this context, we varied *N*-substituents in alkyl nitronates **2** and also investigated different ketene acetals **3**, as summarized in Fig. 2e. Initially, a less sterically demanding nitronate derived from nitromethane was examined. Its reaction yielded product **1e** in 95% yield (¹H NMR) with a good e.r. using catalyst **4 h**. However, amine **1e** exhibited very low chemical stability at 25 °C, and its half-life could not be reliably determined. We then turned our attention to a cyclic nitronate derived from nitrocyclohexane. Under optimized conditions, product **1f** was obtained in good yield with moderate enantioselectivity. This compound showed more than three-fold lower half-life (13.3 h) compared with the model substrate **1d**. This unexpected result further motivated us to systematically modify the structure at the nitrogen centre to better understand its influence on configurational stability (Fig. 2e). For example, we varied the ketene acetal component, which affects both the ester and the silyl substituent in the final product. Altering the alkyl group in the SKA from ethyl to isobutyl to benzyl (compounds **1g**–**1i**) delivered the desired products in moderate to good e.r.s and with comparable half-lives. Replacing the nitrogen-bound ethoxy group with a methoxy group furnished product **1j** in good yield and e.r., along with a notably increased half-life. Introduction of the bulky thexyldimethylsilyl (TDS) group afforded product **1k** in excellent yield and enantioselectivity when using catalyst **4h**. Finally, replacing the R^2^-alkyl substituent of the nitronates with another silyl group yielded products **1l** and **1m**, which were obtained in moderate yields and enantioselectivities, but showed the highest half-lives observed in this study.

**Fig. 3:**
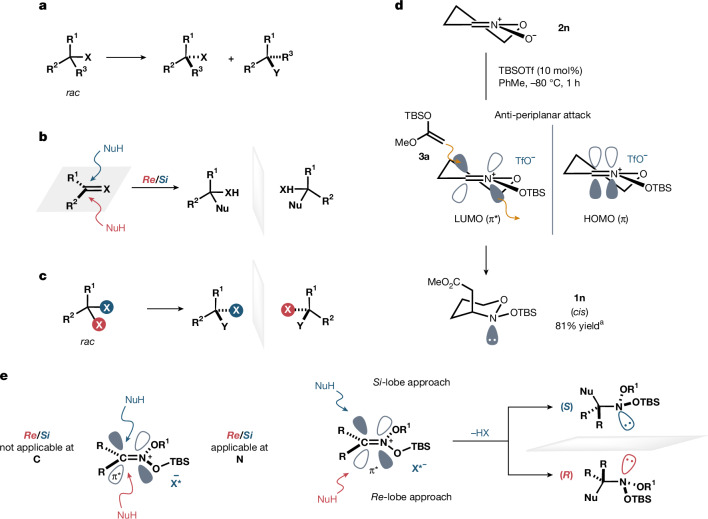
Stereochemical considerations require previously established stereochemical descriptors of enantiodifferentiation. **a**, Enantiomer differentiation. **b**, Enantiofacial differentiation. **c**, Enantiogroup differentiation. **d**, Experimental result confirms the anti-periplanar mechanism of the reaction. **e**, This work: enantiolobal differentiation. ^a^NMR yield.

With the establishment of an asymmetric synthesis of an acyclic nitrogen-stereogenic amine, a particularly interesting question was how the enantiodifferentiation could be best described. The formation of a silyl nitronium cation and IDPi counteranion pair during the reaction of an SKA with a nitronate has been previously established in our amino acid ester synthesis^31^. In that reaction, the enantiotopic planes that are spanned by the reacting carbon atom and the groups attached to it are distinguishable as *Re*- and *Si*-faces. However, as electrophile **2a** features identical substituents at the carbon atom of the nitronium ion, this formal distinction cannot be made here, although the corresponding *Re* and *Si* descriptors are fully functional with regard to the corresponding nitrogen atom. A different type of enantiodifferentiation must, therefore, be used to describe our transformation. The previously considered comprehensive nomenclature^35^ distinguishes three modes of enantiotopos differentiation, enantiomer differentiation (Fig. 3a), enantiofacial differentiation (Fig. 3b) and enantio(topic)-group differentiation (Fig. 3c), all of which, however, are not applicable to the current situation. Previous studies^36,37^ demonstrated that the addition of SKA **3a** to substituted cyclic nitronates occurs in an anti-periplanar fashion such that the *N*-lone pair builds up anti to the newly created σ-bond. Inspired by this observation, we synthesized the unsubstituted cyclic nitronate **2n** and subjected it to a reaction with SKA **3a** and TBSOTf as a catalyst. As expected, this experiment resulted in the exclusive formation of *cis*-**1n**, consistent with an anti-periplanar addition pathway (Fig. 3d). An anti-periplanar mode of addition should also operate in the corresponding reactions of acyclic nitronates **2**. In the key bond-forming step, electron density is transferred from the silyl ketene acetal nucleophile to the nitronium ion electrophile. The electronic movement formally occurs from the π-HOMO (highest occupied molecular orbital) of the nucleophile into the π*-LUMO (lowest unoccupied molecular orbital) of the electrophile. This particular antibonding orbital features two nodes, and identical phases of the symmetric lobes are found on the opposite planes of the C=N–π system. The transfer of electron density from the nucleophile into one π*-orbital lobe at carbon initiates not only the formation of the new C–C–σ-bond but also the breaking of the C=N–π bond and the concomitant buildup of electron density at the nitrogen atom, ultimately shaping the critical lone pair on the opposite side of the π-plane. Accordingly, distinguishing the two π* lobes at carbon determines the absolute configuration at nitrogen and thus, this unique type of enantioselectivity may be described as enantiolobal differentiation (Fig. 3e).

**Fig. 4:**
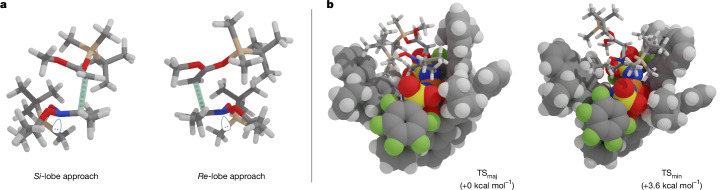
Comparison of transition states leading to the major and minor isomers. **a**, Visualization of the substrates in the transition states with the forming lone pairs indicated. Chiral counteranions are omitted for clarity. **b**, Visualization of the complexes in the transition states. Substrates are shown as sticks, and chiral counteranions are shown as spheres. The free energies were obtained at SMD(toluene)-*ω*B97M-V/def2-TZVPP//PBE-D4/def2-SVP level of theory.

With this hypothesis in mind, we calculated the transition states leading to the major enantiomer (**TS**_maj_) and the minor enantiomer (**TS**_min_) to gain insights into the key enantiolobal differentiation in our system with IDPi catalyst **4j**. The energy difference between **TS**_maj_ and **TS**_min_ qualitatively aligns with the experimental results. For both enantiomers, as expected, the addition of the SKA to the nitronium ion builds electron density at the nitrogen atom on the opposite side of the π-plane, eventually leading to the formation of the corresponding nitrogen-centred chirality (Fig. 4a). The origin of enantioselectivity appears to arise from a finely tuned microenvironment that induces steric hindrance between the bulky silyl group and the chiral counteranion (Fig. 4b). In both cases, the nucleophile approaches in a manner that minimizes steric hindrance between the bulky silyl group and the wing of the chiral counterion. By contrast, the *Si*-lobe approach gains marked stabilization through non-covalent interactions between the substrates and Lewis basic functionalities in the catalytically active site, whereas the *Re*-lobe approach proceeds with fewer such interactions, presumably because of the silyl group on the nucleophile orienting outwards (Supplementary Fig. 2). This is further supported by a distortion-interaction analysis^38^ (Supplementary Table 12).

In conclusion, we have developed a strategy for the catalytic asymmetric synthesis of configurationally stable acyclic amines with excellent enantioselectivity. Central to this achievement is the use of confined, enzyme-like IDPi catalysts, which provide a uniquely defined chiral environment capable of controlling the stereochemistry at a pyramidal nitrogen centre, long considered one of the most challenging stereochemical elements in chemical synthesis. This work establishes a new paradigm for stereocontrol at nitrogen. We anticipate that the conceptual foundation presented here will inspire further investigations, ultimately advancing the broader field of asymmetric catalysis and molecular stereochemistry.

## Online content

Any methods, additional references, Nature Portfolio reporting summaries, source data, extended data, supplementary information, acknowledgements, peer review information; details of author contributions and competing interests; and statements of data and code availability are available at 10.1038/s41586-025-09905-z.

## Supplementary information

Supplementary Information This file contains Supplementary Methods, Supplementary References, NMR spectra and HPLC chromatograms.

## Data Availability

The experimental procedures and analytical data supporting the findings of the study are available in the paper and the Supplementary Information.
