# Supplementary material for: The asymmetric synthesis of an acyclic N-stereogenic amine
Source: Nature. 2025 Nov 19;649(8097):621–5. doi: 10.1038/s41586-025-09905-z (PMC12804080; doi:10.1038/s41586-025-09905-z)
Supplement: Supplementary file 1 — This file contains Supplementary Methods, Supplementary References, NMR spectra and HPLC chromatograms. [file 41586_2025_9905_MOESM1_ESM.pdf]

nature portfolio

https://doi.org/10.1038/s41586-025-09905-z

Supplementary information

The asymmetric synthesis of an acyclic *N*-stereogenic amine

In the format provided by the authors and unedited

Supplementary Material

The Asymmetric Synthesis of an Acyclic *N*-Stereogenic Amine

Chendan Zhu[1#], Sayantani Das[1#], Marie Sophie Sterling[1], Nobuya Tsuji[2], Spencer J. Léger[1], Frank Neese[1], Chandra Kanta De[1*] & Benjamin List[1*]

[1]Max-Planck-Institut für Kohlenforschung, Kaiser-Wilhelm-Platz 1, 45470 Mülheim an der Ruhr, Germany.

[2]Institute for Chemical Reaction Design and Discovery (ICReDD), Hokkaido University, Sapporo 001- 0021, Japan;

*e-mail: de@kofo.mpg.de (C.K.D); list@kofo.mpg.de (B.L.); [#]Contributed equally.

Contents

1	Supplementary Methods	2
	General Information	2
	Synthesis of Catalysts	3
	Nitronate Synthesis	8
	Reaction Development with Different Catalysts	13
	General Procedure for the Addition of Silyl Ketene Acetals to Ethyl Nitronates (GP2)	14
	Configurational Stability Study	19
	Determination of Absolute Configuration	40
	DFT Coordinates Data	43
2	Supplementary References	70
3	NMR Spectra	73
4	HPLC Chromatograms	98

1 Supplementary Methods

General Information

Unless otherwise stated, all reagents were purchased from commercial suppliers and used without further purification. All solvents used in the reactions were distilled from appropriate drying agents prior to use. Reactions were monitored by thin layer chromatography (TLC) on silica gel pre-coated plastic sheets (0.2 mm, Macherey-Nagel). Visualization was accomplished by irradiation with UV light at 254 nm and/or PMA stain. Column chromatography was performed on Merck silica gel (60, particle size 0.040–0.063 mm). ^{1}H and ^{13}C NMR spectra were recorded on a Bruker AV-500 spectrometer in deuterated solvents. Proton chemical shifts are reported in ppm (δ) relative to the solvent resonance employed as the internal standard ($CDCl_3$, CD_2Cl_2 and C_6D_6 δ 7.26, 5.32 and 7.16 ppm, respectively). Data are reported as follows: chemical shift, multiplicity (s = singlet, d = doublet, t = triplet, q = quartet, p = pentet, s = sextet, h = heptet, m = multiplet, br = broad), coupling constants (Hz) and integration. ^{13}C chemical shifts are reported in ppm with the solvent resonance as the internal standard ($CDCl_3$, CD_2Cl_2 and C_6D_6 δ 77.16, 54.00 and 128.00 ppm respectively). High resolution mass spectra were determined on a Bruker APEX III FTMS (7 T magnet). Optical rotations were determined with an Autopol IV polarimeter (Rudolph Research Analytical) at 589 nm and 25 °C. Data are reported as follows: $[\alpha]_{\lambda}^{temp}$, concentration (*c* in g/100 mL), and solvent. CD spectra were measured on J-1100 CD spectrometer (JASCO). Enantiomeric ratios (er) were determined by HPLC analysis employing a chiral stationary phase column specified in the individual experiment, by comparing the samples with the appropriate racemic mixtures.

Synthesis of Catalysts

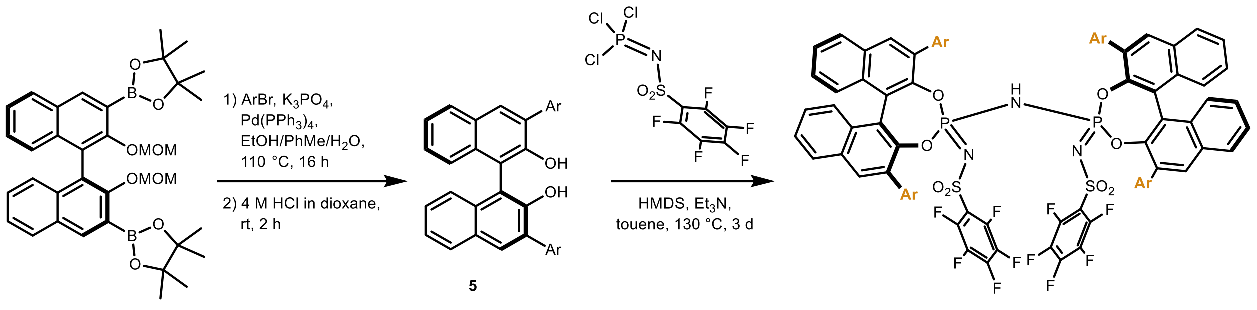

2'-Bromo-7'-adamantylspiro[cyclopentane-1,9'-fluorene] (6k): The synthetic procedure for the preparation of the

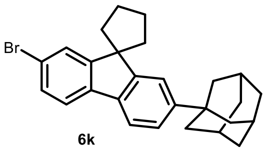

6k

desired bromo-compound was adopted from the procedure described in literature.[1] **^{1}H NMR** (500 MHz, $CDCl_3$) δ 7.60 (d, *J* = 8.0 Hz, 1H), 7.57–7.48 (m, 2H), 7.46–7.39 (m, 2H), 7.35 (dd, *J* = 8.0, 1.9 Hz, 1H), 2.22–2.02 (m, 11H), 2.02–1.94 (m, 6H), 1.87–1.76 (m, 6H).

^{13}C NMR (126 MHz, $CDCl_3$) δ 156.8, 153.8, 151.8, 138.7, 136.1, 129.8, 126.3, 123.9, 120.8, 120.7, 119.3, 119.3, 58.0, 43.6, 39.9, 37.0, 36.7, 29.2, 27.1.

HRMS *m/z* (EI): calcd. for $C_{27}H_{29}Br_1$ $[M]^+$: 432.1447; found: 432.1455.

(*S*)-3,3'-bis(2'-adamantylspiro[cyclopentane-1,9'-fluoren]-7'-yl)-[1,1'-binaphthalene]-2,2'-diol--methane (1/1) (5k): The synthetic procedure for the preparation of the desired Binol was adopted from the procedure described in literature.[1] **^{1}H NMR** (500 MHz, $CDCl_3$) δ 8.01 (s, 2H), 7.87 (d, *J* = 8.0 Hz, 2H), 7.70 (d, *J* = 8.4 Hz, 4H), 7.60 (d, *J* = 7.9 Hz, 4H), 7.39–7.16 (m, 10H), 5.40 (s, 2H), 2.18–2.00 (m, 22H), 1.92 (d, *J* = 3.2 Hz, 12H), 1.85–1.63 (m, 12H).

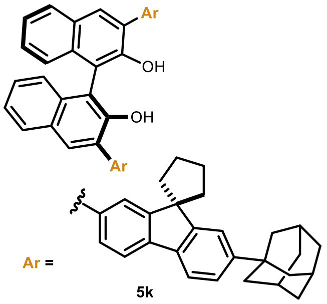

5k

^{13}C NMR (126 MHz, $CDCl_3$) δ 155.0, 154.6, 151.5, 150.3, 139.5, 136.8, 136.0, 133.1, 131.3, 131.3, 129.7, 128.5, 128.3, 127.3, 124.6, 124.4, 124.3, 123.7, 119.5, 119.4, 119.3, 112.9, 58.0, 43.6, 40.1, 40.1, 37.0, 36.7, 29.2, 27.2.

HRMS *m/z* (ESI): calcd. for $C_{74}H_{69}O_2$ $[M–H]^–$: 989.5303; found: 989.5316.

(*S*,*S*)- N,N'-(azanediylbis(2,6-bis(spiro[cyclopentane-1,9'-fluoren]-7'-yl)-4l5-dinaphtho[2,1-d:1',2'-f][1,3,2]dioxaphosphepine-4-yl-4-ylidene))bis(2,3,4,5,6-pentafluorobenzenesulfonamide) (4j):

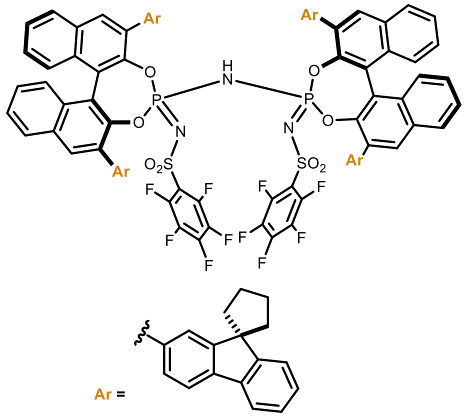

4j

In a flame dried Schlenk flask under Ar, Phosphorimidoyl trichloride (0.24 g, 0.63 mmol, 2.0 equiv.) and (*S*)-3,3'-di(spiro[cyclopentane-1,9'-fluoren]-2'-yl)-[1,1'-binaphthalene]-2,2'-diol-methane (1/1)[1] (0.46 g, 0.63 mmol, 2.0 equiv.) was added. The solids were dissolved in toluene (5 mL) and then triethylamine (0.51 g, 0.71 mL, 5.06 mmol, 16 equiv.) was added at room temperature. The mixture was stirred at room temperature for 60 min and then hexamethyldisilazane (0.05 g, 0.066 mL, 0.32 mmol, 1.0 equiv.) was added. After being stirred at room temperature for additional 10 min, the mixture was heated to 130 °C for 3 d. The reaction mixture was cooled to room temperature, diluted with DCM and HCl (10% aq.). Two phases were separated and the aqueous layer was washed with DCM (2x50 mL). The combined organic layer was dried over Na_2SO_4, filtered and concentrated *in vacuum*. Purification by silica gel column chromatography (10% EtOAc in pentane *v/v*) followed by the acidification with Dowex in DCM and drying *in vacuum* afforded the desired compound as an off-white solid (0.48 g, 75%).

^{1}H NMR (501 MHz, CD_2Cl_2) δ 8.11 (s, 2H), 8.05 (d, *J* = 7.7 Hz, 2H), 8.01 (d, *J* = 8.0 Hz, 2H), 7.75 (ddd, *J* = 8.0, 6.4, 1.6 Hz, 2H), 7.62–7.56 (m, 4H), 7.55–7.47 (m, 6H), 7.44 (dd, *J* = 6.9, 1.4 Hz, 2H), 7.39 (dd, *J* = 6.9, 1.3 Hz, 2H), 7.37–7.31 (m, 8H), 7.29–7.16 (m, 12H), 7.05–6.94 (m, 4H), 6.65 (dd, *J* = 7.9, 1.6 Hz, 2H), 2.37–2.29 (m, 2H), 2.17–1.91 (m, 22H), 1.88–1.74 (m, 8H).

^{13}C NMR (126 MHz, CD_2Cl_2) δ 155.6, 155.2, 155.0, 154.6, 144.5, 143.3, 139.7, 139.4, 139.3, 139.3, 135.3, 134.7, 134.2, 132.4, 132.3, 132.3, 132.0, 131.9, 129.6, 129.2, 128.9, 128.7, 128.1, 127.9, 127.7, 127.3, 127.3, 127.2, 127.1, 124.4, 124.2, 123.6, 123.3, 123.2, 122.9, 120.2, 120.1, 119.7, 118.8, 58.3, 58.1, 40.8, 40.6, 39.9, 39.5, 27.7, 27.5, 27.4, 27.4. (other signals not detected or observed)

^{19}F NMR (471 MHz, CD_2Cl_2) δ –136.20 (d, *J* = 22.3 Hz), –146.58 (t, *J* = 22.3 Hz), –160.08 (t, *J* = 19.6 Hz).

^{31}P NMR (203 MHz, CD_2Cl_2) δ –14.56.

HRMS *m/z* (ESI): calcd. for $C_{120}H_{80}F_{10}N_3O_8P_2S_2$ $[M–H]^–$: 2006.4708; found: 2006.4728.

(*S*,*S*)- N,N'-(azanediylbis(2,6-bis(2'-adamantylspiro[cyclopentane-1,9'-fluoren]-7'-yl)-4l5-dinaphtho[2,1-d:1',2'-f][1,3,2]dioxaphosphepine-4-yl-4-ylidene))bis(2,3,4,5,6-pentafluorobenzenesulfonamide) (4k): In a flame dried Schlenk flask under Ar, Phosphorimidoyl trichloride (0.031 g, 0.08 mmol, 2.0 equiv.) and (*S*)-3,3'-bis(2'-adamantylspiro[cyclopentane-1,9'-fluoren]-7'-yl)-[1,1'-binaphthalene]-2,2'-diol-methane (1/1) (0.08 g, 0.08mmol, 2.0 equiv.) was added. The solids were dissolved in toluene (1 mL) and then triethylamine (0.07 g, 0.09 mL, 0.65 mmol, 16 equiv.) was added at room temperature. The mixture was stirred at room temperature for 60 min and then hexamethyldisilazane (0.065 g, 0.084 mL, 0.04 mmol, 1.0 equiv.) was added. After being stirred at room temperature for additional 10 min, the mixture was heated to 130 °C for 3 d. The reaction mixture was cooled to room temperature, diluted with DCM and HCl (10% aq.). Two phases were separated and the aqueous layer was washed with DCM (2x50 mL). The combined organic layer was dried over Na_2SO_4, filtered and concentrated *in vacuum*. Purification by silica gel column chromatography (10% EtOAc in pentane *v/v*) followed by the acidification with Dowex in DCM and drying *in vacuum* afforded the desired compound as an off-white solid (0.071 g, 69%).

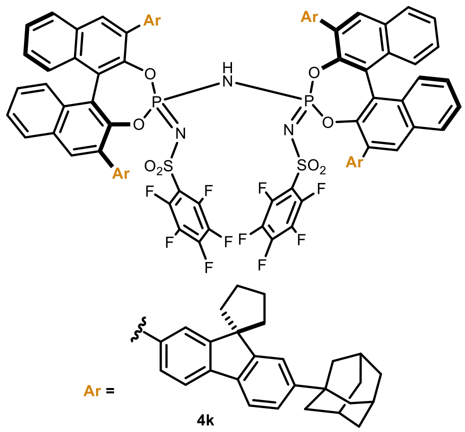

4k

^{1}H NMR δ 8.10 (s, 2H), 8.04 (d, *J* = 8.2 Hz, 2H), 7.98 (d, *J* = 8.2 Hz, 2H), 7.75 (td, *J* = 7.3, 3.9 Hz, 2H), 7.57 (t, *J* = 7.6 Hz, 2H), 7.55–7.52 (m, 2H), 7.52–7.43 (m, 4H), 7.42–7.37 (m, 4H), 7.37–7.28 (m, 8H), 7.29–7.23 (m, 4H), 7.20 (d, *J* = 8.7 Hz, 2H), 7.16 (dd, *J* = 8.0, 1.9 Hz, 2H), 7.06 (d, *J* = 8.0 Hz, 2H), 7.02 (d, *J* = 7.9 Hz, 2H), 6.94 (d, *J* = 9.0 Hz, 2H), 6.58 (dd, *J* = 7.9, 1.7 Hz, 2H), 2.16–1.93 (m, 46H), 1.88–1.69 (m, 46H).

^{13}C NMR (126 MHz, CD_2Cl_2) δ 155.5, 155.3, 155.0, 154.9, 152.0, 151.8, 144.5, 143.6, 139.9, 139.4, 136.8, 136.7, 134.8, 134.7, 134.3, 132.4, 132.3, 132.3, 132.0, 131.9, 129.6, 129.2, 128.9, 128.7, 127.6, 127.3, 127.3, 127.2, 127.0, 124.4, 124.0, 123.9, 123.5, 123.0, 119.8, 119.6, 119.4, 118.6, 58.4, 58.1, 44.0, 43.9, 41.0, 40.7, 40.0, 39.5, 37.4, 37.3, 37.0, 37.0, 34.7, 30.3, 29.8, 29.7, 27.8, 27.6, 27.4, 22.9, 14.4. (other signals not detected or observed)

^{19}F NMR (471 MHz, CD_2Cl_2) δ –136.05 (d, *J* = 21.2 Hz, 4F), –146.37 (t, *J* = 22.3 Hz, 2F), –160.26 (t, *J* = 20.1 Hz, 4F).

^{31}P NMR (203 MHz, CD_2Cl_2) δ –14.90.

HRMS *m/z* (ESI): calcd. for $C_{160}H_{136}F_{10}N_3O_8P_2S_2$ $[M–H]^–$: 2542.9090; found: 2542.9107.

(*S*,*S*)- N,N'-(azanediylbis(2,6-bis(2'-methylspiro[cyclopentane-1,9'-fluoren]-7'-yl)-4l5-dinaphtho[2,1-d:1',2'-f][1,3,2]dioxaphosphepine-4-yl-4-ylidene))bis(2,3,4,5,6-pentafluorobenzenesulfonamide) (4n): In a flame dried

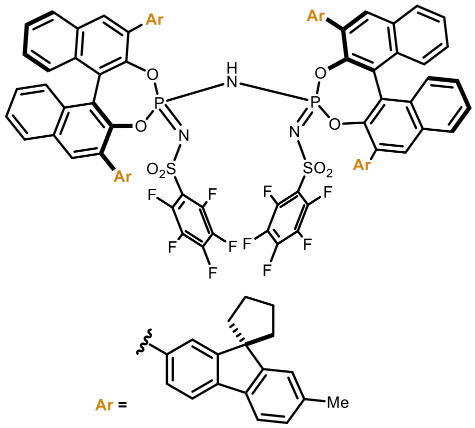

4n

Schlenk flask under Ar, Phosphorimidoyl trichloride (0.046 g, 0.12 mmol, 2.0 equiv.) and (*S*)-3,3'-bis(2'-methylspiro[cyclopentane-1,9'-fluoren]-7'-yl)-[1,1'-binaphthalene]-2,2'-diol-methane (1/1)[1] (0.09 g, 0.12 mmol, 2.0 equiv.) was added. The solids were dissolved in toluene (1 mL) and then triethylamine (0.1 g, 0.14 mL, 0.96 mmol, 16 equiv.) was added at room temperature. The mixture was stirred at room temperature for 60 min and then hexamethyldisilazane (0.01 g, 0.013 mL, 0.06 mmol, 1.0 equiv.) was added. After being stirred at room temperature for additional 10 min, the mixture was heated to 130 °C for 3 d. The reaction mixture was cooled to room temperature, diluted with DCM and HCl (10% aq.). Two phases were separated and the aqueous layer was washed with DCM (2x50 mL). The combined organic layer was dried over Na_2SO_4, filtered and concentrated *in vacuum*. Purification by silica gel column chromatography (10% EtOAc in pentane *v*/*v*) followed by the acidification with Dowex in DCM and drying *in vacuum* afforded the desired compound as an off-white solid (0.085 g, 69%).

^{1}H NMR (501 MHz, $CDCl_3$) δ 8.04 (s, 2H), 7.96 (dd, *J* = 20.2, 8.4 Hz, 4H), 7.70–7.61 (m, 2H), 7.56–7.46 (m, 4H), 7.45–7.36 (m, 6H), 7.33–7.23 (m, 8H), 7.23–7.09 (m, 10H), 7.05 (d, *J* = 7.9 Hz, 4H), 6.96 (d, *J* = 7.7 Hz, 2H), 6.83 (d, *J* = 7.9 Hz, 2H), 6.54 (dd, *J* = 7.9, 1.7 Hz, 2H), 2.39 (s, 6H), 2.33 (s, 6H), 2.32–2.23 (m, 2H), 2.14–1.75 (m, 30H).

^{19}F NMR (471 MHz, CD_2Cl_2) δ –136.19 (d, *J* = 22.3 Hz, 4F), –146.72 (t, *J* = 20.7 Hz, 2F), –160.18 (t, *J* = 19.6 Hz, 4F).

^{31}P NMR (203 MHz, $CDCl_3$) δ –14.30.

HRMS *m/z* (ESI): calcd. for $C_{124}H_{88}F_{10}N_3O_8P_2S_2$ $[M–H]^-$: 2062.5334; found: 2062.5346.

(*S*,*S*)- N,N'-(azanediylbis(2,6-bis(2'-*tert*butylspiro[cyclopentane-1,9'-fluoren]-7'-yl)-4l5-dinaphtho[2,1-d:1',2'-f][1,3,2]dioxaphosphepine-4-yl-4-ylidene))bis(2,3,4,5,6-pentafluorobenzenesulfonamide) (4m-4-yl-4-ylidene))bis(2,3,4,5,6-pentafluorobenzenesulfonamide) (4o): In a flame dried Schlenk flask under Ar, Phosphorimidoyl trichloride (0.046 g, 0.12 mmol, 2.0 equiv.) and (*S*)-3,3'-bis(2'-*tert*butylspiro[cyclopentane-1,9'-fluoren]-7'-yl)-[1,1'-binaphthalene]-2,2'-diol-methane (1/1)[1] (0.1 g, 0.12 mmol, 2.0 equiv.) was added. The solids were dissolved in toluene (1 mL) and then triethylamine (0.1 g, 0.14 mL, 0.96 mmol, 16 equiv.) was added at room temperature. The mixture was stirred at room temperature for 60 min and then hexamethyldisilazane (0.01 g, 0.013 mL, 0.06 mmol, 1.0 equiv.) was added. After being stirred at room temperature for additional 10 min, the mixture was heated to 130 °C for 3 d. The reaction mixture was cooled to room temperature, diluted with DCM and HCl (10% aq.). Two phases were separated and the aqueous layer was washed with DCM (2x50 mL). The combined organic layer was dried over Na_2SO_4, filtered and concentrated *in vacuum*. Purification by silica gel column chromatography (5% EtOAc in pentane *v*/*v*) followed by the acidification with Dowex in DCM and drying *in vacuum* afforded the desired compound as an off-white solid (0.095 g, 71%).

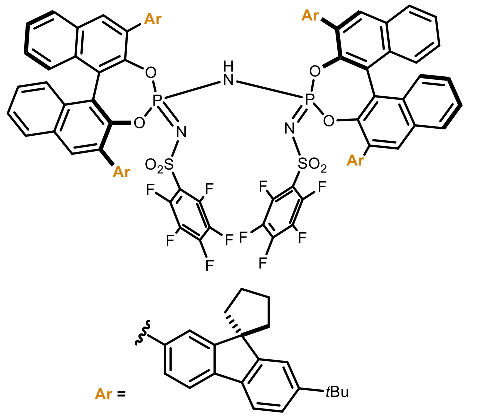

4o

^{1}H NMR (501 MHz, CD_2Cl_2) δ 8.10 (s, 2H), 8.04 (d, *J* = 7.4 Hz, 2H), 7.98 (d, *J* = 8.4 Hz, 2H), 7.75 (ddd, *J* = 8.2, 5.7, 2.3 Hz, 2H), 7.58 (ddd, *J* = 8.2, 6.7, 1.2 Hz, 2H), 7.53 (d, *J* = 1.7 Hz, 2H), 7.51 (dd, *J* = 5.8, 1.3 Hz, 4H), 7.42–7.28 (m, 14H), 7.22 (d, *J* = 1.7 Hz, 2H), 7.21 (d, *J* = 1.6 Hz, 4H), 7.09 (dd, *J* = 12.7, 8.0 Hz, 4H), 6.92 (dd, *J* = 8.0, 1.7 Hz, 2H), 6.59 (dd, *J* = 8.0, 1.7 Hz, 2H), 2.34–2.24 (m, 2H), 2.12–2.02 (m, 12H), 2.01–1.92 (m, 8H), 1.89–1.70 (m, 10H), 1.34 (s, 18H), 1.28 (s, 18H).

^{13}C NMR (126 MHz, CD_2Cl_2) δ 155.4, 155.2, 154.9, 154.8, 151.6, 151.4, 143.5, 139.8, 139.3, 136.7, 136.6, 134.9, 134.7, 134.3, 132.4, 132.3, 132.2, 132.0, 131.8, 129.6, 129.2, 128.9, 128.7, 127.6, 127.3, 127.3, 127.1, 127.1, 127.0, 124.3, 124.3, 124.0, 123.6, 122.9, 120.1, 119.9, 119.6, 119.5, 119.5, 118.6, 58.4, 58.1, 41.0, 40.7, 39.9, 39.5, 35.4, 35.4, 31.9, 27.8, 27.5, 27.3. (other signals not detected or observed)

^{19}F NMR (471 MHz, CD_2Cl_2) δ –136.26 (d, *J* = 22.3 Hz, 4F), –146.34 (t, *J* = 23.8 Hz, 2F), –160.11 (t, *J* = 20.1 Hz, 4F).

^{31}P NMR (203 MHz, CD_2Cl_2) δ –15.28.

HRMS *m/z* (ESI): calcd. for $C_{136}H_{112}F_{10}N_3O_8P_2S_2$ $[M–H]^-$: 2230.7212; found: 2230.7216.

Catalysts **4a**,[2] **4b**,[3] **4c**,[4] **4d**,[5] **4e**,[2] **4f**,[6] **4g**,[6] **4h**,[4] **4i**,[7] **4l**,[8] **4m**[3] were prepared following literature-known methods.

Ethyl propan-2-ylideneazinate (2d):

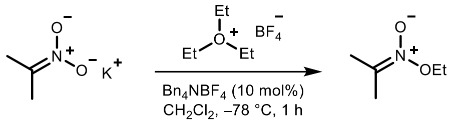

To a flame dried Schlenk flask under Ar and equipped with a magnetic stirring bar, potassium propan-2-ylideneazinate (763 mg, 6 mmol, 1.2 equiv.) and anhydrous dichloromethane (6 mL) were added. The suspension was cooled to –78 °C and the triethyloxonium tetrafluoroborate solution (2 M in anhydrous dichloromethane, 2.5 mL, 5 mmol 1.0 equiv.) was added dropwise followed by tetrabenzylammonium tetrafluoroborate solution (0.8 M in anhydrous dichloromethane, 0.625 mL, 0.5 mmol, 0.1 equiv.). The reaction mixture was stirred at –78 °C for 1 hour. The crude NMR in dichloromethane-d_2 showed full conversion of triethyloxonium tetrafluoroborate. Subsequently, dichloromethane was removed at –20 °C under reduced pressure (3 mbar). To the resulted white slurry, anhydrous diethyl ether (10 mL) was added dropwise at –78 °C, and the suspension was filtered at –78 °C under Ar. The filtrate was concentrated under reduced pressure (3 mbar) at –20 °C to remove diethyl ether. Then anhydrous toluene (10 mL) was added dropwise at –78 °C and the product was obtained as a toluene solution and was stored at –78 °C under Ar. The concentration of the product in toluene was determined by ^{1}H NMR with internal standard (benzyl methyl ether/dibromomethane) within 10 minutes.

<u>Note:</u>

1. The obtained product is highly sensitive to oxygen, moisture and temperature.
2. The product solution can be decomposed at room temperature (80% was decomposed at room temperature within 4 hours).
3. It should be handled carefully under Ar atmosphere and low temperature (–20 °C).

^{1}H NMR (501 MHz, Tol-d_8) δ 4.14 (q, *J* = 7.0 Hz, 2H), 1.68 (q, *J* = 1.1 Hz, 3H), 1.61 (q, *J* = 1.0 Hz, 3H), 0.98 (t, *J* = 6.9 Hz, 3H).

Ethyl methyleneazinate (2e):

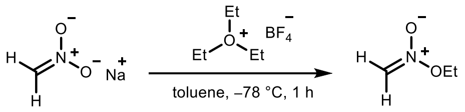

To a flame dried Schlenk flask under Ar and equipped with a magnetic stirring bar, sodium methyleneazinate (996 mg, 12 mmol, 1.2 equiv.) and anhydrous toluene (6 mL) were added. The suspension was cooled to –78 °C and the triethyloxonium tetrafluoroborate (10 mmol, 1.0 equiv.) was added. The reaction mixture was stirred at –78 °C for 1 hour. Subsequently, the suspension was warmed to room temperature and filtered under Ar within 15 minutes

(including warm-up and filtration). The product (filtrate) was obtained as a toluene solution and was stored at –78 °C under Ar. The concentration of the product in toluene was determined by ^{1}H NMR with internal standard (benzyl methyl ether) within 10 minutes.

Note:

1. The obtained product is highly sensitive to oxygen, moisture and temperature.
2. The product solution can be decomposed at room temperature (80% was decomposed at room temperature within few hours).
3. It should be handled carefully under Ar atmosphere and low temperature.
4. The product has poor solubility in toluene at low temperatures. The product (filtrate) will precipitate at –78 °C as a white flocculent solid.

^{1}H NMR (501 MHz, Tol-d_8) δ 5.19 (d, *J* = 6.8 Hz, 1H), 4.92 (d, *J* = 6.8 Hz, 1H), 3.96 (q, *J* = 6.9 Hz, 2H), 0.90 (t, *J* = 7.0 Hz, 3H).

Ethyl cyclohexylideneazinate (2f):

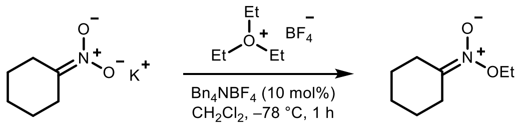

To a flame dried Schlenk flask under Ar and equipped with a magnetic stirring bar, potassium cyclohexylideneazinate (650 mg, 3.9 mmol, 1.25 equiv.) and anhydrous dichloromethane (5 mL) were added. The suspension was cooled to –78 °C and the triethyloxonium tetrafluoroborate solution (2 M in anhydrous dichloromethane, 1.6 mL, 3.1 mmol 1.0 equiv.) was added dropwise followed by tetrabenzylammonium tetrafluoroborate solution (0.8 M in anhydrous dichloromethane, 0.38 mL, 0.31 mmol, 0.1 equiv.). The reaction mixture was stirred at –78 °C for 1 hour. The crude NMR in dichloromethane-d_2 showed full conversion of triethyloxonium tetrafluoroborate. Subsequently, dichloromethane was removed at –20 °C under reduced pressure (3 mbar). To the resulted white slurry, anhydrous diethyl ether (10 mL) was added dropwise at –78 °C, and the suspension was filtered at –78 °C under Ar. The filtrate was concentrated under reduced pressure (3 mbar) at –20 °C to remove diethyl ether. Then anhydrous toluene (3 mL) was added dropwise at –78 °C and the product was obtained as a toluene solution and was stored at –78 °C under Ar. The concentration of the product in toluene was determined by ^{1}H NMR with internal standard (benzyl methyl ether/dibromomethane) within 10 minutes.

Note:

1. The obtained product is highly sensitive to oxygen, moisture and temperature.
2. The product solution can be decomposed at room temperature (fully decomposed at room temperature within 4 hours in NMR tube).
3. It should be handled carefully under Ar atmosphere and low temperature (–20 °C).

^{1}**H NMR** (501 MHz, CD_2Cl_2) δ 4.23 (q, *J* = 7.0 Hz, 2H), 2.60 (t, *J* = 6.4 Hz, 2H), 2.52 (t, *J* = 6.5 Hz, 2H), 1.71–1.52 (m, 6H), 1.22 (t, *J* = 7.0 Hz, 3H).

Methyl propan-2-ylideneazinate (2h):

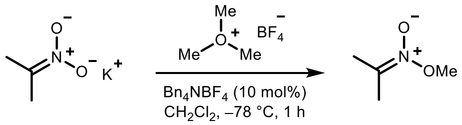

To a flame dried Schlenk flask under Ar and equipped with a magnetic stirring bar, potassium propan-2-ylideneazinate (763 mg, 6 mmol, 1.2 equiv.) and anhydrous dichloromethane (6 mL) were added. The suspension was cooled to –78 °C and the trimethyloxonium tetrafluoroborate solution (2 M in anhydrous dichloromethane, 2.5 mL, 5 mmol 1.0 equiv.) was added dropwise followed by tetrabenzylammonium tetrafluoroborate solution (0.8 M in anhydrous dichloromethane, 0.625 mL, 0.5 mmol, 0.1 equiv.). The reaction mixture was stirred at –78 °C for 1 hour. The crude NMR in dichloromethane-d_2 showed full conversion of trimethyloxonium tetrafluoroborate. Subsequently, dichloromethane was removed at –20 °C under reduced pressure (3 mbar). To the resulted white slurry, anhydrous diethyl ether (10 mL) was added dropwise at –78 °C, and the suspension was filtered at –78 °C under Ar. The filtrate was concentrated under reduced pressure (3 mbar) at –20 °C to remove diethyl ether. Then anhydrous toluene (10 mL) was added dropwise at –78 °C and the product was obtained as a toluene solution and was stored at –78 °C under Ar. The concentration of the product in toluene was determined by ^{1}H NMR with internal standard (benzyl methyl ether/dibromomethane) within 10 minutes.

Note:

1. The obtained product is highly sensitive to oxygen, moisture and temperature.
2. The product solution can be decomposed at room temperature (80 % was decomposed at room temperature within 4 hours).
3. It should be handled carefully under Ar atmosphere and low temperature (–20 °C).

^{1}**H NMR** (501 MHz, Tol-d_8) δ 3.54 (s, 3H), 1.66–1.63 (m, 3H), 1.58–1.56 (m, 3H).

^{1}**H NMR** (501 MHz, CD_2Cl_2) δ 3.74 (s, 3H), 2.02 (s, 3H), 1.99–1.94 (m, 3H).

Triisobutylsilyl methyleneazinate (2l):

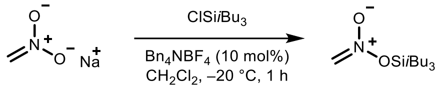

To a flame dried Schlenk flask under Ar and equipped with a magnetic stirring bar, sodium methyleneazinate (830 mg, 10 mmol, 2 equiv.) and anhydrous dichloromethane (6 mL) were added. The suspension was cooled to –78 °C and the chlortriisobutylsilane (1.34 mL, 5 mmol 1.0 equiv.) was added dropwise followed by tetrabenzylammonium tetrafluoroborate solution (0.8 M in anhydrous dichloromethane, 0.625 mL, 0.5 mmol, 0.1 equiv.). The reaction mixture was stirred at –20 °C for 1 hour. The crude NMR in toluene-d_8 showed full conversion of chlortriisobutylsilane. Subsequently, dichloromethane was removed at –20 °C under reduced pressure (0.3 mbar). To the resulted white slurry, anhydrous diethyl ether (10 mL) was added dropwise at –78 °C, and the suspension was filtered at –20 °C under Ar. The filtrate was concentrated under reduced pressure (0.3 mbar) at –20 °C to remove diethyl ether. Then anhydrous toluene (10 mL) was added dropwise at –78 °C and the product was obtained as a toluene solution and was stored at –78 °C under Ar. The concentration of the product in toluene was determined by ^{1}H NMR with internal standard (benzyl methyl ether/dibromomethane) within 10 minutes.

Note:

1. The obtained product is highly sensitive to oxygen, moisture and temperature.
2. The product solution can be decomposed at room temperature (fully decomposed at room temperature within 24 hours in NMR tube).
3. It should be handled carefully under Ar atmosphere and low temperature (–20 °C).

^{1}H NMR (501 MHz, Tol-d_8) δ 5.14 (s, 2H), 1.99 (dp, *J* = 12.9, 6.5 Hz, 3H), 1.05 (dd, *J* = 6.6, 1.1 Hz, 18H), 0.93 (dd, *J* = 7.0, 1.4 Hz, 6H).

Methyldiphenylsilyl methyleneazinate (2m):

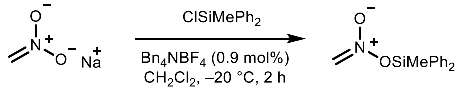

To a flame dried Schlenk flask under Ar and equipped with a magnetic stirring bar, sodium methyleneazinate (830 mg, 10 mmol, 2 equiv.) and anhydrous dichloromethane (6 mL) were added. The suspension was cooled to –78 °C and the chloro(methyl)diphenylsilane (0.84 mL, 5 mmol 1.0 equiv.) was added dropwise followed by tetrabenzylammonium tetrafluoroborate solution (0.8 M in anhydrous dichloromethane, 0.056 mL, 0.005 mmol, 0.01 equiv.). The reaction mixture was stirred at –20 °C for 2 hour. The crude NMR in toluene-d_8 showed >90% conversion of chloro(methyl)diphenylsilane. Subsequently, anhydrous toluene (10 mL) was added dropwise at –78 °C and the

suspension was filtered at 0 °C under Ar within 10 min. Then the filtrate was concentrated under reduced pressure (0.3 mbar) at –20 °C to remove dichloromethane. The product was obtained as a toluene solution and was stored at –78 °C under Ar. The concentration of the product in toluene was determined by ^{1}H NMR with internal standard (benzyl methyl ether/dibromomethane) within 10 minutes.

Note:

1. The obtained product is highly sensitive to oxygen, moisture and temperature.
2. The product solution can be decomposed at room temperature (fully decomposed at room temperature within 4 hours in NMR tube).
3. It should be handled carefully under Ar atmosphere and low temperature (–20 °C).

^{1}H NMR (501 MHz, Tol-d_8) δ 7.68–7.62 (m, 4H), 7.20–7.12 (m, 6H), 5.03 (s, 2H), 0.86 (s, 3H).

5,6-dihydro-4*H*-1,2-oxazine 2-oxide (2n):

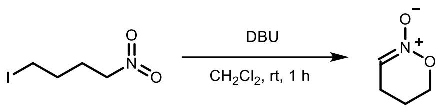

To a flame dried Schlenk flask under Ar and equipped with a magnetic stirring bar, 1-iodo-4-nitrobutane (250 mg, 1.1 mmol, 1.0 equiv.), anhydrous dichloromethane (3 mL) and diazabicycloundecen (0.15 mL, 1.0 mmol, 0.95equiv.) were added at 0 °C. The reaction mixture was stirred at rt for 1 hour. Subsequently, dichloromethane was removed at –20 °C under reduced pressure (3 mbar). Then anhydrous diethyl ether (10 mL) was added dropwise, and the suspension was filtered at –20 °C under Ar. The filtrate was concentrated under reduced pressure (3 mbar) at –20 °C to remove diethyl ether. Then anhydrous toluene (2 mL) was added dropwise at –20 °C and the product was obtained as a toluene solution and was stored at –78 °C under Ar. The concentration of the product in toluene was determined by ^{1}H NMR with internal standard (benzyl methyl ether/dibromomethane).

^{1}H NMR (501 MHz, CD_2Cl_2) δ 6.20 (t, *J* = 4.1 Hz, 1H), 4.37–4.34 (m, 2H), 2.43 (td, *J* = 6.8, 4.1 Hz, 2H), 1.95–1.88 (m, 2H).

Reaction Development with Different Catalysts

Supplementary Table 1. Investigation of different acid catalysts for the reaction between nitronates (**2**) and silyl ketene acetals (**3**).

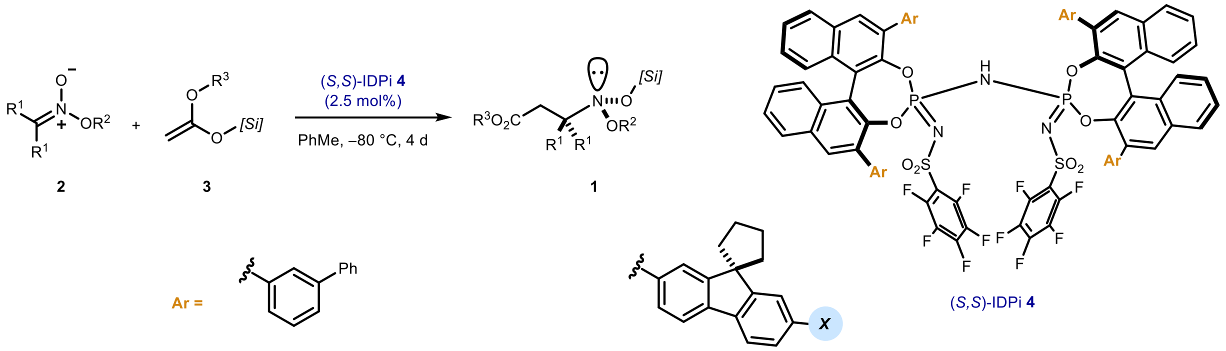

		X = H	X = Me	X = *t*Bu	X = Adam
**1d**	49%, 84:18 er	95%, 89.7:10.3 er	95%, 91:9 er	95%, 95.5:4.5 er	95%, 96.6:3.4 er
**1e**	95%, 92.5:7.5 er	95%, 72:28 er	N.A.	95%, 80:20 er	N.A.
**1f**	95%, 81:19 er	N.A.	95%, 66.3:33.7 er	95%, 65.5:34.5 er	95%,72.5:27.5 er
**1g**	30%, 78:22 er	95%, 83:17 er	N.A.	95%, 89:11 er	95%, 89.4:10.6 er
**1h**	N.A.	N.A.	N.A.	N.A.	90%, 86.2:13.8 er
**1i**	12%, 81.6:18.3 er	77%, 84.3:15.7 er	N.A.	57%, 85.5:14.5 er	63%, 87:13 er
**1j (–60 °C)**	trace, 53.3:46.7 er	N.A.	N.A.	N.A.	trace, 53.1:46.9 er
**1k (–60 °C)**	95%, 94.8:5.2 er	95%, 84.2:15.8 er	N.A.	N.A.	N.A.
**1l**	N.A.	N.A.	N.A.	N.A.	N.A.
**1m (–20 °C)**	trace, 52:48 er	N.A.	N.A.	N.A.	N.A.

unsuccessful nitonate and SKA

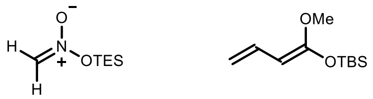

Reactions conducted on 0.025 mmol scale. Conversion and yields were determined by ^{1}H NMR using internal standard. Er were determined by HPLC. N.A., Not available.

General Procedure for the Addition of Silyl Ketene Acetals to Ethyl Nitronates (GP)

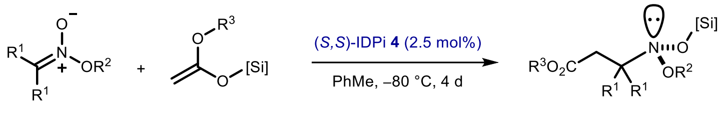

2 3 1

In an oven-dried glass vial, the respective IDPi catalyst **4** (0.025 equiv., 2.5 mol%) was dissolved in toluene (375 µL, 0.25M with respect to the nitronate) and reacted with the silyl ketene acetal (2.0 equiv., 0.5 mmol) under Ar for 5 min at room temperature to give the active silylium IDPi species. The vial was transferred to a cryostat at –80 °C temperature and stirred for 10 min, a stock solution of the nitronate (1.0 equiv., 0.25 mmol, 0.40M in toluene, 625 µL) was added dropwise over the cold glass surface and the resulting reaction mixture was stirred for 4 days. Subsequently, the reaction was quenched by the addition of triethylamine and MeOH (200 µL/mmol of silyl nitronate), stirred for 10 min at –80 °C and was then allowed to warm up to room temperature. The crude reaction mixtures were directly purified *via* flash column chromatography (4% Et_2O in hexanes, silica gel) within 45 min to give the products as colorless oils.

Note:

1. The obtained product is highly sensitive to temperature (all the steps: column chromatography, concentration of the fractions and HPLC sample preparation should be performed <25 °C).

(*S*)-methyl 3-(((tert-butyldimethylsilyl)oxy)(ethoxy)amino)-3-methylbutanoate (1d): Prepared according to GP using catalyst **4k**, 68% yield as colorless oil.

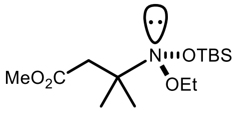

1d

^{1}H NMR (501 MHz, CD_2Cl_2) δ 3.96 (dq, *J* = 9.0, 7.2 Hz, 1H), 3.68 (dq, *J* = 9.1, 7.2 Hz, 1H), 3.62 (s, 3H), 2.61 (d, *J* = 3.9 Hz, 2H), 1.18 (s, 3H), 1.16 (s, 3H), 1.11 (t, *J* = 7.1 Hz, 3H), 0.92 (s, 9H), 0.20 (s, 3H), 0.15 (s, 3H).

^{13}C NMR (126 MHz, CD_2Cl_2) δ 173.1, 68.9, 67.6, 51.6, 40.2, 26.5, 23.7, 23.3, 18.6, 14.0, –3.0, –4.1.

HRMS *m/z* (ESI): calcd. for $C_{14}H_{31}NO_4SiNa$ $[M+Na]^+$: 328.1915; found: 328.1912.

The enantiomeric ratio was measured by HPLC analysis: 1-dimension using 250 mm Multokrom 3-100Si , 4.6 mm i.D, *n*-heptane/*i*-propanol = 99:1, flow rate 1.0 mL/min, λ = 220 nm, 298 K, t_R = 3.60 and then 2-dimension using 150 mm Chiralcel OD-3, 4.6 mm i.D, *n*-heptane = 100, flow rate = 1.0 mL/min, λ = 220 nm, 298 K, t_R = 3.27 min (minor) and t_R = 3.56 min (major). er = 96.3:3.4. $[\alpha]_D^{25}$ = –19.8 (*c* 1.23, CH_2Cl_2).

(*S*)-methyl 3-(((tert-butyldimethylsilyl)oxy)(ethoxy)amino)propanoate (1e): Prepared according to GP using catalyst **4h**, with 95% NMR yield.

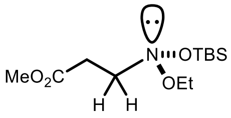

1e

^{1}H NMR (501 MHz, Tol) δ 3.77 (dq, *J* = 9.4, 7.1 Hz, 1H), 3.65 (dq, *J* = 9.4, 7.1 Hz, 1H), 3.37–3.24 (m, 5H), 2.49 (ddd, *J* = 16.2, 7.7, 6.5 Hz, 1H), 2.41 (dt, *J* = 16.2, 6.2 Hz, 1H), 1.02 (t, *J* = 7.1 Hz, 3H), 0.96 (s, 9H), 0.19 (s, 3H), 0.16 (s, 3H).

HRMS *m/z* (ESI): calcd. for $C_{12}H_{27}NO_4SiNa$ $[M+Na]^+$: 300.1602; found: 300.1598.

The enantiomeric ratio was measured by HPLC analysis: 1-dimension using 100 mm RX-SIL, 4.6 mm i.D., *n*-heptane/*i*-propanol = 99.5:0.5, flow rate 1.0 mL/min, λ = 210 nm, 308 K, t_R = 1.56 and then 2-dimension using 150 mm Chiralcel OD-3, 4.6 mm i.D, *n*-heptane = 99.8:0.2, flow rate = 1.0 mL/min, λ = 210 nm, 298 K, t_R = 3.73 min (major) and t_R = 4.13 min (minor). er = 91.9:9.1.

(*S*)-methyl 2-(1-(((tert-butyldimethylsilyl)oxy)(ethoxy)amino)cyclohexyl)acetate (1f): Prepared according to GP using catalyst **4h**, 64% yield as colorless oil.

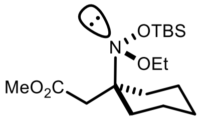

1f

^{1}H NMR (501 MHz, CD_2Cl_2) δ 4.03–3.93 (m, 1H), 3.74–3.66 (m, 1H), 3.60 (s, 3H), 2.70–2.58 (m, 2H), 1.71–1.58 (m, 6H), 1.42 (d, *J* = 10.5 Hz, 4H), 1.10 (t, *J* = 7.1 Hz, 3H), 0.91 (s, 9H), 0.21 (s, 3H), 0.17 (s, 3H).

^{13}C NMR (126 MHz, CD_2Cl_2) δ 173.6, 70.2, 68.6, 51.5, 35.8, 33.5, 33.1, 26.5, 26.3, 22.9, 22.6, 18.7, 14.0, –3.2, –4.1.

HRMS *m/z* (ESI): calcd. for $C_{17}H_{35}NO_4SiNa$ $[M+Na]^+$: 368.22249; found: 368.22276.

The enantiomeric ratio was measured by HPLC analysis: 1-dimension using 250 mm PVA-SIL, 4.6 mm i.D., n-Heptan i-Propanol = 99.5:0.5 flow rate 1.0 mL/min, λ = 210 nm, 298 K, t_R = 3.79 min and then 2-dimension using 150 mm Chiralcel OD-3, 4.6 mm i.D., *n*-heptane = 100, flow rate = 1.0 mL/min, λ = 210 nm, 298 K, t_R = 7.79 min (minor) and t_R = 8.26 min (major). er = 81.2:18.8. $[\alpha]_D^{25}$ = –28.727 (*c* 0.55, heptane, er = 74:26).

(*S*)-ethyl 3-(((tert-butyldimethylsilyl)oxy)(ethoxy)amino)-3-methylbutanoate (1g): Prepared according to GP using catalyst **4k**, 38% yield as colorless oil.

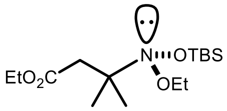

1g

^{1}H NMR (501 MHz, CD_2Cl_2) δ 4.08 (qd, *J* = 7.1, 1.3 Hz, 2H), 3.96 (dq, *J* = 9.0, 7.1 Hz, 1H), 3.69 (dq, *J* = 9.0, 7.2 Hz, 1H), 2.58 (s, 2H), 1.24 (t, *J* = 7.2 Hz, 3H), 1.19 (s, 3H), 1.17 (s, 3H), 1.11 (t, *J* = 7.2 Hz, 3H), 0.92 (s, 9H), 0.20 (s, 3H), 0.15 (s, 3H).

^{13}C NMR (126 MHz, CD_2Cl_2) δ 172.6, 68.9, 67.6, 60.5, 40.7, 26.4, 23.7, 23.1, 18.6, 14.6, 13.9, –3.0, –4.1.

HRMS *m/z* (ESI): calcd. for $C_{15}H_{33}NO_4SiNa$ $[M+Na]^+$: 342.2071; found: 342.2069.

The enantiomeric ratio was measured by HPLC analysis: 150 mm Chiralcel OD-3, 4.6 mm i.D., *n*-heptane = 100, flow rate = 1.0 mL/min, λ = 220 nm, 288 K, t_R = 3.24 min (minor) and t_R = 3.42 min (major). er = 87.2:12.8. $[\alpha]_D^{25}$ = –16.7 (*c* 0.30, CH_2Cl_2).

(*S*)-isobutyl 3-(((tert-butyldimethylsilyl)oxy)(ethoxy)amino)-3-methylbutanoate (1h): Prepared according to GP using catalyst **4k**, 86% yield as colorless oil.

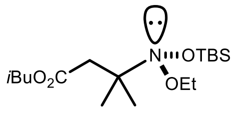

1h

^{1}H NMR (501 MHz, CD_2Cl_2) δ 3.96 (dq, *J* = 9.0, 7.1 Hz, 1H), 3.81 (d, *J* = 6.2 Hz, 2H), 3.69 (dq, *J* = 9.0, 7.1 Hz, 1H), 2.61 (s, 2H), 1.91 (dp, *J* = 13.4, 6.7 Hz, 1H), 1.19 (s, 3H), 1.17 (s, 3H), 1.11 (t, *J* = 7.1 Hz, 3H), 0.94 (s, 3H), 0.93 (s, 3H), 0.92 (s, 9H), 0.20 (s, 3H), 0.16 (s, 3H).

^{13}C NMR (126 MHz, CD_2Cl_2) δ 172.7, 70.9, 68.9, 67.6, 40.6, 28.3, 26.5, 23.7, 23.3, 19.5, 18.6, 14.0, –3.0, –4.1.

HRMS *m/z* (ESI): calcd. for $C_{17}H_{37}NO_4SiNa$ $[M+Na]^+$: 370.2384; found: 370.2384.

The enantiomeric ratio was measured by HPLC analysis: 150 mm Chiralcel OD-3, 4.6 mm i.D., *n*-heptane = 100, flow rate = 1.0 mL/min, λ = 220 nm, 288 K, t_R = 3.03 min (minor) and t_R = 3.16 min (major). er = 86.2:13.8. $[\alpha]_D^{25}$ = –14.2 (*c* 0.41, CH_2Cl_2).

(*S*)-benzyl 3-(((tert-butyldimethylsilyl)oxy)(ethoxy)amino)-3-methylbutanoate (1i): Prepared according to GP using catalyst **4l**, 66% yield as colorless oil.

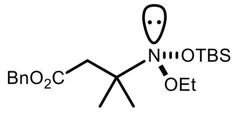

1i

^{1}H NMR (501 MHz, CD_2Cl_2) δ 7.39–7.29 (m, 5H), 5.09 (d, *J* = 5.4 Hz, 2H), 3.95 (dq, *J* = 9.0, 7.1 Hz, 1H), 3.68 (dq, *J* = 9.1, 7.2 Hz, 1H), 2.67 (d, *J* = 1.9 Hz, 2H), 1.19 (s, 3H), 1.17 (s, 3H), 1.10 (t, *J* = 7.1 Hz, 3H), 0.92 (s, 9H), 0.20 (s, 3H), 0.15 (s, 3H).

^{13}C NMR (126 MHz, CD_2Cl_2) δ 172.5, 137.0, 129.0, 128.7, 128.6, 68.9, 67.7, 66.5, 40.5, 26.5, 23.8, 23.3, 18.6, 14.0, –3.0, –4.1.

HRMS *m/z* (ESI): calcd. for $C_{20}H_{35}NO_4SiNa$ $[M+Na]^+$: 404.2227; found: 404.2226.

The enantiomeric ratio was measured by HPLC analysis: 1-dimension using 250 mm Multokrom 3-100Si , 4.6 mm i.D, *n*-heptane/*i*-propanol = 99:1, flow rate 1.0 mL/min, λ = 210 nm, 298 K, t_R = 3.35. and then 2-dimension using 150 mm Chiralcel OZ-3, 4.6 mm i.D, *n*-heptane/*i*-propanol = 99.9:0.1, flow rate = 1.0 mL/min, λ = 210 nm, 298 K, t_R = 6.38 min (major) and t_R = 7.51 min (minor). er = 95.9:4.1. $[\alpha]_D^{25}$ = –18.7 (*c* 0.86, CH_2Cl_2).

(*S*)-methyl 3-(((tert-butyldimethylsilyl)oxy)(methoxy)amino)-3-methylbutanoate (1j): Prepared according to GP using catalyst **4m**, 93% yield as colorless oil.

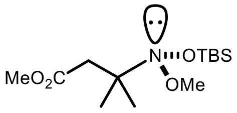

1j

^{1}H NMR (501 MHz, CD_2Cl_2) δ 3.62 (s, 3H), 3.58 (s, 3H), 2.59 (q, *J* = 13.7 Hz, 2H), 1.17 (d, *J* = 7.1 Hz, 6H), 0.92 (s, 9H), 0.22 (s, 3H), 0.16 (s, 3H).

^{13}C NMR (126 MHz, CD_2Cl_2) δ 173.0, 67.9, 61.5, 51.7, 40.3, 26.4, 23.7, 23.0, 18.5, –3.0, –4.1.

HRMS *m/z* (ESI): calcd. for $C_{13}H_{29}NO_4SiNa$ $[M+Na]^+$: 314.1758; found: 314.1756.

The enantiomeric ratio was measured by HPLC analysis: 150 mm Chiralcel OD-3, 4.6 mm i.D., *n*-heptane = 100, flow rate = 1.0 mL/min, λ = 220 nm, 288 K, t_R = 4.39 min (major) and t_R = 5.90 min (minor). er = 92.8:7.2. $[\alpha]_D^{25}$ = +8.1 (*c* 0.82, CH_2Cl_2).

(*S*)-methyl 3-((((2,3-dimethylbutan-2-yl)dimethylsilyl)oxy)(ethoxy)amino)-3-methylbutanoate (1e): Prepared according to GP using catalyst **4h**, 80% yield as colorless oil.

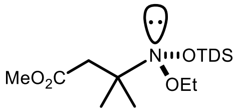

1e

^{1}H NMR (501 MHz, CD_2Cl_2) δ 4.0 (dq, *J* = 9.0, 7.1 Hz, 1H), 3.7 (dq, *J* = 9.0, 7.2 Hz, 1H), 3.6 (s, 3H), 2.7–2.6 (m, 2H), 1.7–1.6 (m, 1H), 1.2 (s, 3H), 1.2 (s, 3H), 1.1 (t, *J* = 7.2 Hz, 3H), 1.0–0.8 (m, 12IIH), 0.2 (s, 3H), 0.2 (s, 3H).

^{13}C NMR (126 MHz, CD_2Cl_2) δ 173.1, 68.8, 67.6, 51.6, 40.4, 34.7, 25.9, 23.6, 23.4, 21.1, 20.6, 19.1, 18.8, 14.0, –1.3, –2.1.

HRMS *m/z* (ESI): calcd. for $C_{16}H_{35}NO_4SiNa$ $[M+Na]^+$: 356.2228; found: 356.2224.

The enantiomeric ratio was measured by HPLC analysis: 1-dimension using 100 mm RX-SIL , 4.6 mm i.D., *n*-heptane/*i*-propanol = 99.9:0.1, flow rate 1.0 mL/min, λ = 210 nm, 298 K, t_R = 3.08 and then 2-dimension using 150 mm Chiralcel OD-3, 4.6 mm i.D, *n*-heptane = 100, flow rate = 1.0 mL/min, λ = 210 nm, 298 K, t_R = 3.18 min (minor) and t_R = 3.47 min (major). er = 96.8:3.2. $[\alpha]_D^{25}$ = –19.7 (*c* 1.00, CH_2Cl_2).

(*S*)-methyl 3-(((tert-butyldimethylsilyl)oxy)((triisobutylsilyl)oxy)amino)propanoate (1l): Prepared according to GP using catalyst **4d**, 36% yield as colorless oil.

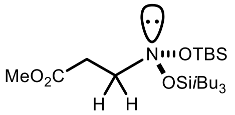

1l

^{1}H NMR (501 MHz, CD_2Cl_2) δ 3.65 (s, 3H), 3.26 (td, *J* = 7.3, 2.0 Hz, 2H), 2.68 (t, *J* = 7.1 Hz, 2H), 1.89–1.81 (m, 3H), 0.96 (s, 9H), 0.95 (s, 9H), 0.90 (d, *J* = 2.8 Hz, 9H), 0.72 (dd, *J* = 6.9, 1.8 Hz, 6H), 0.21 (s, 3H), 0.15 (s, 3H).

^{13}C NMR (126 MHz, CD_2Cl_2) δ 173.3, 62.5, 51.9, 29.2, 26.8, 26.8, 26.3, 25.9, 24.7, 18.2, –3.5, –4.1.

HRMS *m/z* (ESI): calcd. for $C_{22}H_{49}NO_4Si_2Na$ $[M+Na]^+$: 470.3092; found: 470.3092.

The enantiomeric ratio was measured by HPLC analysis: 150 mm Chiralcel OZ-3R, 4.6 mm i.D, acetonitrile/water = 70:30, flow rate = 0.5 mL/min, λ = 220 nm, 298 K, t_R = 22.37 min (minor) and t_R = 23.24 min (major). er = 67.7:32.3.$[\alpha]_D^{25}$ = –7.7 (*c* 0.44, CH_2Cl_2).

(*R*)-methyl 3-(((tert-butyldimethylsilyl)oxy)((methyldiphenylsilyl)oxy)amino)propanoate (1m): Prepared according to GP using catalyst **4d**, 47% yield as colorless oil.

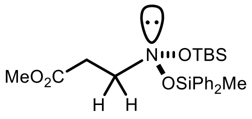

1m

^{1}H NMR (501 MHz, CD_2Cl_2) δ 7.64–7.55 (m, 4H), 7.44–7.34 (m, 6H), 3.60 (s, 3H), 3.28 (t, *J* = 6.8 Hz, 2H), 2.63 (td, *J* = 6.9, 3.2 Hz, 2H), 0.84 (s, 9H), 0.76 (s, 3H), 0.00 (s, 3H), -0.24 (s, 3H).

^{13}C NMR (126 MHz, CD_2Cl_2) δ 173.0, 136.3, 136.1, 135.4, 135.1, 130.5, 130.5, 128.3, 128.3, 62.6, 51.9, 29.8, 26.2, 18.1, –2.7, –4.4, –4.6.

HRMS *m/z* (ESI): calcd. for $C_{23}H_{35}NO_4Si_2Na$ $[M+Na]^+$: 468.1997; found: 468.1997.

The enantiomeric ratio was measured by HPLC analysis: 150 mm Chiralcel OJ-3R, 4.6 mm i.D, acetonitrile/water = 50:50, flow rate = 1.0 mL/min, λ = 220 nm, 298 K, t_R = 30.21 min (minor) and t_R = 32.59 min (major). er = 75:25. $[\alpha]_D^{25}$ = +8.4 (*c* 1.17, CH_2Cl_2).

Configurational Stability Study

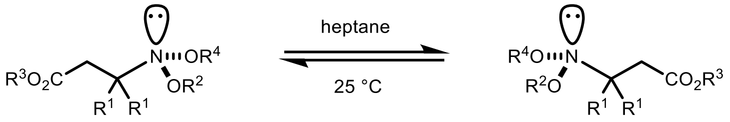

The configurational stability of amine products were studied by measuring the enantiomeric ratio over time (every 2 hours) at 25 °C in heptane using HPLC methods.

The obtained data was plotted as ee vs time and ln(1/ee) vs time, and the racemization rate constant k_{rac} could be obtained *via* the following equations:

eq. 1: $$ee = ee_0 \times e^{-k_{rac}t}$$

eq. 2: $$\ln(1/ee) = k_{rac}t + \ln(1/ee_0)$$

k_{rac}: racemization rate constant

t: time

ee: enantiomeric excess over time

ee_0: the initial enantiomeric excess (when t = 0)

e: mathematical constant

The half-life ($t_{1/2}$) and activation energy ($\Delta G_{25\,°C}$) of amine products were obtained *via* the following equations:

eq. 3: $$t_{1/2} = \frac{\ln 2}{k_{rac}}$$

eq. 4: $$\Delta G_{25\,°C} = RT\ln(k_bT/kh)$$

$R = 1.99 \times 10^{-3}$ kcal/mol

T = 25 °C = 298 K

$K_b = 1.381 \times 10^{-23}$ J/K

$h = 6.626 \times 10^{-34}$ Js

$k = k_{rac}/2$

Supplementary Table 2. Enantiomeric excess over time for **1d**: ***Run 1:***

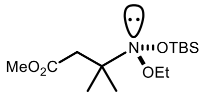

k_{rac} = 0.0156 h^{-1}, $t_{1/2}$ = 44.4 h, $\Delta G_{25\ °C}$ = 25.20 kcal/mol

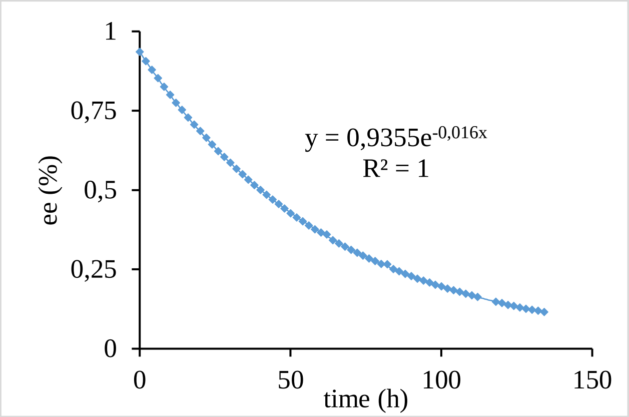

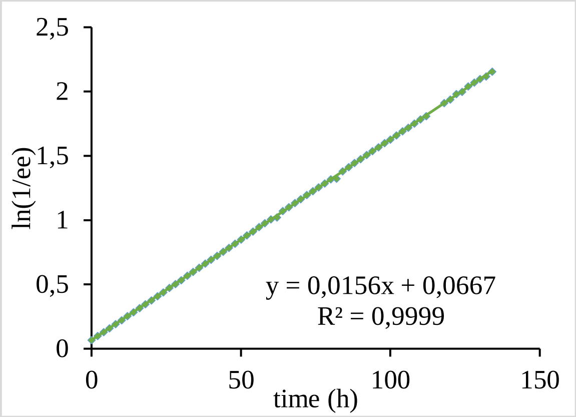

time	ee	ln(1/ee)
0	0.935660795	0.066502266
2	0.906468132	0.098199405
4	0.878891245	0.129094114
6	0.853020154	0.158972105
8	0.826207303	0.190909565
10	0.80093872	0.221970839
12	0.775059917	0.25481494
14	0.752737569	0.284038626
16	0.728961749	0.316134019
18	0.707016015	0.346701961
20	0.68599685	0.376882243
22	0.66489609	0.408124506
24	0.644479928	0.4393116
26	0.623334213	0.472672446
28	0.604982944	0.502555014
30	0.586569968	0.533463321
32	0.567378694	0.566728308
34	0.55012662	0.597606809
36	0.53268537	0.62982433
38	0.51605434	0.661543209
40	0.500703198	0.691741773
42	0.485859758	0.72183526
44	0.470140071	0.754724606
46	0.456001456	0.785259276
48	0.44181182	0.816871235
50	0.427197168	0.850509621
52	0.414344252	0.881058124
54	0.401874589	0.911615206
56	0.388493357	0.945479209
58	0.37687542	0.975840597
60	0.365948201	1.005263482

62	0.360322791	1.020755008
64	0.34258414	1.071237988
66	0.332561645	1.100930039
68	0.322195247	1.132597559
70	0.312057912	1.164566493
72	0.302428706	1.195909711
74	0.293594937	1.225554228
76	0.284683119	1.256378582
78	0.276222639	1.286548075
80	0.267707143	1.317861648
82	0.26621224	1.323461395
84	0.251525474	1.380211008
86	0.243787875	1.411456796
88	0.235957768	1.44410244
90	0.228677706	1.475441665
92	0.221255763	1.508435947
94	0.21493854	1.537403151
96	0.20856352	1.567511633
98	0.201936552	1.599801728
100	0.196427338	1.627462696
102	0.190114243	1.660130111
104	0.184267449	1.69136705
106	0.17921712	1.719157247
108	0.173368724	1.752334602
110	0.168065574	1.783401056
112	0.163685382	1.809809098
114	no solvent in sample	
116	no solvent in sample	
118	0.147884855	1.911321312
120	0.144113799	1.937152021
122	0.138148475	1.97942627
124	0.135563595	1.998314413
126	0.129853201	2.041350687
128	0.126066671	2.070944379
130	0.122873633	2.096598825
132	0.120167127	2.118871782
134	0.115913098	2.154914524

Supplementary Table 3. Enantiomeric excess over time for **1d**: ***Run 2:***

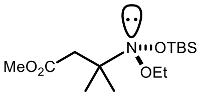

Repeating experiment: k_{rac} = 0.0153 h^{-1}, $t_{1/2}$ = 45.3 h, $\Delta G_{25\,°C}$ = 25.21 kcal/mol

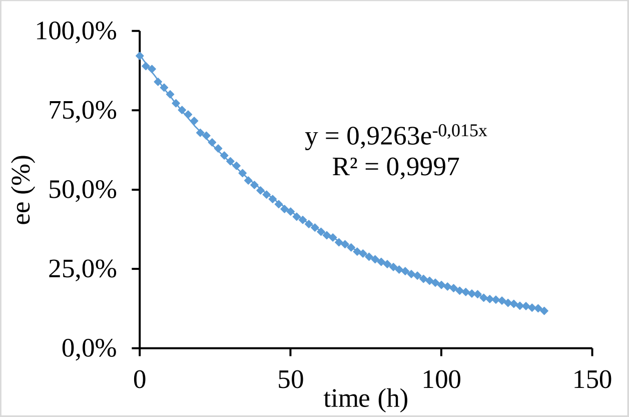

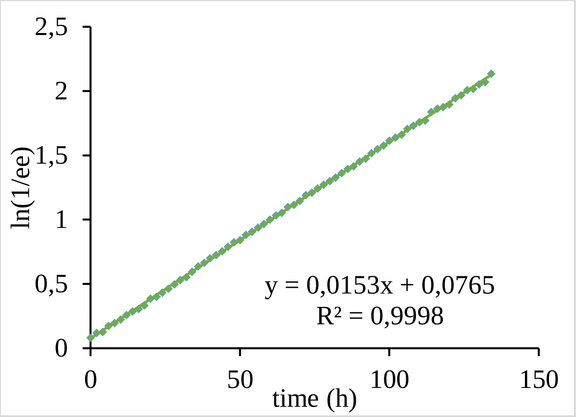

time	ee	ln(1/ee)
0	0.921380361	0.081882341
2	0.888957656	0.117705675
4	0.880593932	0.127158677
6	0.840022655	0.174326417
8	0.821104699	0.197104651
10	0.800655822	0.22232411
12	0.77182448	0.258998112
14	0.750734003	0.286703881
16	0.737252642	0.304824648
18	0.716410638	0.33350176
20	0.67935171	0.386616303
22	0.670320706	0.399999016
24	0.648522471	0.433058624
26	0.630032865	0.461983294
28	0.607749227	0.497992938
30	0.589284359	0.528846429
32	0.575637365	0.552277391
34	0.552198564	0.593847581
36	0.529049837	0.636672641
38	0.515082313	0.66342856
40	0.497375377	0.698410251
42	0.48468725	0.724251441
44	0.46995366	0.755121185
46	0.454439122	0.78869132
48	0.43859998	0.824167488
50	0.430586544	0.842606945
52	0.414191378	0.881427145
54	0.40420113	0.905842678
56	0.391473536	0.937837361

58	0.380200967	0.967055306
60	0.367601552	1.000755666
62	0.355892363	1.033126945
64	0.348646171	1.053697708
66	0.333731329	1.097419015
68	0.327556199	1.116095638
70	0.317989701	1.145736284
72	0.304303601	1.189729388
74	0.298458336	1.209124933
76	0.288693531	1.2423896
78	0.279973015	1.273062057
80	0.272532679	1.299996749
82	0.264940454	1.32825018
84	0.256352533	1.361201701
86	0.248123536	1.393828528
88	0.24302456	1.41459277
90	0.233936473	1.452705681
92	0.228824544	1.474799754
94	0.219301054	1.517309816
96	0.212329658	1.549615223
98	0.206641064	1.576771984
100	0.199255297	1.613168379
102	0.194099032	1.639386777
104	0.189872228	1.661403915
106	0.181731952	1.705222471
108	0.177278899	1.730031087
110	0.172296391	1.75853908
112	0.170072619	1.771529763
114	0.159158741	1.837853206
116	0.155301537	1.86238665
118	0.153387283	1.874789293
120	0.150155835	1.896081623
122	0.143100668	1.944206924
124	0.139843992	1.967227818
126	0.134282831	2.007807024
128	0.132876744	2.018333319
130	0.128381623	2.05274802
132	0.126293713	2.069145032
134	0.118225256	2.135163525

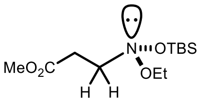

Fully decomposed in 4 h.

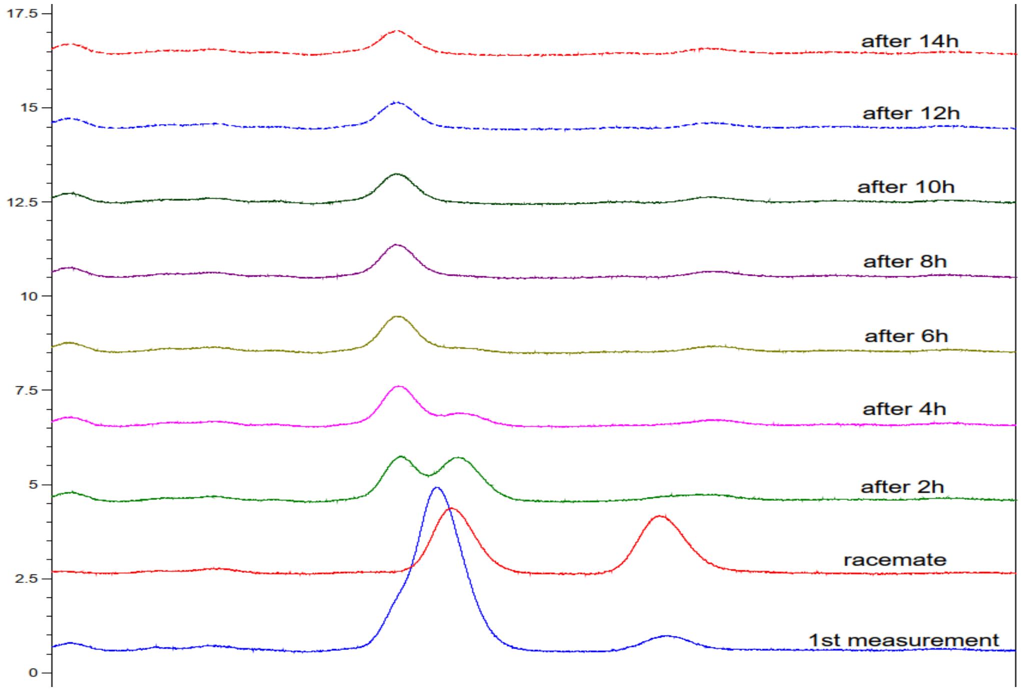

Supplementary Figure 2. Enantiomeric excess over time for **1e**

Supplementary Table 4. Enantiomeric excess over time for **1f**

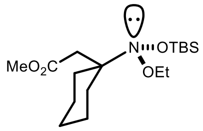

k_{rac} = 0.0119 h^{-1}, $t_{1/2}$ = 13.3 h, $\Delta G_{25\,°C}$ = 24.49 kcal/mol

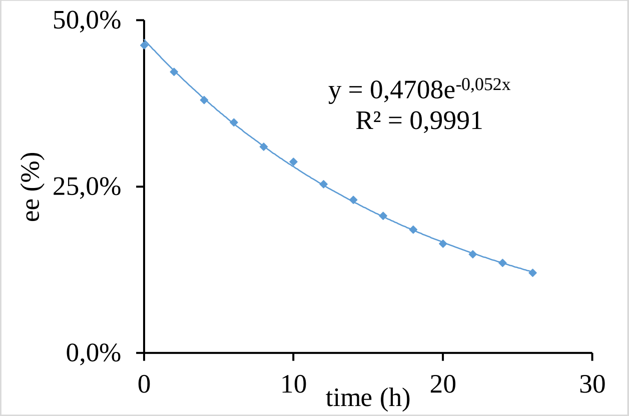

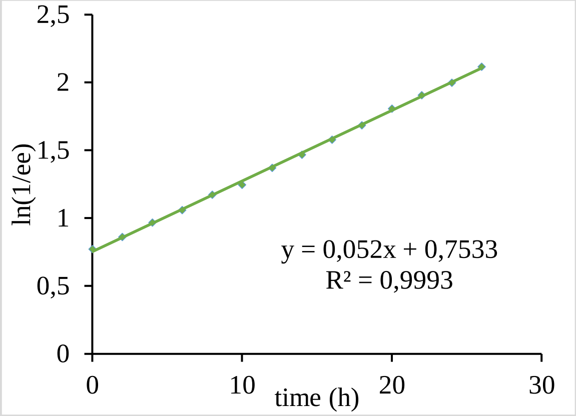

time	ee	ln(1/ee)
0	0.462158	0.771848455
2	0.42228	0.862086678
4	0.38016	0.967163062
6	0.34656	1.059699315
8	0.3097	1.172151192
10	0.28736	1.247019494
12	0.25362	1.371918195
14	0.23028	1.468459319
16	0.206	1.57987911
18	0.18528	1.685887085
20	0.16402	1.807766907
22	0.14854	1.906900997
24	0.135505283	1.998744649
26	0.12034	2.117434209

Supplementary Table 5. Enantiomeric excess over time for **1g**

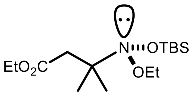

k_{rac} = 0.015 h^{-1}, $t_{1/2}$ = 46.2 h, $\Delta G_{25\,°C}$ = 25.23 kcal/mol

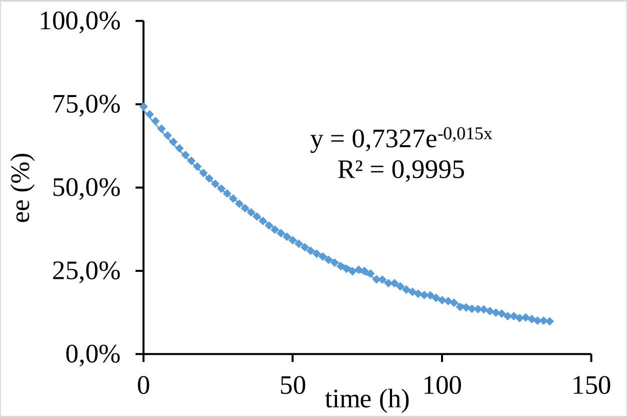

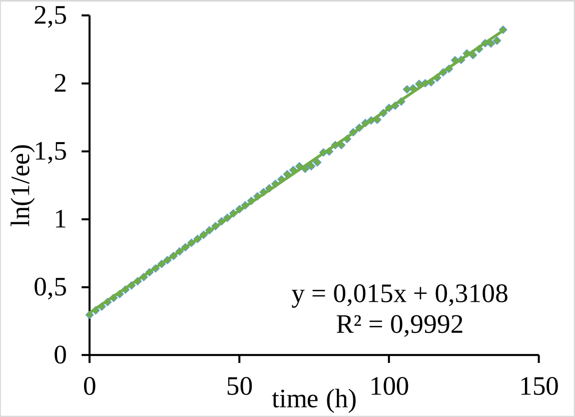

time	ee	ln(1/ee)
0	0.74292	0.297166912
2	0.71962	0.329031984
4	0.69936	0.357589648
6	0.67694	0.390172636
8	0.65672	0.420497531
10	0.63734	0.450452014
12	0.61792	0.48139628
14	0.59818	0.513863567
16	0.58028	0.544244533
18	0.56272	0.57497311
20	0.54396	0.608879564
22	0.52792	0.638810522
24	0.51072	0.671933784
26	0.4969	0.69936648
28	0.48226	0.729271891
30	0.46658	0.762325784
32	0.45194	0.794205851
34	0.4379	0.825764705
36	0.42558	0.854302335
38	0.41286	0.884646727
40	0.3994	0.917791858
42	0.38664	0.950261251
44	0.37394	0.983659922
46	0.36356	1.011810934
48	0.35212	1.043783252
50	0.34188	1.073295481
52	0.33172	1.103464039
54	0.32084	1.136812722
56	0.31044	1.169764633
58	0.3014	1.199316993

60	0.29286	1.2280606
62	0.28342	1.260825383
64	0.2745	1.292804018
66	0.26422	1.33097319
68	0.25606	1.362343487
70	0.24872	1.391427513
72	0.253596922	1.372009192
74	0.24922301	1.389407161
76	0.242058928	1.41857408
78	0.225075261	1.491320438
80	0.223167901	1.499830872
82	0.213201703	1.545516601
84	0.21307007	1.546134198
86	0.203560303	1.591792991
88	0.194127097	1.639242193
90	0.18748271	1.67406865
92	0.181270579	1.707764453
94	0.177718421	1.727554884
96	0.176711586	1.733236336
98	0.168518315	1.780710842
100	0.162133545	1.819334931
102	0.15959672	1.835105147
104	0.154689646	1.866334453
106	0.141490634	1.955521757
108	0.140319012	1.963836792
110	0.135817983	1.996439647
112	0.135251972	2.000615779
114	0.134204105	2.008393466
116	0.129825877	2.041561134
118	0.124633577	2.08237723
120	0.121523579	2.107646968
122	0.11406861	2.170955169
124	0.113857487	2.172807729
126	0.108473935	2.221245362
128	0.109846594	2.208670485
130	0.10493875	2.254378435
132	0.100726803	2.295343353
134	0.10086334	2.293988751
136	0.098707976	2.315589526
138	0.091245431	2.394202359

Supplementary Table 6. Enantiomeric excess over time for **1h**

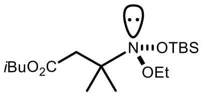

k_{rac} = 0.0143 h^{-1}, $t_{1/2}$ = 48.5 h, $\Delta G_{25\ °C}$ = 25.25 kcal/mol

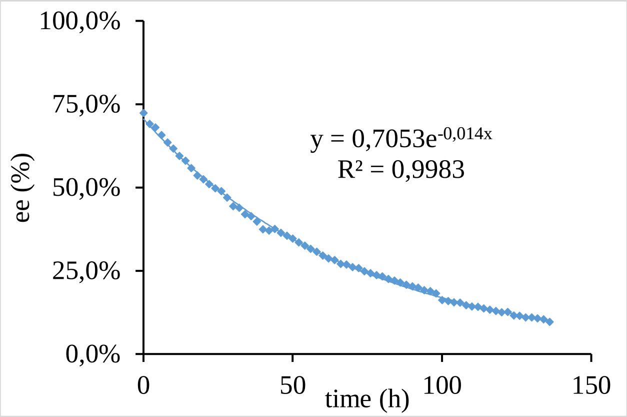

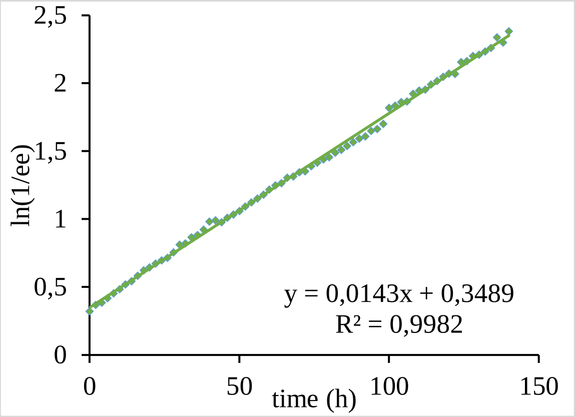

time	ee	ln(1/ee)
0	0.724	0.322963887
2	0.691009709	0.369601405
4	0.68028	0.385250801
6	0.65758	0.419188849
8	0.63518	0.453846856
10	0.6166	0.483534764
12	0.59484	0.519462817
14	0.58054	0.543796574
16	0.55828	0.58289465
18	0.53648	0.622725996
20	0.52478	0.644776152
22	0.51006	0.673226913
24	0.49766	0.697838166
26	0.48918	0.715024759
28	0.46962	0.755831422
30	0.44374	0.812516474
32	0.43912	0.822982555
34	0.42008	0.86731011
36	0.41364	0.882759249
38	0.39724	0.923214647
40	0.3746	0.981896489
42	0.37066	0.992470079
44	0.37582	0.978644974
46	0.36436	1.009612889
48	0.35538	1.034567639
50	0.34632	1.060392076
52	0.33526	1.092848929
54	0.32524	1.123191908
56	0.31566	1.153089594
58	0.30714	1.180451609

60	0.29596	1.217530969
62	0.28718	1.247646082
64	0.28202	1.265777289
66	0.2711	1.305267522
68	0.26848	1.314978856
70	0.26064	1.344615134
72	0.25864	1.352318146
74	0.24874	1.391347105
76	0.24278	1.415599596
78	0.23696	1.439863928
80	0.23286	1.457317864
82	0.22542	1.48978995
84	0.22094	1.509864108
86	0.21474	1.538327285
88	0.2086	1.567336736
90	0.2031	1.59405681
92	0.19982	1.610338318
94	0.19198	1.650364079
96	0.18922	1.66484492
98	0.18262	1.700347788
100	0.16212	1.819418477
102	0.15954	1.835460604
104	0.15536	1.862010274
106	0.15478	1.865750525
108	0.1463	1.922095971
110	0.14274	1.946730485
112	0.14166	1.954325459
114	0.136634099	1.990448736
116	0.13292	2.018007835
118	0.12888	2.04887354
120	0.12582	2.072902965
122	0.12616	2.070204336
124	0.11578	2.15606344
126	0.11482	2.164389594
128	0.11052	2.202558779
130	0.10972	2.209823613
132	0.10702	2.234739546
134	0.10426	2.2608675
136	0.09646	2.338626864
138	0.10028	2.299789006
140	0.09228	2.382927846

Supplementary Table 7. Enantiomeric excess over time for **1i**

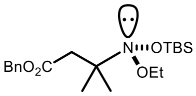

k_{rac} = 0.0188 h^{-1}, $t_{1/2}$ = 36.9 h, $\Delta G_{25\,°C}$ = 25.09 kcal/mol

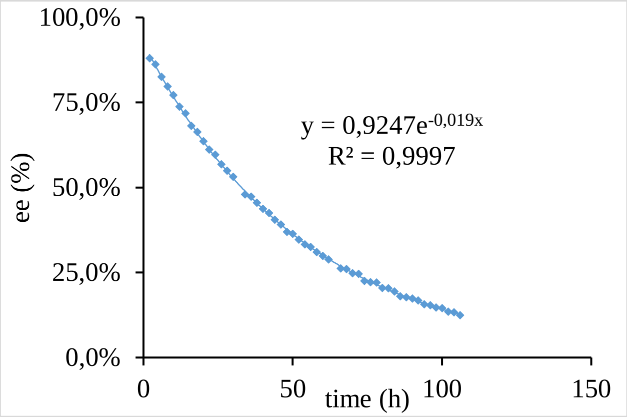

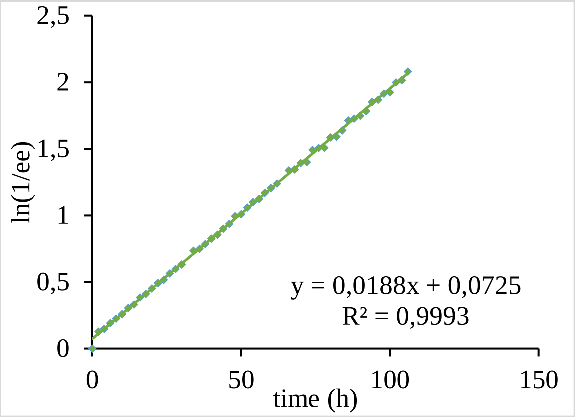

time	ee	ln(1/ee)
0	system not ready	
2	0.8808	0.126924694
4	0.862	0.148500008
6	0.8258	0.191402666
8	0.7974	0.226398844
10	0.7716	0.259288998
12	0.7378	0.304082494
14	0.718	0.33128571
16	0.6816	0.383312303
18	0.6634	0.410377152
20	0.636	0.452556716
22	0.6116	0.491676805
24	0.5968	0.51617323
26	0.569	0.563874845
28	0.5498	0.598200703
30	0.532	0.63111179
32	add solvent	
34	0.48	0.733969175
36	0.473	0.74865989
38	0.4556	0.786140047
40	0.4374	0.826907171
42	0.425	0.85566611
44	0.4058	0.901894852
46	0.3918	0.937003773
48	0.369513168	0.995568901
50	0.3644	1.009503114
52	0.3468	1.059007034
54	0.333	1.099612789
56	0.3252	1.123314901
58	0.3102	1.170538028

60	0.2994	1.205974807
62	0.2892	1.240636789
64	add solvent	
66	0.262140238	1.33887566
68	0.2606	1.344768614
70	0.248	1.394326533
72	0.2464	1.400799047
74	0.2252	1.490766383
76	0.2218	1.505979204
78	0.221	1.509592577
80	0.2048	1.585721386
82	0.204	1.589635285
84	0.1944	1.637837387
86	0.1804	1.712578671
88	0.1778	1.727095956
90	0.1738	1.749850066
92	0.1682	1.782601531
94	0.1568	1.852784171
96	0.1542	1.869504818
98	0.1474	1.914605299
100	0.1458	1.925519459
102	0.1354	1.999521919
104	0.1332	2.015903521
106	0.1248	2.081042823

Supplementary Table 8. Enantiomeric excess over time for **1j**

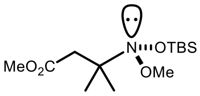

k_{rac} = 0.0119 h^{-1}, $t_{1/2}$ = 58.25 h, $\Delta G_{25\,°C}$ = 25.36 kcal/mol

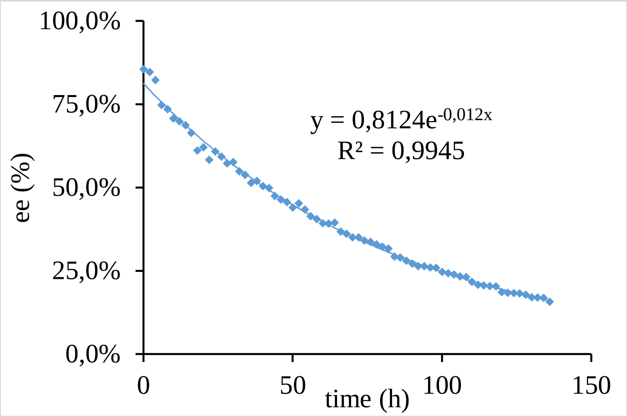

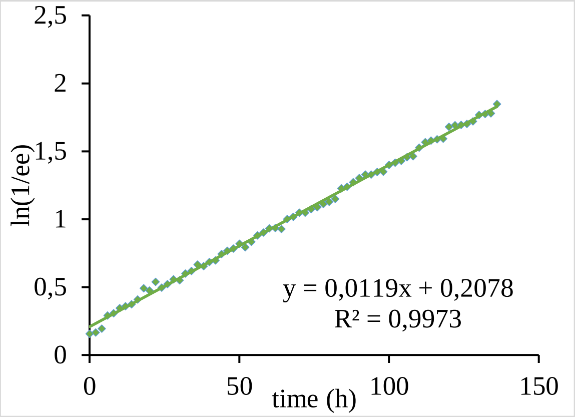

time	ee	ln(1/ee)
0	0.8553	0.156302994
2	0.84686	0.166219887
4	0.82258	0.195309537
6	0.74764	0.2908337
8	0.73532	0.3074495
10	0.70692	0.346837774
12	0.69832	0.359077829
14	0.68758	0.374577093
16	0.66434	0.408961212
18	0.61162	0.491644104
20	0.62096	0.476488611
22	0.5829	0.539739634
24	0.60832	0.49705422
26	0.59314	0.52232482
28	0.57228	0.558126897
30	0.57626	0.551196331
32	0.5487	0.600203435
34	0.53826	0.619413564
36	0.51412	0.665298578
38	0.52002	0.653888007
40	0.50438	0.684425327
42	0.49834	0.696472704
44	0.4749	0.744651023
46	0.46408	0.767698328
48	0.45632	0.784560961
50	0.44024	0.820435246
52	0.45214	0.793763413
54	0.43424	0.834157902
56	0.41406	0.881744388
58	0.40566	0.902239909

60	0.39306	0.933793007
62	0.3921	0.93623837
64	0.39518	0.928413922
66	0.36774	1.000379112
68	0.3611	1.018600351
70	0.3506	1.048109306
72	0.35088	1.047310994
74	0.34114	1.075462329
76	0.3368	1.088265997
78	0.3291	1.111393623
80	0.32318	1.129545835
82	0.317	1.148853505
84	0.29312	1.227173197
86	0.28962	1.23918556
88	0.27998	1.273037107
90	0.27176	1.302835955
92	0.26448	1.329989645
94	0.26446	1.330065268
96	0.25972	1.348151151
98	0.25918	1.350232478
100	0.24682	1.399095953
102	0.24262	1.416258846
104	0.23914	1.430706124
106	0.2328	1.457575563
108	0.2314	1.463607464
110	0.21726	1.526660486
112	0.20864	1.567145
114	0.20638	1.578036149
116	0.20434	1.587970006
118	0.20304	1.594352275
120	0.18616	1.68114876
122	0.1839	1.693363147
124	0.18358	1.695104739
126	0.18246	1.701224308
128	0.17878	1.721599279
130	0.1709	1.766676689
132	0.1695	1.774902352
134	0.16874	1.77939621
136	0.1576	1.847695102

Supplementary Table 9. Enantiomeric excess over time for **1k**

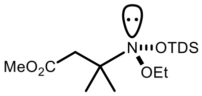

k_{rac} = 0.0168 h^{-1}, $t_{1/2}$ = 41.3 h, $\Delta G_{25\,°C}$ = 25.16 kcal/mol

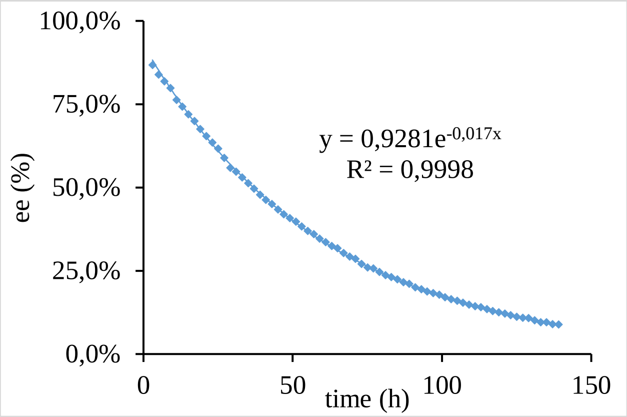

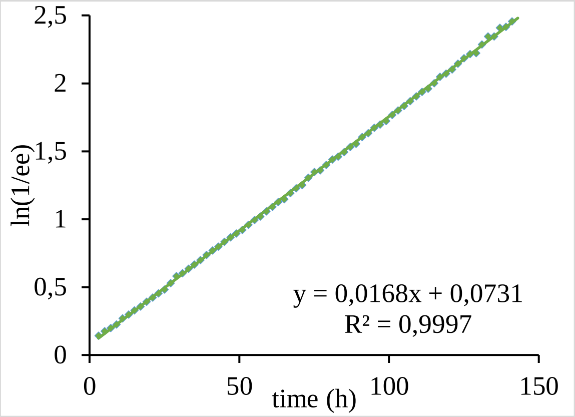

time	ee	ln(1/ee)
3	0.86770428	0.141904313
5	0.839273935	0.175218123
7	0.819020314	0.199646393
9	0.79887709	0.224548175
11	0.763266398	0.270148163
13	0.742791528	0.297339854
15	0.720197188	0.328230232
17	0.699646772	0.357179683
19	0.675340708	0.392537962
21	0.654119181	0.42446571
23	0.635356315	0.453569311
25	0.616791315	0.483224538
27	0.589152706	0.529069866
29	0.559238856	0.581178606
31	0.547362478	0.602644031
33	0.530098346	0.63469273
35	0.513403298	0.666693586
37	0.496882494	0.699401711
39	0.478161993	0.737805707
41	0.463306808	0.769365792
43	0.450523197	0.797345711
45	0.434538631	0.833470429
47	0.420201636	0.867020597
49	0.408697924	0.894778968
51	0.397671576	0.922128801
53	0.3834378	0.958577862
55	0.369848426	0.994662018
57	0.360501419	1.020259386
59	0.346800949	1.059004297
61	0.336194699	1.090064827

63	0.324484263	1.125518241
65	0.31776449	1.146444769
67	0.303443413	1.192560134
69	0.293211216	1.226862057
71	0.28618448	1.25111864
73	0.271081657	1.305335186
75	0.259986427	1.347125852
77	0.256877523	1.359155872
79	0.246573427	1.400095453
81	0.237127324	1.43915805
83	0.231705331	1.462288841
85	0.224533408	1.493730774
87	0.215911992	1.532884399
89	0.210931798	1.55622043
91	0.200949789	1.60470021
93	0.19518234	1.633821081
95	0.187719298	1.672807526
97	0.183245953	1.696926025
99	0.178730948	1.721873687
101	0.170644227	1.768174435
103	0.165041415	1.801558835
105	0.159748658	1.834153584
107	0.154287415	1.868938082
109	0.148746238	1.905513529
111	0.144083959	1.937359104
113	0.14069595	1.961154101
115	0.135311951	2.000172414
117	0.128949402	2.048335183
119	0.125915995	2.072140297
121	0.122165276	2.102380432
123	0.117056705	2.145096806
125	0.112336818	2.186253614
127	0.109023916	2.216188007
129	0.108398944	2.22193693
131	0.101506428	2.287633153
133	0.095855214	2.344916411
135	0.095865761	2.34480639
137	0.089803807	2.41012791
139	0.089246443	2.416353712
141	0.085766117	2.456131259
143	0.080588867	2.518394765

Supplementary Table 10. Enantiomeric excess over time for **1l** in MTBE

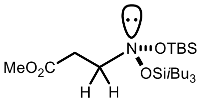

k_{rac} = 0.0027 h^{-1}, $t_{1/2}$ = 256.7 h, $\Delta G_{25\,°C}$ = 26.24 kcal/mol

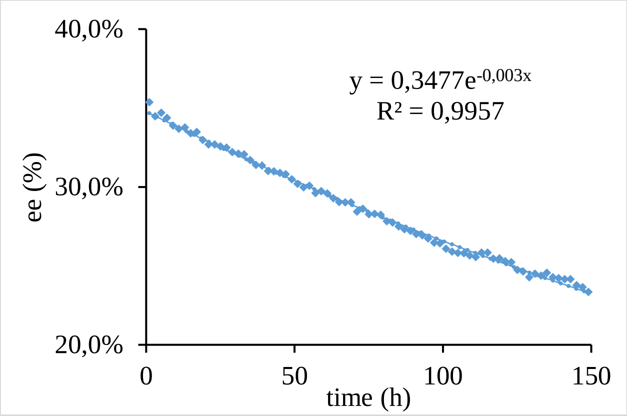

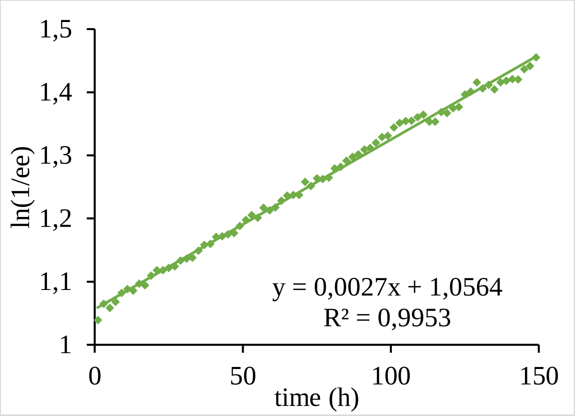

time	ee	ln(1/ee)
1	0.35374	1.0391931
3	0.34474	1.06496477
5	0.347	1.0584305
7	0.3437	1.0679861
9	0.3388	1.08234532
11	0.3368	1.088266
13	0.33764	1.08577504
15	0.33396	1.09673405
17	0.33482	1.0941622
19	0.32978	1.10932951
21	0.32694	1.11797861
23	0.32682	1.11834572
25	0.32578	1.12153297
27	0.32488	1.1242994
29	0.32204	1.13307952
31	0.32098	1.13637646
33	0.32056	1.13768581
35	0.31692	1.1491059
37	0.31402	1.1582986
39	0.31352	1.15989213
41	0.31008	1.17092495
43	0.30986	1.1716347
45	0.30882	1.1749967
47	0.30816	1.17713615
49	0.30482	1.18803384
51	0.3019	1.19765944
53	0.29966	1.20510678
55	0.3008	1.20130969
57	0.29614	1.21692296

59	0.29734	1.21287901
61	0.29592	1.21766613
63	0.29282	1.22819719
65	0.29048	1.23622055
67	0.29024	1.23704711
69	0.29014	1.23739171
71	0.28426	1.25786597
73	0.28608	1.25148379
75	0.28268	1.26343976
77	0.28292	1.26259111
79	0.28236	1.26457243
81	0.2782	1.279415
83	0.27754	1.28179021
85	0.27492	1.29127513
87	0.27318	1.29762436
89	0.27212	1.30151213
91	0.27006	1.30911112
93	0.26932	1.31185502
95	0.26724	1.31960815
97	0.26472	1.32908262
99	0.26426	1.33082181
101	0.26078	1.34407814
103	0.25896	1.35108167
105	0.25816	1.35417573
107	0.25798	1.35487322
109	0.25662	1.36015889
111	0.25554	1.36437633
113	0.25836	1.35340132
115	0.25834	1.35347873
117	0.25454	1.36829728
119	0.25484	1.36711938
121	0.25296	1.37452391
123	0.25238	1.37681939
125	0.2475	1.3963447
127	0.24638	1.40088022
129	0.24282	1.41543485
131	0.2451	1.40608899
133	0.24376	1.41157114
135	0.24556	1.40421396
137	0.2428	1.41551722
139	0.24222	1.41790887
141	0.2416	1.42047181
143	0.24162	1.42038903
145	0.23776	1.43649352
147	0.23654	1.44163795
149	0.23338	1.45508725

Supplementary Table 11. Enantiomeric excess over time for **1m** in MTBE

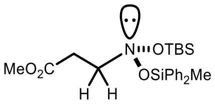

k_{rac} = 0.0024 h^{-1}, $t_{1/2}$ = 288.8 h, $\Delta G_{25\,°C}$ = 26.31 kcal/mol

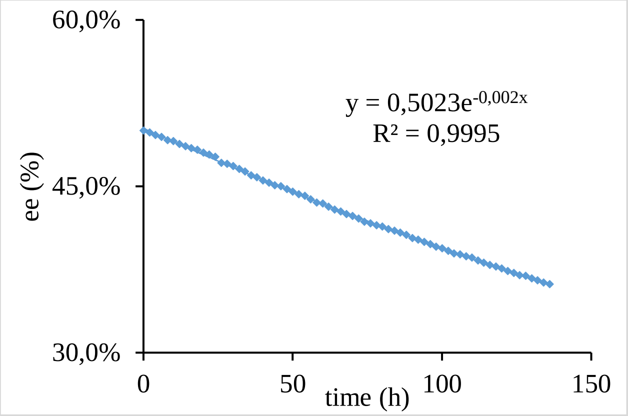

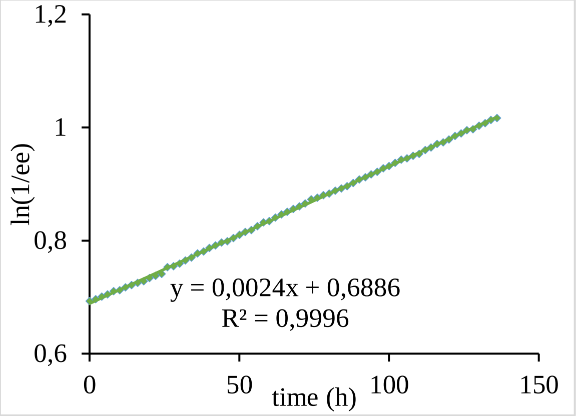

time	ee	ln(1/ee)
0	0.50012	0.692907
2	0.49858	0.695991
4	0.49616	0.700857
6	0.49432	0.704572
8	0.4916	0.71009
10	0.4907	0.711922
12	0.4882	0.71703
14	0.48616	0.721217
16	0.48442	0.724803
18	0.48286	0.728029
20	0.48016	0.733636
22	0.47842	0.737266
24	0.47664	0.740994
26	0.471	0.752897
28	0.4703	0.754384
30	0.46802	0.759244
32	0.46544	0.764772
34	0.46316	0.769683
36	0.45982	0.77692
38	0.45816	0.780537
40	0.4553	0.786799
42	0.45326	0.791289
44	0.45092	0.796465
46	0.44998	0.798552
48	0.44748	0.804123
50	0.445	0.809681
52	0.44272	0.814818
54	0.44126	0.818121
56	0.43806	0.825399

58	0.4352	0.83195
60	0.43428	0.834066
62	0.43144	0.840627
64	0.42906	0.846159
66	0.42708	0.850784
68	0.42486	0.855996
70	0.42314	0.860052
72	0.42096	0.865217
74	0.4179	0.872513
76	0.41658	0.875677
78	0.4148	0.879959
80	0.41354	0.883001
82	0.41136	0.888287
84	0.40988	0.891891
86	0.40816	0.896096
88	0.406	0.901402
90	0.40338	0.907876
92	0.40188	0.911602
94	0.39972	0.916991
96	0.39786	0.921655
98	0.3955	0.927604
100	0.39394	0.931557
102	0.39166	0.937361
104	0.38952	0.94284
106	0.38866	0.94505
108	0.38684	0.949744
110	0.3856	0.952955
112	0.383	0.95972
114	0.3811	0.964693
116	0.37892	0.97043
118	0.37768	0.973708
120	0.3758	0.978698
122	0.37358	0.984623
124	0.37182	0.989345
126	0.36974	0.994955
128	0.36914	0.996579
130	0.36678	1.002993
132	0.36508	1.007639
134	0.36308	1.013132
136	0.36176	1.016774

Determination of Absolute Configuration:

Method: The CD-spectra of **1c** (c = 3.0 x 10^{-3} M) were recorded in hexane (HPLC grade) at 20 °C and compared with the corresponding TD-DFT calculated. Measurements were performed on J-1100 CD spectrometer (JASCO) with a 2.0 mm Light Path QS High Precision Cell quartz cuvette (Hellma Analytics) at 20 °C (in sample holder equipped with a thermostat). The scans were made from 300 to 180 nm at a scan speed of 100 nm/min and 0.5 nm data pitch. The slit width was 1.0 nm. The High Tension Voltage never exceeded 700 V during measurements within the analyzed wavelength range. The solvent spectrum was subtracted from the final spectra. Spectra were recorded in at least three replicates.

CD Spectrum Calculation: Density Functional Theory (DFT) calculations were performed on the Max-Planck-Institut für Kohlenforschung computer cluster using the ORCA program package (Version 5.0-Stable).[9] The geometry of the input structure was pre-optimized using the XTB2 method (the semi empirical GFN2-xTB method) by Grimme et. al.[10] and possible conformers were generated using CREST (Conformer-Rotamer Ensemble Sampling Tool) based on GFN methods by P.Pracht, S.Grimme et. al.[11] resulting in more than 1000 conformer structures. All resulting conformers were optimized using the XTB2 method before subsequent calculations. To minimize the number of conformers the RMSD was used and replicates (RMSD < 0.5) were removed before subsequent calculation steps. Structural optimizations and frequency calculations were performed on 100 lowest energy conformers to identify all of the stationary points as minima (zero imaginary frequencies) and to obtain thermal and entropic corrections. Calculations were performed with the B3LYP functional[12] with D4 dispersion correction[13,14] and the def2-TZVP basis set[15] on all atoms. The libint2 library was used for the computation of 2-el integrals.[16] Strict SCF convergence and geometry optimization criteria were chosen.

The CD spectra were computed by time-dependent density functional theory (TD-DFT, NROOTS = 25) using ωB97x functional[17] at TZVP level of theory, solvent effects of hexane were taken into account using the conductor-like polarized continuum model (CPCM).[18] To exclude possible inconsistency between functionals chosen for the calculation, analogously (at the same level of theory, CPCM and NROOTS) spectra were calculated using m06-2x functional[18] and compared with the obtained using ωB97x. From the electron energies (calculated in the DFT step) and single-point energies (calculated in the TD-DFT step), the energies of all conformers were calculated and the Boltzmann distributions were plotted. All conformers with relative population less than 1% were omitted in following steps. Final CD spectrum was created using Multiwfn[20] with a 0.3 eV half-width at half-height. The final CD-Boltzmann-Distribution-Corrected spectra were generated and compared with the experimental ones (Supplementary Figure 1). The final CD spectra obtained with both used functionals at TD-DFT step were identical.

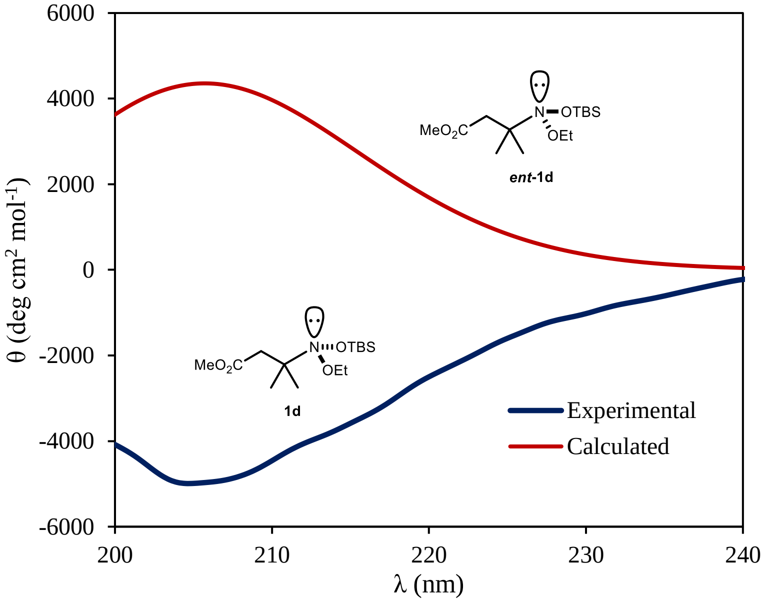

Supplementary Figure 1. Comparison between the calculated (Red, ωB97x/def2-TZVP level of theory) and experimental CD spectrum (Blue, recorded in hexane) of **1d**.

Cartesian coordinates for the representative conformer of *ent*-1d

C	2.56693293249697	-2.03229878091912	-3.98716841699430
O	1.96472945276816	-1.75036460770461	-2.71604336996983
H	1.82247991520859	-2.59273974460904	-4.54643701139991
H	3.47435645365672	-2.62183309021468	-3.85842906300828
H	2.81836977356896	-1.10667441006748	-4.50512952233023
C	2.72825265562205	-1.05305066543339	-1.84680377905425
O	3.85193595685950	-0.69623501758410	-2.10533949669688
C	1.99369629168067	-0.77648902268436	-0.56477841779694
C	1.52998817161278	0.69960174895285	-0.45845075133676
H	2.66509050379599	-0.99497594232897	0.26390760824529
H	1.12225921099929	-1.42180045270309	-0.49522223088364
C	2.70589713378243	1.63924189557070	-0.19517089912495
H	2.35154501732103	2.66659087563664	-0.09481901259226
H	3.23845750608816	1.35918773972235	0.71208881823091
H	3.40872189883891	1.58802171042386	-1.02504734843419
C	0.78504609352290	1.14700738968340	-1.71656380449304
H	1.48343677718579	1.21474700369918	-2.55148412471335
H	0.35076252747271	2.13472278802287	-1.56160706767854
H	-0.00664666611866	0.45213377711524	-1.98643213487924
N	0.58544237150448	0.85799555452302	0.69293721520842
O	-0.50183795045339	-0.03841510458471	0.46362635788174
O	1.25051761104851	0.34577845801164	1.83901985362023
C	1.19922455652396	1.28713566253009	2.91256984523561
C	1.91445522229862	0.65529845119204	4.08730732518718
H	0.15948932385579	1.50993778153750	3.16024156975810
H	1.68403979732079	2.21933350983641	2.60756103344736

H	2.95269832173740	0.43145183700878	3.83694503289642
H	1.42483953784052	-0.27334503571485	4.38434339816729
H	1.90584699513917	1.33844180418295	4.93873105117002
Si	-2.06491674908226	0.52918811176834	0.71883860343847
C	-3.11130987960461	-0.88123749661582	-0.00413404955176
C	-2.41967548972974	0.75948244162200	2.54550149489874
H	-3.49726547443426	0.80516666511020	2.72278254701028
H	-1.99275929670046	1.69110189767122	2.92115042480660
H	-2.01647395479249	-0.06384555250512	3.13779796632839
C	-2.34261527311282	2.15245311578088	-0.16902329834514
H	-1.60340984127352	2.88802081870138	0.15458644878060
H	-3.33305609187590	2.55223422099375	0.06200467370570
H	-2.26381902073698	2.05053970962724	-1.25231069714795
C	-2.89547340775075	-2.16335405494148	0.81813090021557
H	-3.21700019803269	-2.04051090585352	1.85497360334539
H	-1.84608982548701	-2.46387047368693	0.82589229393674
H	-3.47694107179240	-2.98810136367293	0.39116713144569
C	-4.59947167329592	-0.49480065921279	0.03798334153911
H	-5.21087969939323	-1.30767612113301	-0.36871958324121
H	-4.80716808530603	0.39761467320869	-0.55731497092339
H	-4.94760930719546	-0.30714569865656	1.05665941785984
C	-2.70184615835132	-1.14546053187974	-1.46302543150986
H	-1.65777808001057	-1.45210133863965	-1.53878330582757
H	-2.84074508032968	-0.26329675670838	-2.09294061135445
H	-3.31699964339073	-1.94722624587896	-1.88644666427178

DFT Coordinates Data

Method: Possible TS conformations were explored by the artificial force induced reaction (AFIR) method[21] implemented in the global route reaction mapping (GRRM) program.[22] An extensive conformational search was performed on possible catalyst-substrate orientations of reactant with AIMNet2[23] via Orca 5.0.3 program[24] using SC-AFIR for both enantiomers. The obtained approximately 1000 structures were sorted by energy. The energies of the lowest 30 structures were re-evaluated at r^2SCAN-3c level of theory. The lowest five geometries were then further optimized at PBE-D4/def2-SVP level of theory, and the most stable conformers were identified. Due to an overestimation of ΔΔG, another round of this procedure for $\mathbf{TS_{min}}$ was tried, however no significant decrease in the energy was observed. The reaction paths were subsequently generated from the corresponding conformers, and the transition state structures were optimized. All geometries were optimized using GRRM program at PBE-D4/def2-SVP level of theory with RIJCOSX and def2/J approximations. Thermal free energy corrections have been performed at the same level of theory using Orca 6.0.1 program[25] and the temperature was set at 193.15K. Transition state structures were verified by the presence of a single imaginary vibrational frequency and the corresponding intrinsic reaction coordinates (IRC). Solvation effect has been accounted by using SMD (toluene) solvation model as implemented in Orca 6.0.1 program. All single point energy is calculated at SMD(toluene)-ωB97X-V/def2-TZVPP level of theory.[26,27] IGMH analysis was performed using Multiwfn.[28,29] The wavefunction file was generated at ωB97XD/def2-TZVP level of theory using Gaussian 16.[30] The visualizations of the molecular geometries were generated using the ChimeraX[31] version 1.9 followed by rendering with Blender version 4.3. Conversion of enantiomeric ratio and ΔΔG was performed based on the Boltzmann distribution as follows: ΔΔG=RTln(pdt(*R*)/pdt(*S*)).[32] IGMH analysis[33] was conducted with Multiwfn 3.8(dev)[29,34] using the default parameters with isovalue of 0.004 a.u..

Results and Discussion

The non-covalent interactions between substrates and the chiral counteranion in **TS$_{maj}$** and **TS$_{min}$** were visualized by IGMH analysis (Supplementary Figure 2). As shown, while the substrates are well packed in the catalytic pocket in **TS$_{maj}$**, forming multiple noncovalent interactions, the silyl group of the nucleophile in **TS$_{min}$** is more isolated, resulting in a less efficiently constructed NCI network. In particular, relatively strong interactions between the silyl group of the nucleophile and one of the sulfonyl groups in the catalytically active site are observed in **TS$_{maj}$**, but are significantly weaker in **TS$_{min}$**.

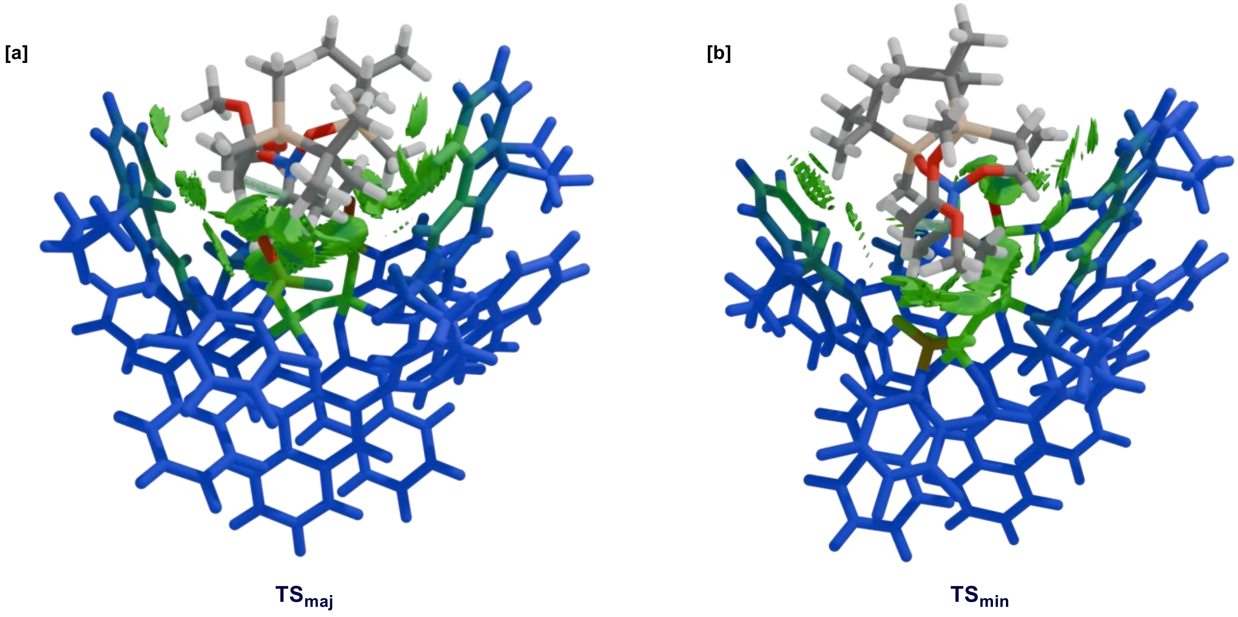

Supplementary Figure 2: [**a**] Visualization of multiple intramolecular noncovalent interactions within the catalyst anion, contributing to the stabilization of **TS$_{maj}$**. [**b**] Visualization of multiple intramolecular noncovalent interactions within the catalyst anion, contributing to the stabilization of **TS$_{min}$**. The color of the counteranion indicates the contribution of each atom to the depicted noncovalent interactions (increasing in strength in the order blue < green < red).

For a better understanding of the origin of enantioselectivity, distortion-interaction analysis was conducted following the Houk-Bickelhaupt protocol[35] (Supplementary Table 12). Gas-phase single point energies of the optimized **TS$_{maj}$** and **TS$_{min}$**, leading to the major enantiomer and minor enantiomer, respectively, were calculated at ωB97X-V/def2-TZVPP level of theory. Then they were decomposed into the catalyst fragment and the substrate fragment. The result suggests that the interactions between the catalyst and the substrates would be the major factor in controlling the enantioselectivity.

Supplementary Table 12: Summary of the distortion-interaction analysis of $\mathbf{TS_{maj}}$ and $\mathbf{TS_{min}}$

TS	ωB97X-V/def2-TZVPP (in hartree)	Relative energy ΔΔE (kcal/mol)
$\mathbf{TS_{maj}}$	-9589.859701	3.6
$\mathbf{TS_{min}}$	-9589.853902	
Substrate fragment		
subst- $\mathbf{TS_{maj}}$	-1724.326573	-1.4
subst- $\mathbf{TS_{min}}$	-1724.328732	
Catalyst fragment		
cat- $\mathbf{TS_{maj}}$	-7865.350979	-0.2
cat- $\mathbf{TS_{min}}$	-7865.351365	
Total distortion		-1.6
Total interaction		5.2 (major factor)

Supplementary Table 13: Energy table of the optimized structures. Energies are given in Hartree. Computed single point energies (E), Gibbs free energy corrections (Gcorr), Gibbs free energies (G), and the imaginary frequencies for transition states are provided.

Structures	E (solv)	Gcorr	G (solv)	Imaginary Frequency
$\mathbf{TS_{maj}}$	-9589.955402	2.2147951	-9587.740607	-133.94
$\mathbf{TS_{min}}$	-9589.949802	2.21498244	-9587.734819	-160.83

1. Cartesian coordinates of the optimized structures

TS_{maj}

O	0.202819449997	-3.456893015637	-1.333804491968
O	-2.081618981804	-2.216160424608	-1.317154682072
O	0.111353525753	1.261821571578	-0.391585414265
O	2.066288565744	-0.335186122488	0.088538088224
P	0.521941491736	-0.023697689985	0.561953466449
P	-0.846870356369	-2.669207372578	-0.325359436440
S	-0.113213458504	1.878515341847	2.620172108801
S	-0.748562655651	-5.170205711366	1.083153227495
O	-1.397852262536	2.245588865418	1.971852595559
O	-0.107299204267	1.843100653153	4.104654202994
O	0.732055489752	-5.135801113334	1.088141208896
O	-1.430003968157	-5.692382095631	2.292450277995
N	-1.429688835579	-3.757665572029	0.707868313960
N	0.519606831778	0.501433271064	2.083387257740
N	-0.291081451727	-1.330705285794	0.275886233353
C	-2.652692999374	-3.253847563781	-2.047823222488
C	0.417455573065	-3.024944925221	-2.633763722157
C	0.515858661840	1.314929649889	-1.717035999403
C	2.962901370829	0.724254172499	0.126121157504
C	-3.920054048033	-3.784461478285	-1.640812088140
C	3.979680639212	0.743999549345	1.139551581203
C	1.732216574676	-2.584040092330	-2.996584923131
C	-0.484313388525	1.196631338315	-2.734322581103
C	-2.229195057907	-6.622301444276	-5.557615243467
C	-1.699266269789	-5.528190157013	-4.888811105333
C	-2.437060963824	-4.871522149469	-3.861319395467
C	-3.726672300005	-5.400108169015	-3.497112260932
C	-4.252108653973	-6.512449963339	-4.218583779588
C	-3.524303563368	-7.106572458053	-5.236795752809
H	-1.636119403676	-7.122937583590	-6.337355651307
H	-0.691961357370	-5.167399170339	-5.137073462623
C	-1.932540133279	-3.732519581845	-3.142147771598
C	-4.433962172206	-4.829119356195	-2.406993554778
H	-5.239727322701	-6.905468566818	-3.932172788362
H	-5.423656545926	-5.238563026695	-2.154641974188
C	-0.033432481146	-1.476030869517	-7.448565709012
C	1.022685309902	-1.560466041409	-6.555132139267
C	0.829759831867	-2.070233052018	-5.237363527337
C	-0.473127022310	-2.547676037082	-4.854628115658
C	-1.542742504980	-2.435060029240	-5.791576003885
C	-1.329807085928	-1.902138011397	-7.054950486636
H	2.894615974099	-1.796769123071	-4.630779340104
H	2.028677940493	-1.218910228279	-6.839927247684
C	1.896588331761	-2.123480358947	-4.302889246655
C	-0.648273576535	-3.090458386549	-3.535929993215
H	-2.547977971099	-2.768863206384	-5.498977449230
H	-2.171321998753	-1.814800342834	-7.758802518212
C	4.051315838999	1.687842548200	-5.074518611406
C	3.680438153469	1.651481399947	-3.737966843324
C	2.308402803564	1.557113631870	-3.356495005603
C	1.319641186271	1.453039137575	-4.397751977240
C	1.730138209154	1.525663382606	-5.761744161103

C	3.068691346558	1.644898279296	-6.098280957746
H	5.116924164206	1.754808260338	-5.341054577903
H	4.453969351350	1.689195585128	-2.959724051292
C	1.870757861305	1.525713220749	-1.983796148390
C	-0.045722544912	1.279544387384	-4.054844938262
H	0.957385365390	1.467637805433	-6.542581005126
H	-0.784814758259	1.225678692815	-4.867779663483
C	5.510160044117	5.032731744225	-0.714324552777
C	5.695957408687	3.951626830143	0.131648894196
C	4.790442940947	2.849045291003	0.116735571449
C	3.704766661546	2.846500229983	-0.826450061031
C	3.517961748908	3.989738020088	-1.655621449755
C	4.397280334853	5.061365770973	-1.595745112408
H	5.764985020715	1.804475528008	1.732624002902
H	6.528198007085	3.939114075013	0.852192984153
H	2.655370996535	4.023213401708	-2.335229346758
H	4.225736782140	5.943508433277	-2.230314187202
C	4.910001377938	1.782950823780	1.042840955637
C	2.844360240284	1.696577339346	-0.872154268218
H	-3.934235527966	-7.973007984395	-5.776639852085
H	0.127757163656	-1.073965875175	-8.460399253736
H	3.373192971773	1.695832778456	-7.154701663027
H	6.203709218349	5.886064478208	-0.680636766342
C	-4.652240935641	-3.301832679952	-0.446864650807
C	-5.318063534725	-4.244611712793	0.375037125275
C	-4.713446969621	-1.931348518619	-0.109019635965
C	-5.400770219706	-1.502725846357	1.031206016644
C	-5.997325197162	-2.452393501740	1.872519240977
C	-5.975866712919	-3.828646232203	1.531455285000
C	-6.634728211914	-2.307773546954	3.182294471774
C	-7.006064160788	-3.597716951738	3.641349486682
C	-6.677837686934	-4.659266378270	2.602907068167
C	-6.863187413736	-1.171384201577	3.975287596594
C	-7.465984928391	-1.331632255335	5.233257361248
C	-7.841773044877	-2.609459516475	5.688109614451
C	-7.614934386633	-3.748359381378	4.891211114145
C	-5.826400079544	-5.863830458311	3.111714691041
C	-6.244985181579	-7.097800990748	2.264251766066
C	-7.440499736038	-6.642574168753	1.390801526389
C	-7.958379575505	-5.366671804024	2.064366959621
H	-5.243484075076	-5.309537807126	0.128444734920
H	-4.214876312335	-1.193194118062	-0.746491892933
H	-5.442533332840	-0.430914914173	1.267215942922
H	-6.575434277588	-0.171637144749	3.617034793871
H	-7.651602443968	-0.450440726586	5.866700897159
H	-7.914091740961	-4.744136679754	5.256096829430
H	-4.744307456504	-5.638678668660	3.055422221983
H	-6.058699113384	-6.034758496954	4.182648918395
H	-6.539485926390	-7.934778472554	2.929454006562
H	-5.411052501091	-7.474186503307	1.641675469351
H	-7.104490550887	-6.412231838474	0.359029538249
H	-8.227523529957	-7.417823331814	1.296886822513
H	-8.603622010542	-5.623846527889	2.932274733349
H	-8.550324095307	-4.708145336315	1.397743489319
H	-8.316671121927	-2.719744906621	6.675074263479

C	4.078750326372	-0.229674276124	2.254870036538
C	4.758787981852	0.177873025941	3.433610332354
C	3.538128896262	-1.539135013250	2.216821888253
C	3.680624044850	-2.416822292691	3.298763484261
C	4.378015477117	-2.002513317922	4.440817084564
C	4.918687270496	-0.695043725809	4.501811535762
C	4.605611794277	-2.655827039208	5.730758994175
C	5.269297746032	-1.736526538051	6.583589245325
C	5.592824687108	-0.446163673204	5.845542176209
C	4.250586863365	-3.933157787529	6.195266798022
C	4.542519221548	-4.276254666356	7.525020244910
C	5.183876060003	-3.358900684726	8.377259818298
C	5.557520689083	-2.085531144374	7.906260671304
C	5.120450929382	0.880176024363	6.547597346931
C	6.374100182298	1.766970813554	6.710970204446
C	7.336432017124	1.268065597052	5.624080686032
C	7.140049710630	-0.249567851877	5.676529585709
H	5.109852854280	1.214783302652	3.522530232162
H	3.011132517802	-1.889560863953	1.324541996388
H	3.231977606016	-3.417577506093	3.244112941327
H	3.739760864985	-4.648230759326	5.535173549724
H	4.260234765162	-5.270397427731	7.904635733855
H	6.068258241216	-1.376205711154	8.577516046794
H	4.366446790919	1.383547107898	5.910509950853
H	4.626888102543	0.665068940563	7.516049477157
H	6.834431187396	1.600920892594	7.708858236929
H	6.144725589226	2.849180813884	6.636777177403
H	7.033318651746	1.661486839432	4.629829898828
H	8.388837671373	1.576829411839	5.789235616467
H	7.652573052298	-0.657820661593	6.573477728685
H	7.525124235903	-0.798887559522	4.795112440390
H	5.398056076614	-3.639755112331	9.419796141042
C	2.891752134725	-2.618235745056	-2.068517744835
C	3.144490796399	-3.716248152377	-1.206692323468
C	3.813542919660	-1.545412131771	-2.098345437440
C	4.927795153238	-1.511450866262	-1.254338470323
C	5.155202130575	-2.591539541255	-0.388950270105
C	4.285699027279	-3.711904509189	-0.401235720988
C	6.170420760357	-2.802113376443	0.643293825917
C	5.964909121674	-4.084332074762	1.213384598761
C	4.811852813881	-4.804474946576	0.524118326219
C	7.165871750139	-1.945240920589	1.137195257494
C	7.962100118512	-2.380469679633	2.208412089902
C	7.763729938974	-3.653204145615	2.774184165857
C	6.764372641175	-4.511229397573	2.277771581622
C	5.319200287740	-6.041040409732	-0.318267007986
C	4.944302218711	-7.277333551892	0.507431734640
C	3.576422415645	-6.890153542943	1.079170007549
C	3.775399713257	-5.430888243191	1.517703868486
H	2.449893188519	-4.565194059891	-1.184735013389
H	3.619397148187	-0.696318198445	-2.765888503796
H	5.600367192787	-0.639990338187	-1.260249251488
H	7.305391300608	-0.941279482539	0.707210386343
H	8.744202002727	-1.719654023422	2.613324111383
H	6.609057305726	-5.497947596176	2.741654606661

H	4.766860086376	-6.060696136771	-1.280289706423
H	6.397496008431	-5.960390722210	-0.558765280302
H	5.675838304580	-7.428568878633	1.331794644689
H	4.934287684637	-8.208193881341	-0.096153366705
H	2.802548080157	-6.939338146026	0.284914781176
H	3.229423286070	-7.540272994969	1.908161475457
H	4.200601461993	-5.402274760230	2.541929787262
H	2.823670991106	-4.873234527090	1.534517807902
H	8.387765288995	-3.976812120417	3.621219760926
C	-1.930287507260	1.036581779184	-2.442567048262
C	-2.571175751018	1.751160800892	-1.397886830684
C	-2.706515583425	0.207427919002	-3.288655162117
C	-4.089738163954	0.088033396091	-3.123178696720
C	-4.717670003878	0.818018151045	-2.101700288033
C	-3.956372101974	1.654461587425	-1.244794928845
C	-6.117432564453	0.860486480441	-1.673761909237
C	-6.218028259299	1.756906273488	-0.581406010935
C	-4.866806748573	2.368164250806	-0.246835094885
C	-7.243805155726	0.157369619864	-2.130424345098
C	-8.473004026806	0.356091414658	-1.483322131324
C	-8.575058453015	1.246899108620	-0.398567704683
C	-7.446864356048	1.954647964260	0.055312786874
C	-4.842948585439	3.925197064974	-0.448143330791
C	-3.893238089256	4.453326522080	0.632640751306
C	-4.255052432962	3.593103193128	1.849085499813
C	-4.445605873879	2.183110575973	1.257467955062
H	-1.979096608877	2.383893836276	-0.723739324017
H	-2.203615891331	-0.376813934785	-4.072593929743
H	-4.668717723337	-0.582583465741	-3.776579142198
H	-7.163578207442	-0.548325187044	-2.971445325582
H	-9.364556889449	-0.193243981252	-1.822206635841
H	-7.534539923859	2.647523960859	0.907875653830
H	-4.563021496373	4.202983814378	-1.483374794260
H	-5.865031770460	4.319567900406	-0.266008750261
H	-4.007267402077	5.542595460519	0.809358599580
H	-2.834725917280	4.273774709989	0.353851441467
H	-3.474380156920	3.605427054612	2.634093882546
H	-5.204171950371	3.959887669700	2.298511979501
H	-5.204843717868	1.593844539192	1.809604143559
H	-3.492616940439	1.627009017366	1.308975239574
H	-9.545688357068	1.387708740839	0.101212092855
C	-1.235370510535	-6.306672047497	-0.266461300280
C	-2.530854114572	-6.853283105941	-0.256395877710
C	-0.370914399433	-6.688178806748	-1.309546942488
C	-2.961424859378	-7.749189281436	-1.242523597246
C	-0.789586022810	-7.590001286803	-2.303413024991
C	-2.078494450614	-8.140419096811	-2.258784939792
C	1.095479116295	3.167403548286	2.173955023308
C	2.310958348644	3.209973391695	2.881584855833
C	0.857968027871	4.145858146195	1.191505773010
C	3.251913549508	4.220958987243	2.653250996343
C	1.792366374537	5.171599876120	0.964404721592
C	2.976606903604	5.225829792345	1.713906588991
F	2.612955306335	2.279950474018	3.791222025591
F	4.418540833461	4.215248696154	3.309713270723

F	3.848503604240	6.212289143943	1.514298511943
F	1.560083378939	6.096168823314	0.029344645054
F	-0.240105094184	4.153757294880	0.433193686135
F	-3.409034723617	-6.540626843256	0.701659642035
F	-4.215526929206	-8.216680803484	-1.220843214529
F	-2.463465781064	-9.020295529788	-3.181062309704
F	0.043232392270	-7.935330473069	-3.288139579126
F	0.882617188139	-6.239266997176	-1.409575283564
C	-1.757780959725	-1.468319693658	3.919601774279
N	-1.892070597157	-0.792481018398	5.069714519521
O	-1.249427666407	-1.210864902482	6.191501740761
O	-3.117217793553	-0.228804014152	5.333069721300
Si	0.198121634091	-0.465041776734	6.900251176897
C	-0.184545451431	1.290746872176	7.448898802888
H	-1.071960736552	1.359373216327	8.108704470069
H	0.688209396627	1.685207121493	8.009899118177
H	-0.325193679906	1.935984216306	6.559279609421
C	1.626608847074	-0.475018699198	5.705910134276
H	2.514881278656	-0.074220333366	6.234175831477
H	1.884028676670	-1.486684524505	5.347207813566
H	1.426918420126	0.183560382556	4.840010280773
C	0.442270142568	-1.674833759223	8.358426678715
C	0.805672286088	-3.069122368505	7.812826102897
H	0.951252053637	-3.779250813070	8.658267470546
H	0.009075477040	-3.479370379592	7.161731733164
H	1.755466520686	-3.057969044839	7.240742945410
C	1.606457962845	-1.142993295163	9.219928752486
H	1.391978905863	-0.142484737208	9.650565115261
H	1.793384380220	-1.833932898490	10.071785785742
H	2.554967582948	-1.078502237901	8.646083401097
C	-0.845427972785	-1.766095377223	9.198218383043
H	-0.683923379820	-2.435415273074	10.072567484442
H	-1.159779933965	-0.777996965788	9.595582962459
H	-1.682517030840	-2.184813208086	8.604828205251
C	-3.078884242924	1.225462738243	5.443136169670
H	-2.351710285222	1.618017559454	4.704634025717
H	-2.726682929299	1.494742431555	6.460413030786
C	-4.488147388764	1.717207027115	5.195591980276
H	-5.206015652617	1.256418319714	5.903438283249
H	-4.525086501677	2.816275399373	5.329726561846
H	-4.814254990460	1.489039540661	4.162409538172
C	-2.611274742355	-1.041382239484	2.771830661255
H	-2.623059318951	-1.832168089042	2.003957604984
H	-3.640344803582	-0.808177559251	3.088020768270
H	-2.172946368355	-0.128089492593	2.316553197867
C	-0.480810715765	-2.192282232808	3.680847252296
H	0.287586083346	-1.435867610193	3.410049055179
H	-0.151444093781	-2.764881761730	4.563798920230
H	-0.601224635147	-2.863915111320	2.815884178078
C	-2.598952597847	-3.980220357946	5.484565253775
C	-3.125307675612	-3.488205177455	4.314013070373
H	-2.716051823471	-3.888369436308	3.378026290661
H	-4.093672150112	-2.976305355123	4.296409881520
O	-1.449604492243	-4.649674861511	5.513372658410
O	-3.117200617555	-3.814957030046	6.720690414343

C	-4.323989270948	-3.062962659229	6.847099277391
H	-4.590040384390	-3.088085513007	7.919383271141
H	-4.168226851653	-2.012736461682	6.523738988830
H	-5.145376746295	-3.510786084876	6.249443643743
Si	-1.210630371154	-6.335246693345	5.874558912130
C	-1.405498365440	-6.520417093305	7.739794528206
H	-2.422740149813	-6.197988175303	8.038608442805
H	-1.260267778748	-7.571689554210	8.062503154028
H	-0.677086819624	-5.887377872594	8.284087253838
C	-2.575081980848	-7.255446046595	4.967680005685
H	-3.568229005486	-6.932792788788	5.342066509458
H	-2.520935233634	-7.041309303064	3.882428451925
H	-2.492456148889	-8.350385102695	5.122473381680
C	0.576681396295	-6.727999517368	5.259911481294
C	0.510601751691	-7.863443843037	4.216228179531
H	-0.061661168907	-7.552179351373	3.320795971962
H	1.539099468873	-8.126190086874	3.880954904903
H	0.052360010669	-8.789202749165	4.623409329071
C	1.208790668705	-5.488543046220	4.600155374740
H	1.317665968718	-4.645725599798	5.310564925512
H	2.222673098467	-5.751231844443	4.222892746866
H	0.620799593482	-5.141344785336	3.732804254342
C	1.450625583181	-7.173400255438	6.450151634904
H	1.052937319382	-8.078841193528	6.954223672740
H	2.475879071901	-7.420992847674	6.094206024224
H	1.552532547933	-6.378172149978	7.217575130156

TS_{min}

O	0.012108979571	-3.493976980733	-1.324488092071
O	-2.047118388288	-1.993268942971	-1.691016439652
O	-0.182650948238	1.100554279027	-0.549602274486
O	1.737105343046	-0.409221136144	0.241848917338
P	0.152671926687	-0.077049777005	0.555081295307
P	-1.137557214702	-2.648532608249	-0.488763050475
S	-0.517078761606	2.009467969047	2.449761771245
S	-1.657576292422	-5.212804406420	0.753316190251
O	-1.472156636428	2.624111937469	1.499858052177
O	-0.923033735796	1.972112432684	3.876296082156
O	-0.235853828699	-5.363290873743	1.168682864273
O	-2.690497485059	-5.680194066848	1.710367326338
N	-2.032315299717	-3.722842114823	0.300641580604
N	-0.021692705654	0.537185223304	2.029685973851
N	-0.597671940086	-1.450890753066	0.377931070371
C	-2.654757377630	-2.969106083437	-2.478532410033
C	0.450028199092	-3.154619058516	-2.593233751505
C	0.309709718860	1.068960616026	-1.845101350544
C	2.625010215858	0.656413326717	0.222124681331
C	-4.005744755435	-3.327813468784	-2.181337394933
C	3.543162262750	0.830131109660	1.313469430381
C	1.848120903943	-2.882755704524	-2.754938192595
C	-0.639554834986	1.022175904117	-2.918592184225
C	-2.311796515503	-6.484015579924	-5.844586550766
C	-1.704975497769	-5.465841538967	-5.123850346291
C	-2.449465093288	-4.677270899581	-4.197636403407
C	-3.834155906182	-5.010784560338	-3.972372781284

C	-4.431329333948	-6.053155833269	-4.742097089990
C	-3.691626678262	-6.768313244805	-5.669557169245
H	-1.714427127426	-7.084974734091	-6.546193703814
H	-0.633499089938	-5.265653387491	-5.256911521276
C	-1.871161317337	-3.591561357233	-3.448785815036
C	-4.571017299310	-4.329267749062	-2.965498543892
H	-5.491459136362	-6.291810966162	-4.567618877070
H	-5.619631131451	-4.615465073788	-2.793014877019
C	1.012814331394	-2.112634224052	-7.534787203897
C	1.886489554062	-2.232377071399	-6.466082181507
C	1.408104355318	-2.527583446566	-5.155752389768
C	0.001040309821	-2.759686651988	-4.958951837059
C	-0.877559396256	-2.578413697394	-6.067550192118
C	-0.383808145014	-2.258736188196	-7.324586194754
H	3.362653463077	-2.426158460409	-4.216450343211
H	2.967675784078	-2.084508683251	-6.606361348188
C	2.289478380366	-2.596993397262	-4.045578622781
C	-0.461338382527	-3.158269111743	-3.656550615987
H	-1.959346938561	-2.700379899953	-5.917940597924
H	-1.080859512645	-2.125159900731	-8.165790335131
C	4.051799260610	0.969877374245	-4.993177192671
C	3.601439301638	1.044934078157	-3.682434602591
C	2.206824506435	1.067023715733	-3.383834790583
C	1.276061407865	0.966761799651	-4.476517422035
C	1.769182401997	0.915014787826	-5.812709118879
C	3.130028915702	0.924779726656	-6.071038169939
H	5.133415873165	0.952492452350	-5.195806446500
H	4.328620733838	1.094883339093	-2.861052441638
C	1.689896186031	1.180026852698	-2.044530275406
C	-0.116565312252	0.967047669821	-4.209657585921
H	1.042672766151	0.851169156207	-6.636380847556
H	-0.806191146527	0.968465713170	-5.066006457582
C	5.289782957105	4.802751150236	-1.004683015365
C	5.370009446771	3.868901849478	0.015106310065
C	4.456385282555	2.774300333944	0.076143702322
C	3.468577901276	2.623776268455	-0.957880172802
C	3.385576089683	3.624428180512	-1.968953953528
C	4.277763256421	4.685886091317	-1.993218993078
H	5.262787598659	1.990664061971	1.914611518528
H	6.123757706734	3.970791254900	0.811030150829
H	2.596311916391	3.554815219497	-2.729023244271
H	4.189712331528	5.453196040356	-2.776852756837
C	4.475524399409	1.859786335342	1.158768753319
C	2.600732180693	1.476945338440	-0.907897817506
H	-4.162621665993	-7.576244524377	-6.248872626563
H	1.395871983888	-1.883182379949	-8.540700846967
H	3.497077808738	0.880143151026	-7.107505694860
H	5.991764177702	5.649477098560	-1.035252007146
C	-4.751439322977	-2.709598665847	-1.058675051294
C	-5.491818335922	-3.538212531571	-0.183584671648
C	-4.737573865121	-1.313778835889	-0.836762106919
C	-5.507274065194	-0.734484722972	0.179056486956
C	-6.257216182865	-1.564301977522	1.025407897527
C	-6.209410579526	-2.970190429836	0.865972303198
C	-7.167217342673	-1.258982847526	2.132875073874

C	-7.630046878701	-2.485807128836	2.679145872788
C	-6.986818322511	-3.670214486793	1.969180803144
C	-7.627038801229	-0.030496575623	2.636351986350
C	-8.553907838328	-0.036129630696	3.692006636650
C	-9.010120027763	-1.250132198503	4.237259796319
C	-8.548734415326	-2.481109632588	3.732807971781
C	-6.009326820699	-4.475763572550	2.917118531119
C	-6.680915869671	-5.835932453355	3.148932600793
C	-7.366833324066	-6.110797942657	1.805370032589
C	-7.998852612428	-4.752347625454	1.462502971085
H	-5.455261153323	-4.627055790930	-0.310227955503
H	-4.137217916330	-0.671324387139	-1.487484285212
H	-5.519881691461	0.356304164030	0.298764476131
H	-7.287098723132	0.918499259454	2.193438181608
H	-8.933959465347	0.916729423396	4.090976504641
H	-8.916298600225	-3.426390311437	4.162844511962
H	-5.042241733727	-4.628333270491	2.397619056142
H	-5.799703530278	-3.919211659401	3.851267371152
H	-7.445268629812	-5.765796403195	3.954453357945
H	-5.954975251360	-6.618844318743	3.447988956000
H	-6.599722776694	-6.387144688161	1.050251176405
H	-8.107909623846	-6.935397244707	1.839343193312
H	-8.956804881103	-4.640130997267	2.011814865385
H	-8.228708735193	-4.622275146244	0.386636389017
H	-9.738186686722	-1.237290119866	5.062713221226
C	3.561704839273	0.000490195681	2.545607894392
C	4.208688446366	0.515097334037	3.702422459207
C	3.008954886568	-1.302761569749	2.623118644274
C	3.138271009876	-2.082502779526	3.778146510432
C	3.833876436619	-1.577554962552	4.882979365687
C	4.354305045868	-0.261783702500	4.846210742236
C	4.166665801423	-2.172470315783	6.176526911277
C	4.884100992658	-1.212698420914	6.935136672731
C	5.060284326059	0.081427915149	6.152939887710
C	3.908561896131	-3.450477310272	6.695370635092
C	4.363357162227	-3.760972813050	7.986760937356
C	5.065893797344	-2.806806749843	8.746069965582
C	5.331685625222	-1.527527255977	8.221166926605
C	4.458943145247	1.352363755619	6.868564071982
C	5.631148064588	2.326017297102	7.093196820901
C	6.612450086450	1.988332327597	5.964472276391
C	6.566129516453	0.456606091781	5.927103324340
H	4.586989191351	1.545385688759	3.707542868957
H	2.507966849873	-1.735003599947	1.752473634172
H	2.715601435603	-3.095553902561	3.794619690919
H	3.362022497050	-4.196073270380	6.098645643742
H	4.168231303239	-4.758397865292	8.409376050312
H	5.888321836450	-0.789577944133	8.820947860148
H	3.701201946024	1.814765335221	6.207615647977
H	3.945459719610	1.076141495609	7.809820097066
H	6.115783787457	2.128038685175	8.074018118349
H	5.308270718165	3.386505243547	7.096870043491
H	6.247258153244	2.417123586261	5.006354605720
H	7.634394692895	2.386098913268	6.130224517195
H	7.167786026715	0.049701511269	6.766928269929

H	6.961225214208	0.009193189116	4.994517286474
H	5.414419632808	-3.064352526956	9.757953992057
C	2.812582568124	-2.910765335301	-1.624583648307
C	2.801530702310	-3.932559126482	-0.642074557743
C	3.812855590591	-1.911020076606	-1.559554150904
C	4.759102032229	-1.883144428723	-0.528600992303
C	4.730495101243	-2.892942491743	0.445290931942
C	3.768955519250	-3.931060946831	0.363887764566
C	5.524398992900	-3.091210885583	1.659868567618
C	5.081772769718	-4.282061592568	2.291326556338
C	3.994669706312	-4.959775478574	1.465181375002
C	6.505287203647	-2.285220658166	2.257376885414
C	7.041876067395	-2.676793701669	3.494285508984
C	6.604813352447	-3.855946605291	4.123505451009
C	5.624174516011	-4.666137766773	3.521364752137
C	4.496300642969	-6.324422966374	0.855947546633
C	3.889915934913	-7.411808273765	1.748659094948
C	2.486492184416	-6.862822906530	2.027319860972
C	2.726579529207	-5.366623948776	2.296237686707
H	2.042917048528	-4.723599741411	-0.680449336290
H	3.813540196534	-1.116979592485	-2.318224828419
H	5.498393114641	-1.069382830733	-0.478449963828
H	6.828894560050	-1.348994359176	1.777425623929
H	7.806073461716	-2.052727439937	3.982186788239
H	5.275923583428	-5.577886594135	4.031410351716
H	4.086970760808	-6.423372405003	-0.170821050862
H	5.600580040881	-6.357679130532	0.776410631034
H	4.466940740610	-7.501040254251	2.695382293557
H	3.892417389918	-8.412260052161	1.269389460611
H	1.841090574097	-6.981405010418	1.132777963589
H	1.966681089389	-7.369316500904	2.865957538804
H	2.936542470297	-5.207395866856	3.374332802779
H	1.847606973378	-4.751099652496	2.031469141249
H	7.020381005619	-4.138389929738	5.102506677735
C	-2.105596817373	1.144715782437	-2.723933876516
C	-2.652926634440	1.981423112341	-1.716552615035
C	-2.982759293775	0.512096441432	-3.640784250187
C	-4.366189384954	0.714350867760	-3.587163728278
C	-4.894067047008	1.559300047102	-2.597063722835
C	-4.032407484371	2.193426022764	-1.666313714913
C	-6.276769458118	1.905941917818	-2.263094897344
C	-6.254779743329	2.785421389538	-1.151334705871
C	-4.829025962405	3.081515500483	-0.713603840423
C	-7.495041353915	1.483966673189	-2.819602833746
C	-8.693388204088	1.947465696964	-2.255150214899
C	-8.673731265699	2.820409494844	-1.151397766147
C	-7.452676190120	3.245753538557	-0.596004925484
C	-4.455526486367	4.599865684785	-0.887158736235
C	-3.535496603645	4.927535263924	0.294284876752
C	-4.180534862395	4.152831684969	1.449061630073
C	-4.559808296172	2.803915950843	0.811671322619
H	-1.993033148386	2.464981454303	-0.984438014083
H	-2.567087790862	-0.174332171598	-4.393550102601
H	-5.028697782867	0.192249942029	-4.294185537028
H	-7.511653513045	0.792382310990	-3.675897540530

H	-9.657504862432	1.621373886818	-2.674264778561
H	-7.446051492940	3.927924939546	0.269411650212
H	-4.014413440652	4.803142673827	-1.882879395273
H	-5.385267217624	5.202060309906	-0.810886053169
H	-3.453380497540	6.018111405500	0.481409884560
H	-2.509791928290	4.541538059131	0.120686756627
H	-3.502230888226	4.020864689304	2.314148917470
H	-5.089787702950	4.689850001763	1.799260696486
H	-5.439360310626	2.337540383985	1.297448939968
H	-3.714315658807	2.099187215523	0.916557520013
H	-9.622705648834	3.170403526801	-0.716975280484
C	-1.861812742439	-6.273161372924	-0.724865266675
C	-3.133559767051	-6.780826276474	-1.054833928280
C	-0.771110740203	-6.669443616431	-1.523384046015
C	-3.315614783400	-7.665592189334	-2.127368815022
C	-0.947658907556	-7.532407198904	-2.617162454616
C	-2.215955184922	-8.055096795009	-2.903428416344
C	0.974880815579	3.060848599285	2.424556334833
C	1.869130231750	3.035118682360	3.510932890879
C	1.245181080598	3.936540532842	1.355704987588
C	2.977028317610	3.892292334130	3.553537521009
C	2.331546789705	4.825781898748	1.411268216540
C	3.211021886670	4.789354206291	2.501627745063
F	1.703516720495	2.194800799616	4.537629791456
F	3.841478147972	3.837296380533	4.579470112707
F	4.278388914302	5.587220617997	2.529347442174
F	2.553928990668	5.674930911428	0.408550033312
F	0.494977122195	3.972592462943	0.254994212994
F	-4.228072237489	-6.453776145964	-0.360715258975
F	-4.534712897519	-8.132034147303	-2.410985401393
F	-2.374570233745	-8.905820349678	-3.915502698185
F	0.095998249620	-7.873168116765	-3.376879743644
F	0.480616857067	-6.269220829028	-1.286291237892
C	-1.770761797554	-2.175437421149	3.961146255787
N	-1.787360772913	-1.420590388791	5.070680596913
O	-2.968129149368	-1.134177269752	5.674426167282
O	-0.736444360456	-1.478358891145	5.955379248685
Si	-3.727153641723	0.463401738611	5.753080686176
C	-4.135998732917	1.064757302852	4.027614738202
H	-3.214517645943	1.190972006412	3.429794018421
H	-4.839942284178	0.392933574173	3.500128587467
H	-4.607815911473	2.066407225354	4.102235945004
C	-2.614375876535	1.657630315193	6.677788953667
H	-3.218524085869	2.523997512516	7.020171515178
H	-2.160003869938	1.186909794753	7.571894198242
H	-1.819458290364	2.044542772729	6.011011957634
C	-5.289513562324	-0.020775941002	6.744163661864
C	-4.935503818834	-0.233540902229	8.227425632907
H	-5.842981734886	-0.531021494944	8.798811790210
H	-4.182332101239	-1.034715741970	8.363364687105
H	-4.538032868509	0.688750369353	8.699733584593
C	-6.298298930480	1.138751885607	6.611662363886
H	-6.634312052057	1.278634763801	5.564523180551
H	-7.205038419723	0.925898481492	7.220513180283
H	-5.883114640465	2.103839743292	6.973842222543

C	-5.900615379786	-1.310712509078	6.163413850599
H	-6.863266477039	-1.540796353728	6.670512417792
H	-6.123707640972	-1.225014372323	5.080454330561
H	-5.225066843833	-2.177409354436	6.307849943712
C	0.246667167674	-0.409109256877	5.730558010136
H	-0.276439082414	0.470427674252	5.312400143037
H	0.981061697722	-0.754272936832	4.973645671232
C	0.920121980834	-0.115831918230	7.051259215389
H	1.460565462187	-0.999420583493	7.446345655710
H	1.665493819426	0.687233042033	6.896165306678
H	0.190473748185	0.226205370834	7.811071867408
C	-2.878392991028	-1.977058591879	2.974317316687
H	-2.657689819879	-1.049340097716	2.403490907026
H	-2.892228650577	-2.800896010385	2.240966648379
H	-3.864885338353	-1.873056424575	3.455042443679
C	-0.431024172663	-2.645158331942	3.496705025580
H	-0.543647934388	-3.384332233339	2.687234342891
H	0.123130893852	-1.777762003387	3.079728044915
H	0.160318115099	-3.069543343354	4.327951504686
C	-1.905438936214	-4.695562752094	5.741499443600
C	-2.720831534270	-4.250197691702	4.726090150334
H	-2.708449095800	-4.723455960009	3.735692365950
H	-3.607185654600	-3.686394398694	5.038876787189
O	-2.038276506167	-4.253699841514	6.983591579419
O	-0.883041076897	-5.566244062409	5.611234881283
C	-0.706780071268	-6.251890594720	4.364682556527
H	0.077620496128	-7.008666027272	4.545734366424
H	-1.646608135095	-6.744693529668	4.043565798369
H	-0.382240278221	-5.568419671385	3.557968671642
Si	-1.066307358671	-4.635793917703	8.379336637041
C	-1.309074695426	-6.456584436799	8.787385956087
H	-0.950750990405	-7.085309348968	7.948752753127
H	-0.737197787641	-6.735110146337	9.696312348314
H	-2.376580407536	-6.693742881770	8.967921860375
C	0.723858167068	-4.218029683964	7.999745898509
H	1.187567433683	-5.013007948920	7.384790307399
H	0.778127347276	-3.270138302658	7.429418298562
H	1.326797305378	-4.095015951601	8.922061833544
C	-1.837249462107	-3.481901942275	9.690769308515
C	-1.541557672422	-2.020813651576	9.301503380172
H	-1.911571093584	-1.783051835992	8.284663681067
H	-2.029740311081	-1.324414397504	10.019609012516
H	-0.454280612504	-1.802124902855	9.316514874486
C	-3.359900234077	-3.711360413976	9.742016213827
H	-3.615951099048	-4.750321404768	10.037706542516
H	-3.826778916995	-3.032478240475	10.489951440305
H	-3.836072572455	-3.510935052303	8.761448057374
C	-1.212378575944	-3.798772968737	11.063067294673
H	-0.109749074052	-3.665329344199	11.063370577802
H	-1.622131614399	-3.116090155200	11.840332660489
H	-1.428857437375	-4.836402405959	11.392920194388

Methods: All electronic structure calculations in this work were carried out with the ORCA program suite, version 6.0.[24,36] Since there are no crystal structures of the target compounds available, we have first constructed molecular models of **1a, 1b** and **1d** using the Scigress program suite (version 3.4; https://www.fujitsu.com/global/solutions/business-technology/tc/sol/scigress/) and minimized the energy of the structure using the MM3 force field.[37] Subsequently, we carried out a global optimization using the GOAT algorithm (https://www.faccts.de/docs/orca/6.0/manual/contents/typical/GOAT.html) as implemented in ORCA 6.0 together with GFN2-xTB semi-empirical Hamiltonian.[10] This optimization produced a set of conformers ordered by energy. For each target system around 20 of the lowest energy structures were geometry optimized using DFT with the PBE functional[38] and the D3BJ dispersion correction[13] as well as the def2-SVP basis set[14] and matching auxiliary basis sets for the resolution of the identity (RI) approximation.[39] Analytic frequencies were used to confirm the resulting structures as minima. The lowest energy structure of each target system after the DFT geometry optimization was then further optimized using the B3LYP functional,[40–42] D3BJ dispersion correction, the def2-TZVPP basis set[14] and the conductor like polarizable continuum (CPCM) solvent model[43] with THF as solvent. Analytic frequency calculations at the resulting minima demonstrated these structures to be minima and provided the zero-point and thermal corrections to the free energy. Next, the central nitrogen atoms were constrained to a planar geometry and reoptimized in order to provide a good guess for the transition state search that subsequently located the relevant inversion transition states at the B3LYP-D3BJ/def2-TZVPP CPCM(THF) level.

For studying the hypothetical model system, the nitronium and silyl-acene fragments were first geometry optimized separately using the PBE0 functional and the def2-SVP basis set. The inter-fragment C-C bond distance was then constrained to 3.0 Å and a relaxed surface scan perform while shortening the bond in steps of 0.1 Å down to 1.3 Å. In these calculations, the supermolecule was embedded in an ideal dielectric continuum using the CPCM model. In order to allow for electron-unpairing and possible single-electron transfer, the calculations were run in a spin-unrestricted fashion and each self-consistent field calculation was followed by a stability analysis. However, no unstable SCF solutions were found.

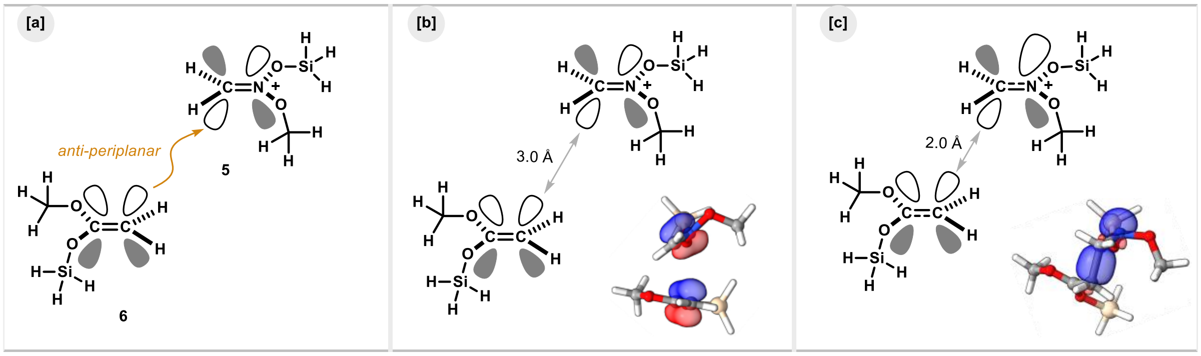

Supplementary Figure 3: Stereospecific Modeling of the Reaction. [a] Anti-periplanar trajectory of the SKA addition and the formation of the lone pair. **[b]** & **[c]** Computed optimized structures at selected points. The electronic structure features at selected points, long C–C bond formation coordinate showing the interaction of the HOMO of **6** with the LUMO of **5** as the key interaction leading to the breaking of the C=N -bond around 2.0 Å with the concomitant formation of the nitrogen lone pair around 1.8 Å.

Input string for GOAT calculations

```
! PAL4
! XTB GOAT #XTB version 6.4.0
* xyz 0 1
(initial guess structure)
```

Input string for geometry optimizations

```
! B3LYP D3BJ def2-TZVPP def2/J CPCM(THF) VeryTightSCF VeryTightOpt Freq PAL4
%freq  Temp  293.15, 298.15, 288.15, 303.15
       end
* xyzfile 0 1 1a-DFTopt01.021.xyz
```

Constrained optimization as guess for TS search

```
! BLYP def2-SV(P) def2/J TightSCF Opt CPCM(THF)


%geom Constraints
        { A 1 0 2   90.0   C}
        { A 1 0 3   90.0   C}
        { A 1 0 4   90.0   C}
        end
      end

* xyz 0 1
(optimized structure)
```

Transition state input

```
! B3LYP D3BJ def2-TZVPP def2/J CPCM(THF) VeryTightSCF OptTS Freq PAL4
%maxcore 8000

%geom  Calc_Hess    true
       Recalc_Hess  5
       TS_Active_Atoms  { 1 2 3 4 } end
       Trust        0.3
       end

%freq  Temp  293.15, 298.15, 288.15, 303.15
       end
* xyz 0 1
(Structure from TS guess)
```

Surface scan input

```
! UKS PBE0 D3BJ def2-SVP def2/J CPCM TightSCF Opt PAL8

%geom  scan
          B 0 1  = 3.0, 1.30, 180
            end
        maxiter 500
          end
%loc    locmet pm
```

end
* xyz 1 1
#Coordinates from ORCA-job attack2-01 E -1133.067437029121

C	-0.50411743860821	0.61440636613500	1.00421744269379
C	2.14939854907617	-0.57876484556763	1.73578775252454
C	0.26259077928686	1.49293012065680	0.32288369730842
O	0.27648815352797	1.66375966170248	-0.99775130330069
O	1.13230971782479	2.27635754274728	0.97736681071339
C	1.93679719564240	3.18577520747470	0.24055077711259
Si	-0.46926891435002	0.67845600777877	-2.16497824416247
H	-0.02393167518013	-0.72638466636303	-1.96429660892838
H	-1.94778362190604	0.78676572336442	-2.02589907114617
H	0.00056383791482	1.23741606668638	-3.45618875036126
H	2.58137036278295	2.65783054424414	-0.47669743629191
H	1.31739463286777	3.91763135420952	-0.29763352875255
H	2.55609125954016	3.70201771674331	0.98323418081413
H	-0.47057732602192	0.59967281783566	2.09343108042035
H	-1.21129695665626	-0.03431701198293	0.48660262622791
N	2.51355667189754	-1.07689337452139	0.61733034818955
O	2.69276748112358	-2.39058866379373	0.44918662636775
C	1.65582954024385	-2.99802476956455	-0.35433791942797
O	2.69914760205629	-0.32563630368981	-0.45481764881676
Si	4.16376011492899	-0.50512535390645	-1.41491448902886
H	0.68128659269738	-2.83643290455377	0.12622626955634
H	1.66848369303296	-2.59211146201893	-1.37439075676491
H	1.91650940484482	-4.06119939760719	-0.36271130400983
H	5.28832845540092	-0.55546673871579	-0.44833932208440
H	4.03365152584186	-1.74418727295221	-2.21868384043650
H	4.09863519837225	0.73193776995105	-2.22295037889305
H	2.13378693783660	0.51046835697458	1.82689436278471
H	1.92520802798144	-1.27676149126668	2.54409762769225

Optimized Coordinates:

1a-minimum in THF

33
Coordinates from ORCA-job 1a-DFTmin-thf-20deg E -709.894216279342

N	0.36121314037387	1.26940630876697	0.44748354124753
O	0.28187526633164	2.30393342860635	1.40954139158506
O	1.65759830042668	1.40888594374534	-0.15248776235663
C	0.33893214262792	-0.02076070791943	1.20297341403958
C	1.52360303733565	1.59106700991356	-1.56122962763320
C	-0.36749429648278	3.46404657907468	0.86392097398528
C	-1.84818049793777	3.26013572039566	0.61186506624735
C	1.45718888536709	-0.02891031999795	2.28104002751137
C	-1.05016886639997	-0.14228009640412	1.82883700832065
C	0.53917756454878	-1.13705538635408	0.17727308843305
H	1.05284988704577	0.72599995745238	-2.02998803250576
H	0.94987535957758	2.48883581917812	-1.79381807546406
H	2.54008075273439	1.69851103686562	-1.93710272729102
H	-0.20282685915317	4.22702249925737	1.62493652546218
H	0.15394117853082	3.77726735220315	-0.04217727359414

H	-2.35854328896181	2.96536729207377	1.52921782895810
H	-2.28980207165347	4.19261806248235	0.25566104985962
H	-2.01556238620016	2.49223496574344	-0.14194475663777
C	1.59708328083683	-1.35860049039755	2.96795034022730
O	0.89193426991410	-1.75643363417583	3.86996785746485
O	2.60681744879863	-2.08335847998093	2.45925186567020
C	2.79769228737824	-3.39780845302914	3.01649483114869
H	1.21937903943727	0.71283978476480	3.03956818693529
H	2.39915614427526	0.23218866398906	1.80660978911260
H	-1.22142500811566	0.63427658852059	2.57008104009024
H	-1.14780642632742	-1.11109984288480	2.31442666431648
H	-1.81352178899697	-0.06371971555623	1.05488429931194
H	0.38725617678020	-2.10485743407040	0.65357454181748
H	-0.19050791195079	-1.03844540003252	-0.62604937941467
H	1.53952374817695	-1.11782092990481	-0.24885037208524
H	3.64205548378958	-3.82074496955887	2.48191613656198
H	1.90661658704068	-4.00399959462554	2.86403059258602
H	3.01459720748129	-3.32942863852584	4.08068293625410

1a-transition state in THF

33

Coordinates from ORCA-job 1a-ts-thf-20deg E -709.853307345519

N	0.78488900947118	0.37024424292385	-0.38567604709679
O	1.78017428500479	-0.10551777171339	0.45589547190786
O	0.34500496301939	1.64726777787302	-0.11574401537463
C	0.11742559037221	-0.43145640214336	-1.41664100621731
C	-0.74724459168266	1.77667982235304	0.83339616988967
C	1.39644734572988	-0.42537320257310	1.81964103448913
C	0.52308430195797	-1.64996597722693	1.98665242444253
C	0.27278566277450	0.40383508284691	-2.73146759090129
C	0.85790555456554	-1.75976797090627	-1.55738299948154
C	-1.37435347460522	-0.68962165568451	-1.14462276642611
H	-1.15589865322661	0.80696938765128	1.09701844906715
H	-0.37730027488446	2.27899014317670	1.72734957684094
H	-1.52022675919414	2.38405047320709	0.36269733354089
H	2.36469358098809	-0.58133292140625	2.29562070088569
H	0.94249157353156	0.45284879199197	2.27723813165180
H	1.00225687896959	-2.53691322413780	1.57306214477933
H	0.35501481395996	-1.81938839568167	3.05204765309382
H	-0.45008767914624	-1.52497016824683	1.51610789751800
C	-0.46354872729709	-0.16244511879199	-3.91469904803468
O	-0.07712103398690	-1.08422311390683	-4.60085558606440
O	-1.62917072920662	0.46814921150498	-4.13296012339966
C	-2.43860192180757	-0.03395669897923	-5.21334778975232
H	1.33190402177193	0.43994028411026	-2.97956705002780
H	-0.08272658971143	1.41167732055187	-2.53567399926841
H	1.92043656270279	-1.60002406251807	-1.72978687760862
H	0.44580727669834	-2.30063322699145	-2.40639021753216
H	0.73597563108834	-2.37115951661604	-0.66771971060242

H -1.80069529734860 -1.29296778565150 -1.94704732420106
H -1.50865198105905 -1.23586168586868 -0.21359691709588
H -1.93759636475082 0.24026889346093 -1.09419858767252
H -3.32634508059838 0.59000901639196 -5.22680384573937
H -2.70509415195339 -1.07362329508678 -5.03169597170539
H -1.90159055585639 0.04508521522254 -6.15655753948146

1b-minimum in THF

30
Coordinates from ORCA-job 1b-DFTmin-thf-20deg E -670.585183742249
N -1.78909322444983 2.38660848853651 -0.08319978701482
O -2.98377547678310 2.88058858111145 -0.67030099975847
O -0.96285248905891 3.53036855933829 0.15520310931001
C -2.21267219228460 1.86343335072293 1.25046295067971
C 0.29149552420743 3.36175247832859 -0.50748816036373
C -2.87647451960517 2.91911073649455 -2.10326883996703
C -2.84101507439705 1.54669997477615 -2.74459217882588
C -0.97084612558320 1.29262143878836 1.94369371123951
C -2.88999479258265 2.91327099259658 2.12879393268791
H 0.15888690329691 3.24189166800338 -1.58299131177664
H 0.84950175066850 4.27449700239325 -0.30463247694627
H 0.83605350770884 2.50512587669889 -0.10882277094417
H -3.76695603985705 3.46968063906251 -2.40649803665442
H -2.00553005740285 3.51508993453916 -2.38104735677013
H -1.96387831586325 0.98672322030287 -2.42616091235897
H -3.73185945462660 0.97470072791951 -2.48355770290711
H -2.80716590272281 1.65509386988710 -3.83023046149991
C -3.18742727635174 0.71296839343130 0.95489375799488
O -4.29002693404647 0.61172575397907 1.43857525550392
O -2.65560127186650 -0.19465101441673 0.13446892927763
C -3.49089977584356 -1.31452924661170 -0.21508843302430
H -0.29885189870208 2.10333944278964 2.21811780732759
H -0.44617860636582 0.59502684132136 1.29479711061475
H -1.26921995588750 0.77165843570767 2.85310249530599
H -3.16241671153641 2.46662235078830 3.08307900124984
H -2.19770861058313 3.73147760786005 2.31180187436842
H -3.79046241079601 3.30336139013179 1.66269173995669
H -3.75006643512784 -1.88409361514067 0.67514103824619
H -2.89804635871318 -1.91682121436488 -0.89551187379186
H -4.39784975547702 -0.96582560439864 -0.70487440770190

1b-transition state in THF

30
Coordinates from ORCA-job 1b-ts-thf-20deg E -670.547365926929
N -0.03043572955237 0.89757559173206 -0.63446596918467
O 1.26057580163949 1.34556604196361 -0.38394064976617
O -0.99357726308628 1.39491745462357 0.22037718010128

C	-0.28170160728267	-0.11987612765395	-1.65103699766813
C	-1.36011958136784	0.56647697809536	1.34836046525164
C	1.89989471778636	0.91825494315979	0.84383669934034
C	2.16511787218755	-0.56659563652187	0.96499671592644
C	-1.75858276029821	-0.03720159228167	-2.05816653567179
C	0.59730456665826	0.19282635992431	-2.86673076221060
H	-0.48830781662908	0.16732181302854	1.85862065030773
H	-1.90312996232690	1.23574469113796	2.01326067079015
H	-2.00364641930347	-0.25069555930430	1.03185170845853
H	2.83877129523104	1.47071016683079	0.80803065150342
H	1.32781596106224	1.29654550011280	1.69221437073230
H	1.24932978525989	-1.13715589059716	1.10457191371235
H	2.68784605406949	-0.94517024970105	0.08788838242854
H	2.79213073202649	-0.74021496250575	1.84151528859279
C	0.02396011419930	-1.57202508605014	-1.19704833215666
O	0.89805359718678	-2.26920694379159	-1.65870057418350
O	-0.79998731572873	-1.98693617839394	-0.22871536397658
C	-0.59653091918846	-3.32153211345792	0.27183046877587
H	-1.96844751064729	0.95711671164828	-2.44828911185647
H	-2.42525208738284	-0.24019376806384	-1.22582126406625
H	-1.94987868051912	-0.77117280193012	-2.84071342501021
H	0.39107379935677	-0.52049744790917	-3.66284979504088
H	0.36750865751525	1.19744384852674	-3.21675390783615
H	1.65304132628488	0.13570515174323	-2.61896979356935
H	-0.73966780241510	-4.04737979971896	-0.52620634727275
H	-1.34125933483819	-3.45637837977356	1.04947980218471
H	0.40659391460589	-3.42271022695763	0.68145414198237

1d-minimum in THF

51

Coordinates from ORCA-job 1c-DFTmin-thf-20deg E -1197.185509660523

N	-1.10441434481117	2.60637104789544	1.38264827614401
O	-0.40974473131443	1.64202259368641	2.14951113116904
O	-0.77042273091127	2.31812732229220	0.01380589478385
C	-2.55299824430715	2.27718979918010	1.56622780523601
Si	-0.02031645226211	3.52021054619849	-0.90548563299831
C	0.84825443127972	2.13262888363821	2.63209648749928
C	0.72608374508170	3.31772600714158	3.56882135863476
C	-2.88267199001355	2.50282398663709	3.04210395641768
C	-3.35891639138381	3.26534765309714	0.72189913975342
C	-0.95356210168195	5.13974504934237	-0.84665715692418
C	1.73519856815046	3.84750148710662	-0.34426283660951
C	-0.05711108100048	2.74334768593639	-2.63557450876575
H	1.26583119029690	1.26870747175920	3.14920781015926
H	1.49571772868633	2.35992073546170	1.78598975439339
H	0.08804508119199	3.07733304365822	4.41964714000533
H	1.71578928281879	3.58454464523545	3.94384055410501
H	0.31082655669783	4.18323296399654	3.05525357138060

C	-2.25745181016971	-0.30316916181369	1.80025687690260
O	-2.47719641450567	-0.63015909859992	2.94628388723270
O	-1.43269570807514	-0.98893599588852	0.99200687709661
C	-0.73352126725232	-2.09636083394493	1.58523060723070
H	-3.95571161480360	2.38244803064390	3.18938103384706
H	-2.60779464901268	3.51803603542051	3.32875629683010
H	-2.36563693136389	1.80096549568352	3.68729726205320
H	-3.10412523302213	4.28904687175054	0.99059400860676
H	-3.17846640761615	3.12545091005898	-0.34073673433186
H	-4.42043000764883	3.11299110371143	0.91395972431047
H	-1.43782227757327	-2.83542588243434	1.96255053316686
H	-0.10126937853381	-1.74715928143798	2.40007667617474
H	-0.12785367073277	-2.51935543047532	0.79007030012389
C	-2.91175905229254	0.84851713438730	1.08265283176874
H	-3.98657368634577	0.73282892651663	1.22694745733545
H	-2.69892568504625	0.77707486770738	0.02022019919344
H	-1.95149427180588	5.06260343037796	-1.27635329889693
H	-0.39804008493991	5.89540418336870	-1.40607297380115
H	-1.05085076072536	5.48987783057013	0.18191594060238
H	2.21802412848082	4.52481515486294	-1.05231927155447
H	1.75125219320319	4.32989400992173	0.63416618987725
H	2.33097690696397	2.93604562539896	-0.28965285133885
C	0.75773403672308	1.43949096286615	-2.64360554587881
C	0.55047074281945	3.72758303177470	-3.64916927482947
C	-1.50713058971620	2.43285589672366	-3.04207388926677
H	0.72833644786754	0.98572382306280	-3.63967398256528
H	0.35891229984910	0.71325614025876	-1.93373343876842
H	1.80541324766435	1.61552521129689	-2.39277351731979
H	1.58802649700257	3.96844912828896	-3.41038899493028
H	0.53663421772006	3.28684140342311	-4.65115519666303
H	-0.01139308220902	4.66251458082027	-3.69241261488821
H	-2.12381457042281	3.33337030309485	-3.06733641394615
H	-1.97283857614270	1.72559403914730	-2.35499790646888
H	-1.52682230473224	1.99096164255444	-4.04362677325371

1d-transition state in THF

51

Coordinates from ORCA-job 1c-ts-thf-20deg E -1197.152477122277

N	-0.09081856844682	-0.11086624029140	0.16695191880639
O	-1.15237856152976	-0.77890154790865	0.72059264914584
O	-0.31891467981459	1.24393561538920	-0.10456418650629
C	1.23011993273058	-0.67300292942021	-0.02684182395437
Si	-0.86145806757122	2.25076497304228	1.14916040642562
C	-2.09365996809113	-1.22966784339521	-0.28690092660980
C	-3.22984572086866	-1.91646397696591	0.43400442936253
C	2.30367206143758	0.22769239596224	0.59669899341842
C	1.27092382618960	-2.04796581212213	0.64210037234141
C	-2.71732167124218	2.07360334574615	1.32287892202288

C	-0.03094666309293	1.82035194339536	2.76698251658709
C	-0.40652431545971	3.98913004267424	0.54193705429274
H	-2.43931027158116	-0.36133805614267	-0.84982216526670
H	-1.57366663549045	-1.90694024113602	-0.96678867540855
H	-3.73147347278209	-1.22759758292202	1.11430657994537
H	-3.96194712397872	-2.27631492547153	-0.28978652624730
H	-2.86703613991924	-2.77019583260961	1.00783420238024
C	1.36417511173841	0.35564394691432	-2.37995348919877
O	2.17245320563810	1.25713750262871	-2.44884701105044
O	0.20570166075571	0.36070756890744	-3.05812205174801
C	-0.07400230369892	1.53751853635233	-3.83632476154571
H	3.29241309416201	-0.18353685130389	0.39314451852337
H	2.16063563371682	0.27840405618271	1.67445396406705
H	2.26374428774189	1.23399512873448	0.19040245862527
H	1.09294835134154	-1.95588008207720	1.71213212059667
H	0.52640511584217	-2.72095732573067	0.22081546802578
H	2.25729247962101	-2.48405257026114	0.48747453503458
H	-1.05405161797368	1.37352227740869	-4.27282730011779
H	0.67470544784923	1.66375628893112	-4.61638987004658
H	-0.08434625018159	2.41651217368110	-3.19575026164829
C	1.52297146076661	-0.88838094524401	-1.55329300667049
H	2.55175028447075	-1.23045417174729	-1.66658230899382
H	0.84897238762590	-1.65701916375653	-1.92405502371681
H	-3.20924585611890	2.13185382842361	0.35075002280987
H	-3.12572365914591	2.86400868044972	1.95588236244472
H	-2.96847414861998	1.11309972619931	1.77305754384424
H	-0.51435558649630	2.36224752018980	3.58232844685488
H	-0.12507597752814	0.75291104597621	2.97075912948212
H	1.02783537843162	2.07752284068847	2.76827595859915
C	-0.72868833132421	5.00134785602384	1.65500104039241
C	-1.21876924799351	4.34645964572055	-0.71304501570086
C	1.09261915529571	4.06193210009476	0.21231095139572
H	-0.49372303181543	6.01537615997143	1.31579269332308
H	-0.14297779619581	4.81112641829643	2.55619506543053
H	-1.78538861744152	4.98295811049688	1.92932267912885
H	-2.29135368677788	4.35240740175080	-0.51227175655346
H	-0.94175575720937	5.34501787939290	-1.06680200985061
H	-1.03329096270485	3.64361340041110	-1.52619577329899
H	1.36095719663469	3.37574163053191	-0.59096572584325
H	1.70968334414430	3.82119157999172	1.08014199505958
H	1.35479827496044	5.07531447794629	-0.10894032838951

DFT Coordinates Data for compound 6

Coordinates from ORCA-job attack-02 Relaxed Surface Scan Step 1 E -803.683184153900

C	0.192145	0.023688	-0.108481
N	1.464286	0.224658	-0.370460
H	-0.493673	0.860137	-0.021923
H	-0.112700	-1.014709	0.005700
O	1.944046	1.628203	-0.534959

O	2.385981	-0.573122	-0.465140
C	0.945023	2.632817	-0.678096
H	0.358908	2.784751	0.247915
H	1.518629	3.550104	-0.889681
H	0.271556	2.422777	-1.531925
C	-0.100035	-0.040223	2.876573
C	0.064548	1.300216	2.794167
H	0.755994	-0.710884	2.982464
H	-1.099821	-0.483894	2.884038
O	1.235084	1.962138	2.763084
O	-0.942031	2.207732	2.647431
Si	2.800760	1.311050	2.957880
H	3.053869	0.225941	1.976576
H	3.696974	2.472326	2.749553
H	2.929944	0.787726	4.346877
H	-1.788296	1.730345	2.719374

Coordinates from ORCA-job attack-02 Relaxed Surface Scan Step 2 E -803.681936937356

C	-0.025044	0.007735	0.025103
N	1.288748	-0.008348	0.019819
H	-0.566420	0.947890	0.007543
H	-0.513359	-0.964531	0.017245
O	2.016536	1.297295	0.018869
O	2.067272	-0.950327	0.071162
C	1.248665	2.456185	-0.286065
H	0.521793	2.708401	0.509418
H	1.996692	3.263500	-0.349999
H	0.733842	2.362850	-1.262290
C	-0.860182	-0.094896	2.800352
C	-0.502784	1.206466	2.900408
H	-0.144246	-0.896233	2.997420
H	-1.891625	-0.377005	2.570389
O	0.729003	1.678461	3.163092
O	-1.325098	2.267022	2.663923
Si	2.114394	0.785585	3.604866
H	2.407283	-0.278669	2.612190
H	3.189096	1.803988	3.662348
H	1.885580	0.184861	4.948998
H	-2.225503	1.922545	2.523325

Coordinates from ORCA-job attack-02 Relaxed Surface Scan Step 3 E -803.680452327381

C	-0.047289	-0.015904	-0.035510
N	1.265196	-0.027434	0.016976
H	-0.591124	0.920614	-0.102388
H	-0.531717	-0.989339	-0.074502
O	1.988564	1.278298	0.044788
O	2.041649	-0.968536	0.118681
C	1.227814	2.438546	-0.268942
H	0.473403	2.677454	0.505056
H	1.974402	3.249222	-0.295097
H	0.746283	2.359664	-1.263380
C	-0.784486	-0.141351	2.662786
C	-0.474349	1.152201	2.915534
H	-0.033997	-0.930315	2.749217
H	-1.811680	-0.435659	2.429427

O	0.744612	1.634985	3.218822
O	-1.337806	2.201225	2.816744
Si	2.133455	0.751548	3.666444
H	2.493817	-0.250312	2.631939
H	3.179316	1.791181	3.815262
H	1.870107	0.076800	4.968341
H	-2.222649	1.843052	2.622383

Coordinates from ORCA-job attack-02 Relaxed Surface Scan Step 4 E -803.678562464847

C	-0.009747	-0.003198	0.040406
N	1.303661	-0.005370	0.014128
H	-0.561295	0.930857	0.020324
H	-0.488340	-0.977681	-0.029172
O	2.019079	1.305155	0.059490
O	2.090600	-0.944253	0.043791
C	1.237156	2.468919	-0.176222
H	0.520989	2.676503	0.642326
H	1.975978	3.286357	-0.212895
H	0.705748	2.420638	-1.147080
C	-0.609117	-0.247125	2.661714
C	-0.312178	1.025913	3.016968
H	0.152670	-1.029824	2.674827
H	-1.640497	-0.540811	2.446863
O	0.909112	1.503771	3.317757
O	-1.193938	2.064082	3.027111
Si	2.322834	0.612424	3.660378
H	2.665616	-0.303927	2.543761
H	3.357577	1.655564	3.855760
H	2.107222	-0.160358	4.915730
H	-2.079949	1.706459	2.837274

Coordinates from ORCA-job attack-02 Relaxed Surface Scan Step 5 E -803.676280744604

C	-0.022402	-0.014939	0.014288
N	1.292123	-0.011103	0.008025
H	-0.578405	0.915764	-0.032134
H	-0.493943	-0.988245	-0.104411
O	2.000930	1.299123	0.095557
O	2.080233	-0.950272	0.051020
C	1.218371	2.465068	-0.124558
H	0.485104	2.648019	0.684821
H	1.953523	3.286615	-0.125297
H	0.706063	2.441325	-1.106684
C	-0.610209	-0.293663	2.531587
C	-0.336703	0.947032	3.005758
H	0.152805	-1.075456	2.525644
H	-1.635737	-0.580226	2.282069
O	0.870751	1.405350	3.382702
O	-1.226267	1.977221	3.065683
Si	2.270077	0.491979	3.726782
H	2.672343	-0.331748	2.558906
H	3.287632	1.518518	4.055688
H	1.994661	-0.378272	4.904196
H	-2.102438	1.631223	2.817595

Coordinates from ORCA-job attack-02 Relaxed Surface Scan Step 6 E -803.673498209293

C	-0.006141	-0.003815	0.052357
N	1.309230	0.000360	0.014738
H	-0.558381	0.930164	0.022483
H	-0.478143	-0.965895	-0.136871
O	2.020499	1.305423	0.162737
O	2.096356	-0.942576	0.012562
C	1.241865	2.479930	-0.019581
H	0.509512	2.640194	0.795848
H	1.978897	3.299561	0.004092
H	0.727868	2.489096	-1.001197
C	-0.584513	-0.391551	2.453429
C	-0.320944	0.806221	3.036044
H	0.172373	-1.179008	2.425329
H	-1.609435	-0.661475	2.183641
O	0.880103	1.231168	3.466251
O	-1.210068	1.831502	3.154172
Si	2.277612	0.295332	3.754435
H	2.693729	-0.434731	2.530424
H	3.288681	1.294256	4.175482
H	1.990201	-0.663964	4.857566
H	-2.083381	1.505765	2.871174

Coordinates from ORCA-job attack-02 Relaxed Surface Scan Step 7 E -803.670275753715

C	-0.010799	-0.005012	0.045468
N	1.307087	0.000660	0.012990
H	-0.557832	0.932526	0.010672
H	-0.476498	-0.950586	-0.226918
O	2.015593	1.297023	0.239827
O	2.092303	-0.946011	-0.004779
C	1.247394	2.478821	0.064220
H	0.487976	2.619863	0.858753
H	1.984574	3.295725	0.134545
H	0.765737	2.516820	-0.933102
C	-0.625580	-0.477783	2.316708
C	-0.388975	0.667333	3.012438
H	0.121661	-1.274836	2.289428
H	-1.644633	-0.730279	2.009748
O	0.792503	1.051810	3.524498
O	-1.280475	1.685964	3.164267
Si	2.181458	0.096794	3.791788
H	2.641576	-0.540717	2.532265
H	3.173512	1.061660	4.323148
H	1.858025	-0.941185	4.810698
H	-2.142131	1.384580	2.824348

Coordinates from ORCA-job attack-02 Relaxed Surface Scan Step 8 E -803.666660936067

C	0.013049	-0.036468	0.058986
N	1.334021	0.025365	0.032337
H	-0.562364	0.883010	-0.013271
H	-0.402949	-0.982909	-0.285422
O	1.978529	1.339178	0.333961
O	2.155797	-0.893008	0.019259
C	1.189899	2.496010	0.096048
H	0.371530	2.618308	0.833949
H	1.893529	3.337023	0.211614

H	0.777688	2.513092	-0.932587
C	-0.645325	-0.532288	2.206241
C	-0.407523	0.543585	3.013076
H	0.044009	-1.381148	2.212115
H	-1.663745	-0.722564	1.854248
O	0.744269	0.823944	3.641734
O	-1.258007	1.597052	3.167101
Si	2.109627	-0.181118	3.842066
H	2.649185	-0.604131	2.525203
H	3.061228	0.667479	4.598405
H	1.711913	-1.370702	4.646970
H	-2.105912	1.362927	2.748783

Coordinates from ORCA-job attack-02 Relaxed Surface Scan Step 9 E -803.662864687813

C	-0.021007	0.001384	0.057028
N	1.307061	0.001341	0.019252
H	-0.537725	0.959242	0.036557
H	-0.475998	-0.865014	-0.425126
O	2.007501	1.240502	0.487158
O	2.078138	-0.960064	-0.062412
C	1.315100	2.461668	0.275497
H	0.460332	2.598297	0.969197
H	2.062192	3.245335	0.485035
H	0.970617	2.567035	-0.772598
C	-0.711301	-0.643527	2.043881
C	-0.463022	0.323702	2.984703
H	-0.072900	-1.531808	2.008255
H	-1.737255	-0.768772	1.682455
O	0.677347	0.487167	3.668261
O	-1.276884	1.390039	3.221073
Si	2.017167	-0.567520	3.775637
H	2.537518	-0.897603	2.425044
H	2.991328	0.189541	4.597516
H	1.587484	-1.805666	4.485694
H	-2.120639	1.233206	2.760415

Coordinates from ORCA-job attack-02 Relaxed Surface Scan Step 10 E -803.668635587740

C	-0.600615	0.137596	0.216245
N	0.659583	0.502744	-0.016306
H	-1.292992	0.964015	0.382598
H	-0.920308	-0.729338	-0.365003
O	0.923171	1.827571	0.648593
O	1.638571	-0.197972	-0.340828
C	2.098514	2.422225	0.114947
H	1.893311	2.955675	-0.835521
H	2.438263	3.141269	0.877805
H	2.876097	1.655894	-0.052616
C	-0.798789	-0.904450	2.028665
C	-0.671835	-0.122846	3.161039
H	0.021229	-1.587977	1.781224
H	-1.802969	-1.269203	1.784174
O	0.477786	0.185802	3.772789
O	-1.682515	0.586853	3.721777
Si	2.022192	-0.498339	3.533147
H	2.428698	-0.388349	2.108201

H	2.898347	0.281581	4.439694
H	1.982540	-1.929367	3.946627
H	-2.510828	0.347958	3.268502

Coordinates from ORCA-job attack-02 Relaxed Surface Scan Step 11 E -803.665475869658

C	-0.034539	0.001164	0.029911
N	1.309570	-0.001036	-0.010759
H	-0.473176	1.001466	-0.003365
H	-0.473752	-0.770986	-0.607538
O	1.813371	1.251267	0.667731
O	2.076400	-0.989192	-0.060764
C	3.184967	1.452241	0.359335
H	3.316356	1.948719	-0.623939
H	3.580237	2.101619	1.157459
H	3.722665	0.486946	0.359562
C	-0.752829	-0.766493	1.731311
C	-0.637782	0.030370	2.868120
H	-0.125744	-1.667077	1.694353
H	-1.773355	-0.912055	1.355047
O	0.417480	0.076723	3.684577
O	-1.515071	1.007796	3.203355
Si	1.752496	-0.986285	3.768712
H	2.422214	-1.083564	2.446588
H	2.612158	-0.385558	4.816535
H	1.262994	-2.326064	4.199532
H	-2.274740	0.958765	2.595653

Coordinates from ORCA-job attack-02 Relaxed Surface Scan Step 12 E -803.663550517056

C	-0.049016	-0.001040	0.010504
N	1.313270	-0.007820	-0.034700
H	-0.463740	1.011894	-0.017546
H	-0.474018	-0.709794	-0.708880
O	1.787146	1.197870	0.771399
O	2.040544	-1.034024	-0.013007
C	3.172283	1.401615	0.546559
H	3.359016	1.968995	-0.388631
H	3.542072	1.981184	1.408891
H	3.697311	0.429515	0.498150
C	-0.721018	-0.817929	1.588827
C	-0.650733	-0.084257	2.788137
H	-0.080498	-1.712476	1.544666
H	-1.746640	-0.999565	1.237396
O	0.367133	-0.081749	3.643962
O	-1.560547	0.851033	3.148116
Si	1.715518	-1.135231	3.725958
H	2.417658	-1.163606	2.418446
H	2.524713	-0.571637	4.832907
H	1.220995	-2.494185	4.083986
H	-2.284275	0.844208	2.495937

2 Supplementary References

1. Zhu, C. *et al.* Catalytic Asymmetric Synthesis of Unprotected β²-Amino Acids. *J. Am. Chem. Soc* .**143**, 3312–3317 (2021).
2. Gatzenmeier, T. *et al.* Scalable and Highly Diastereo- and Enantioselective Catalytic Diels–Alder Reaction of α,β-Unsaturated Methyl Esters. *J. Am. Chem. Soc.* **140**, 12671–12676 (2018).
3. Kaib, S. J. P. *et al*. Extremely Active Organocatalysts Enable a Highly Enantioselective Addition of Allyltrimethylsilane to Aldehydes. *Angew. Chem. Int. Ed.* **55**, 13200–13203 (2016).
4. Ghosh, S. *et al*. Strong and Confined Acids Control Five Stereogenic Centers in Catalytic Asymmetric Diels–Alder Reactions of Cyclohexadienones with Cyclopentadiene. *Angew. Chem. Int. Ed.* **59**, 12347–12351 (2020).
5. Lee, S. *et at.* Asymmetric Catalysis via Cyclic, Aliphatic Oxocarbenium Ions. *J. Am. Chem. Soc.* **139**, 2156–2159 (2017).
6. Tsuji, N. *et al.* Predicting Highly Enantioselective Catalysts Using Tunable Fragment Descriptors. *Angew. Chem. Int. Ed.* **62**, e202218659 (2023).
7. Maji, R. *et al.* A Catalytic Asymmetric Hydrolactonization. *J. Am. Chem. Soc.* **145**, 8788–8793 (2023).
8. Zhou, H., Zhou, Y., Bae, H.Y. *et al.* Organocatalytic stereoselective cyanosilylation of small ketones. *Nature* **605**, 84–89 (2022).
9. Neese, F. The ORCA program system. *WIREs Comput. Mol. Sci.* **2**, 73–78 (2012).
10. Bannwarth, C. *et al*. GFN2-xTB-An Accurate and Broadly Parametrized Self-Consistent Tight-Binding Quantum Chemical Method with Multipole Electrostatics and Density-Dependent Dispersion Contributions. *J. Chem. Theory Comput*. **15**, 1652-1671 (2019).
11. Pracht, P. *et al*. Automated exploration of the low-energy chemical space with fast quantum chemical methods. *Phys. Chem. Chem. Phys.* **22**, 7169-7192 (2020).
12. Becke, A. D. A new mixing of Hartree–Fock and local density-functional theories. *J. Chem. Phys.* **98**, 1372–1377 (1993).
13. Caldeweyher, E. *et al.* Extension of the D3 dispersion coefficient model, *J.Chem.Phys*. **147**, 034112 (2017).
14. Caldeweyher, E. *et al*. A generally applicable atomic-charge dependent London dispersion correction, *J.Chem.Phys*. **150**, 154122 (2018).
15. Weigend, F. and Ahlrichs, R. Balanced basis sets of split valence, triple zeta valence and quadruple zeta valence quality for H to Rn: Design and assessment of accuracy, *Phys. Chem. Chem. Phys.* **7**, 3297 (2005).
16. Valeev, E. F. Libint: A library for the evaluation of molecular integrals of many-body operators over Gaussian functions, Can be found at: http://libint.valeyev.net/
17. Chai, J.-D. *et al*. Long-range corrected hybrid density functionals with damped atom–atom dispersion corrections. *J. Chem. Phys.* **128**, 084106 (2008).
18. Barone, V. and Cossi, M. Quantum calculation of molecular energies and energy gradients in solution by a conductor solvent model. *J. Phys. Chem. A*. **102**, 1995–2001 (1998).

19. Zhao, Y. and Truhlar, D.G. The M06 suite of density functionals for main group thermochemistry, thermochemical kinetics, noncovalent interactions, excited states, and transition elements: two new functionals and systematic testing of four M06-class functionals and 12 other functionals. *Theor. Chem. Account.* **120**, 215–241 (2008).
20. Lu, T. F. *et al*. Multiwfn: A multifunctional wavefunction analyzer. *Comput. Chem.* **61**, 580-592 (2012). Xtb, Version 6.3; University Bonn: 2020; please refer to: xtb@thch.uni-bonn.de. T. Lu, molclus program, Can be found at: http://www.keinsci.com/research
21. Maeda, S., Harabuchi, Y., Takagi, M., Taketsugu, T. & Morokuma, K. Exploring paths of chemical transformations in complex systems: automated reaction path search methods. *Chem. Rec.* **16**, 2232–2248 (2016).
22. Maeda, S., Ohno, K. & Morokuma, K. Systematic exploration of the mechanism of chemical reactions: the global reaction route mapping (GRRM) strategy using the ADDF and AFIR methods. *Phys. Chem. Chem. Phys.* **15**, 3683–3701 (2013).
23. Anstine, D. M., Zubatyuk, R. & Isayev, O. AIMNet2: a neural network potential to meet your neutral, charged, organic, and elemental-organic needs. *Chem. Sci.* **16**, 10228–10244 (2025).
24. Neese, F. Software update: The ORCA program system — Version 5.0. *WIREs Computational Molecular Science* **12**, e1606 (2022).
25. Neese, F. Software update: the ORCA program system — Version 6.0. *WIREs Comput. Mol. Sci.* **15**, e70019 (2025).
26. Mardirossian, N. & Head-Gordon, M. ωB97X-V: a 10-parameter, range-separated hybrid, generalized gradient approximation density functional with nonlocal correlation. *Phys. Chem. Chem. Phys.* **16**, 9904–9924 (2014).
27. Weigend, F. & Ahlrichs, R. Balanced basis sets of split valence, triple zeta valence and quadruple zeta valence quality for H to Rn: design and assessment of accuracy. *Phys. Chem. Chem. Phys.* **7**, 3297–3305 (2005).
28. Lu, T. & Chen, Q. Independent gradient model based on Hirshfeld partition: IGMH. *J. Comput. Chem.* **43**, 2022–2037 (2022).
29. Lu, T. & Chen, F. Multiwfn: A multifunctional wavefunction analyzer. *J. Comput. Chem.* **33**, 580–592 (2012).
30. Frisch, M. J. *et al.* Gaussian 16, Revision A.03. Gaussian, Inc., Wallingford, CT (2016).
31. Meng, E. C., Pettersen, E. F., Huang, C. C., Cummings, P. D., Greenblatt, D. M., Ferrin, T. E. UCSF ChimeraX: Tools for structure building and analysis. *Protein Sci.* **32**, e4792 (2023).
32. Peng, Q., Duarte, F. & Paton, R. S. Computing organic stereoselectivity – from concepts to quantitative calculations and predictions. *Chem. Soc. Rev.* **45**, 6093–6107 (2016).
33. Lu, T. & Chen, Q. Independent gradient model based on Hirshfeld partition: IGMH. *J. Comput. Chem.* **43**, 2022–2037 (2022).
34. Lu, T. Independent gradient model based on Hirshfeld partition: IGMH. *J. Chem. Phys.* **161**, 082503 (2024).
35. Bickelhaupt, F. M. Analyzing reaction rates with the distortion/interaction–activation strain model. *Angew. Chem. Int. Ed.* **56**, 10070–10086 (2017).

36. Neese, F.; Wennmohs, F.; Becker, U.; Riplinger, C. The ORCA quantum chemistry program package. *J. Chem. Phys.* **152**, 224108 (2020).
37. Allinger, N. L.; Young, Y. H.; Lii, J. H. Molecular mechanics. The MM3 force field for hydrocarbons. *J. Am. Chem. Soc.* **111**, 8551–8566 (1989).
38. Perdew, J. P.; Burke, K.; Ernzerhof, M. PBE. Generalized gradient approximation made simple. *Phys. Rev. Lett.* **77**, 3865–3868 (1996).
39. Eichkorn, K.; Weigend, F.; Treutler, O.; Ahlrichs, R. Auxiliary basis sets for main row atoms and transition metals and their use to approximate coulomb potentials. *Theor. Chem. Acc.* **97**, 119–124 (1997).
40. Becke, A. D. B88 functional. Density-functional exchange-energy approximation with correct asymptotic behavior. *Phys. Rev. A* **38**, 3098–3100 (1988).
41. Lee, C.; Yang, W.; Parr, R. G. Development of the Colle–Salvetti correlation-energy formula into a functional of the electron density. *Phys. Rev. B* **37**, 785–789 (1988).
42. Stephens, P. J.; Devlin, F. J.; Chabalowski, C. F.; Frisch, M. J. Ab initio calculation of vibrational absorption and circular dichroism spectra using density functional force fields. *J. Phys. Chem.* **98**, 11623–11627 (1994).
43. Barone, V.; Cossi, M. Quantum calculation of molecular energies and energy gradients in solution by a conductor solvent model. *J. Phys. Chem. A* **102**, 1995–2001 (1998).

3 NMR Spectra

^{1}H NMR and ^{13}C NMR of **6k**

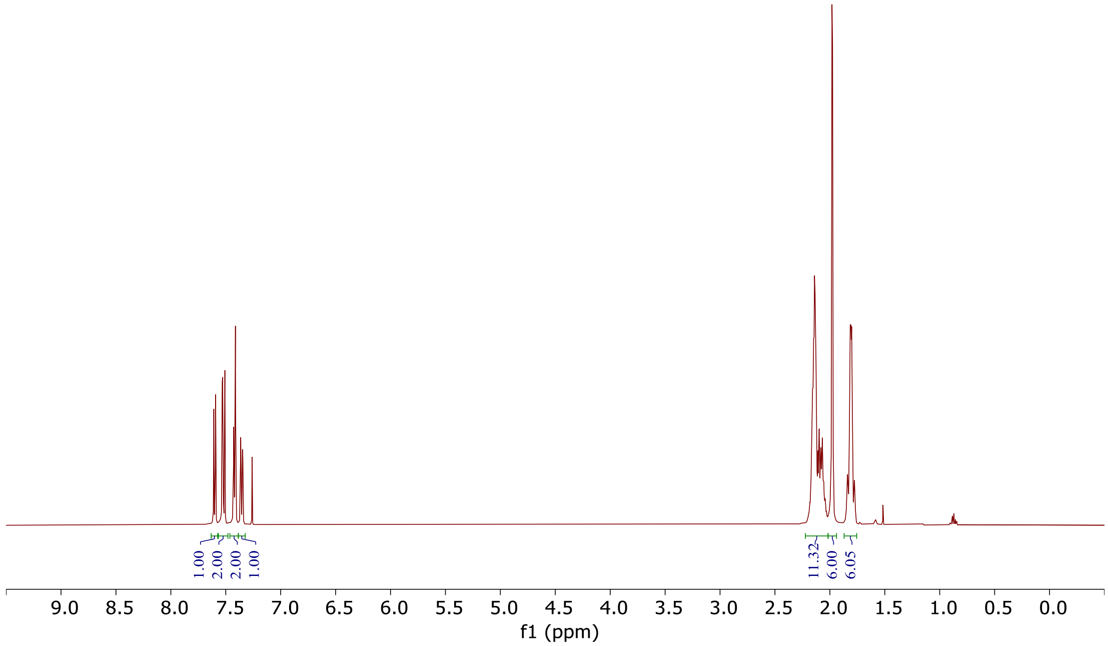

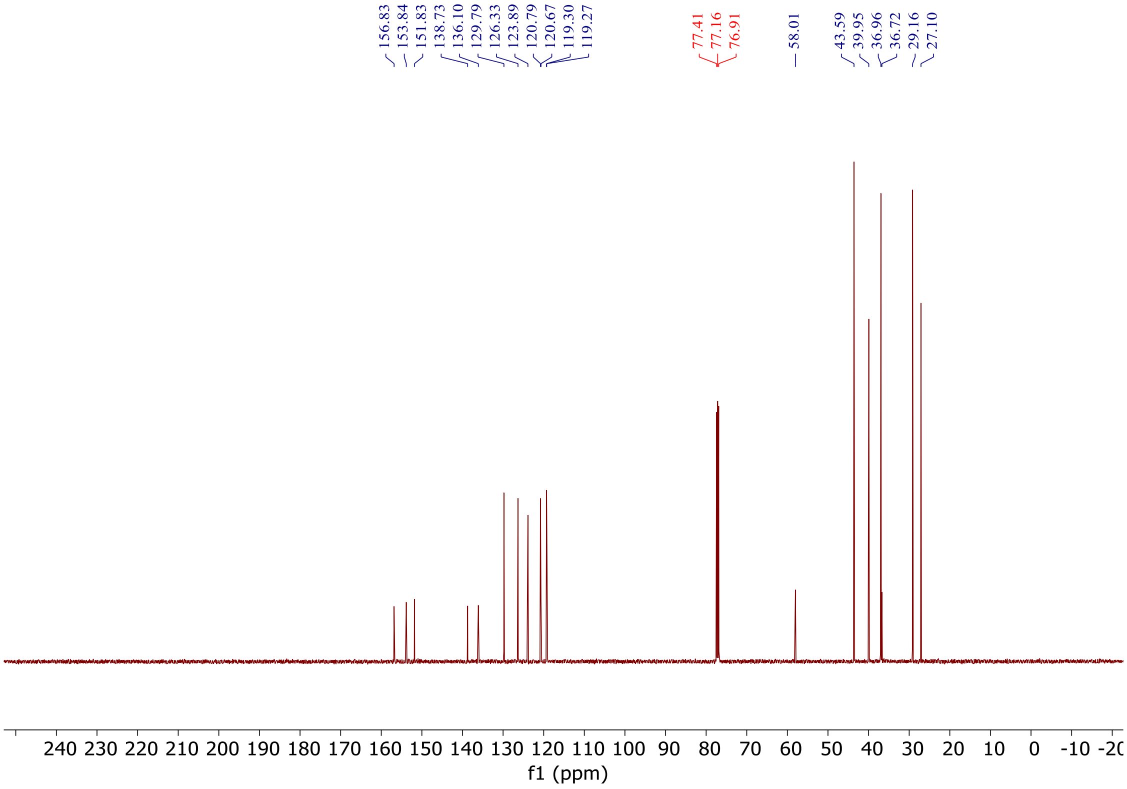

^{1}H NMR and ^{13}C NMR of (*S*)-**5k**

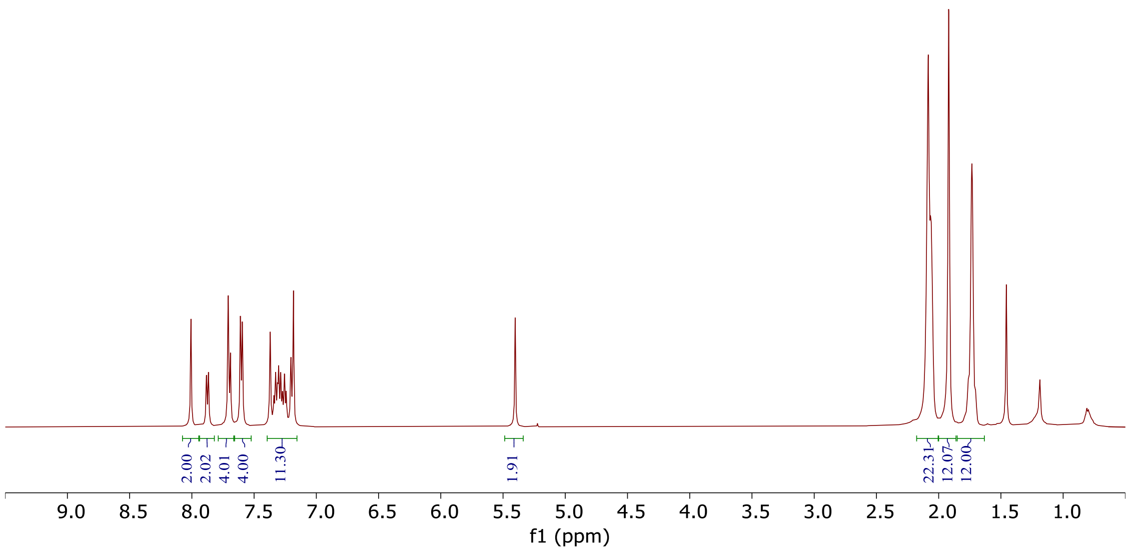

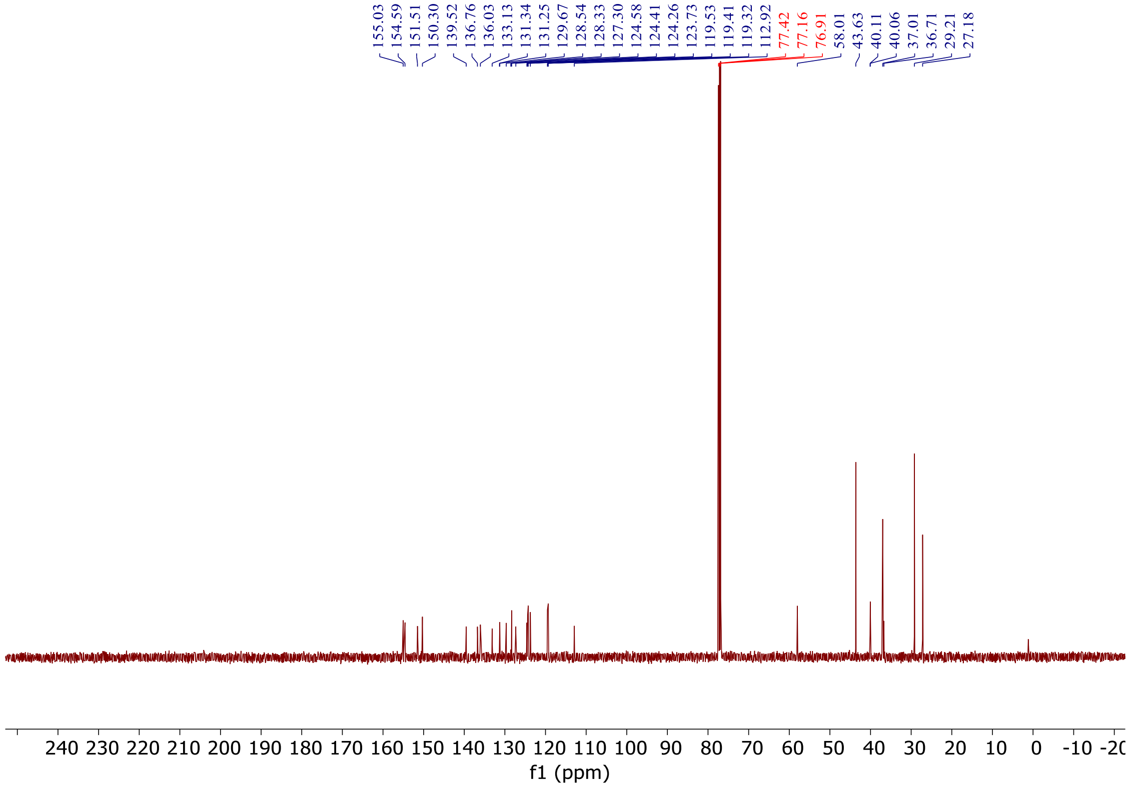

^{1}H NMR, ^{13}C NMR, ^{19}F NMR and ^{31}P NMR of (*S*,*S*)-**4j**

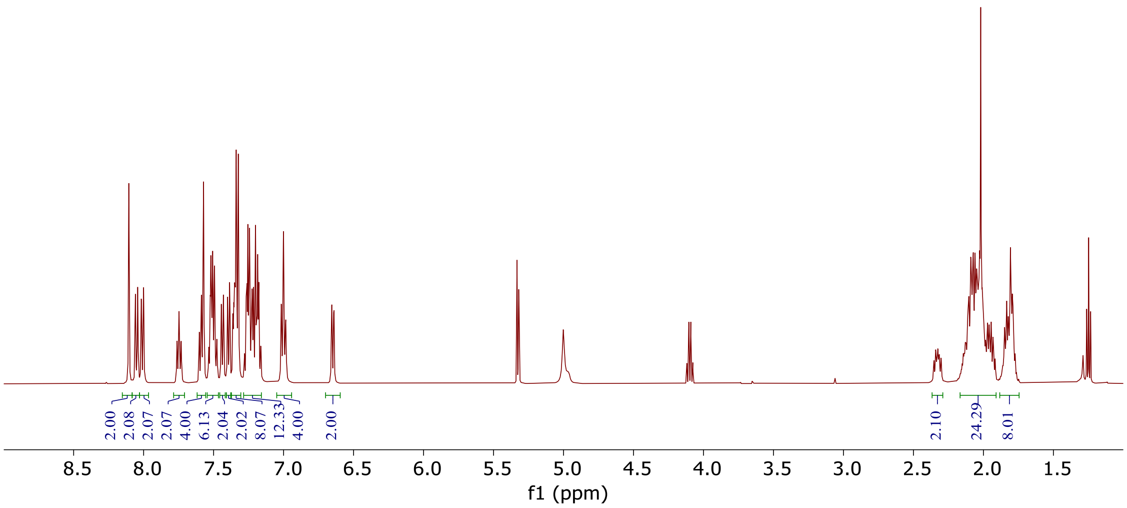

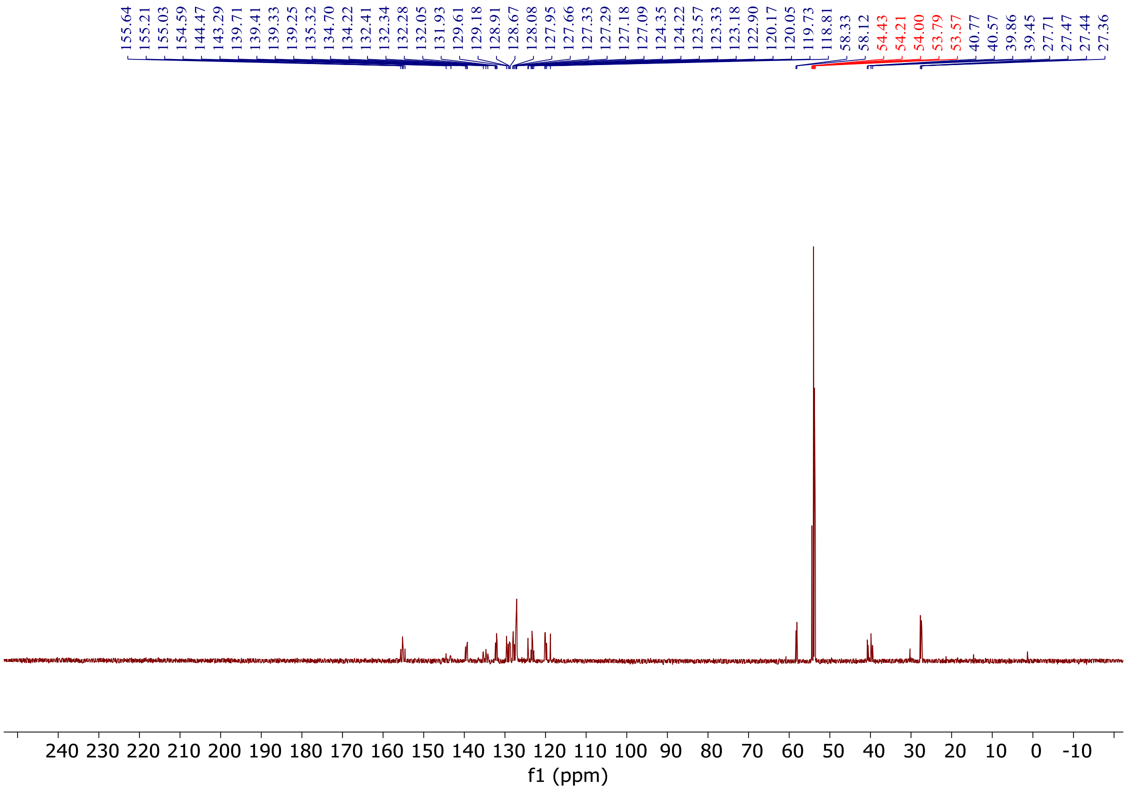

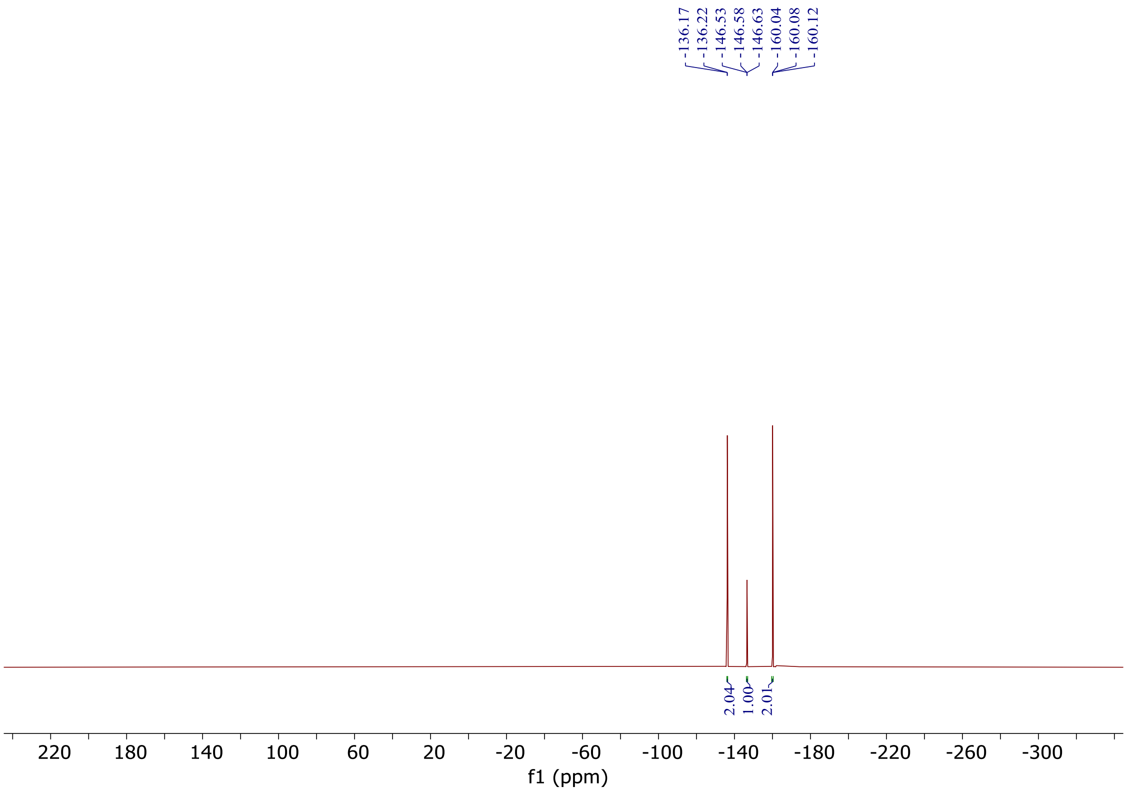
-136.17
-136.22
-146.53
-146.58
-146.63
-160.04
-160.08
-160.12
2.04
1.00
2.01
220 180 140 100 60 20 -20 -60 -100 -140 -180 -220 -260 -300
f1 (ppm)

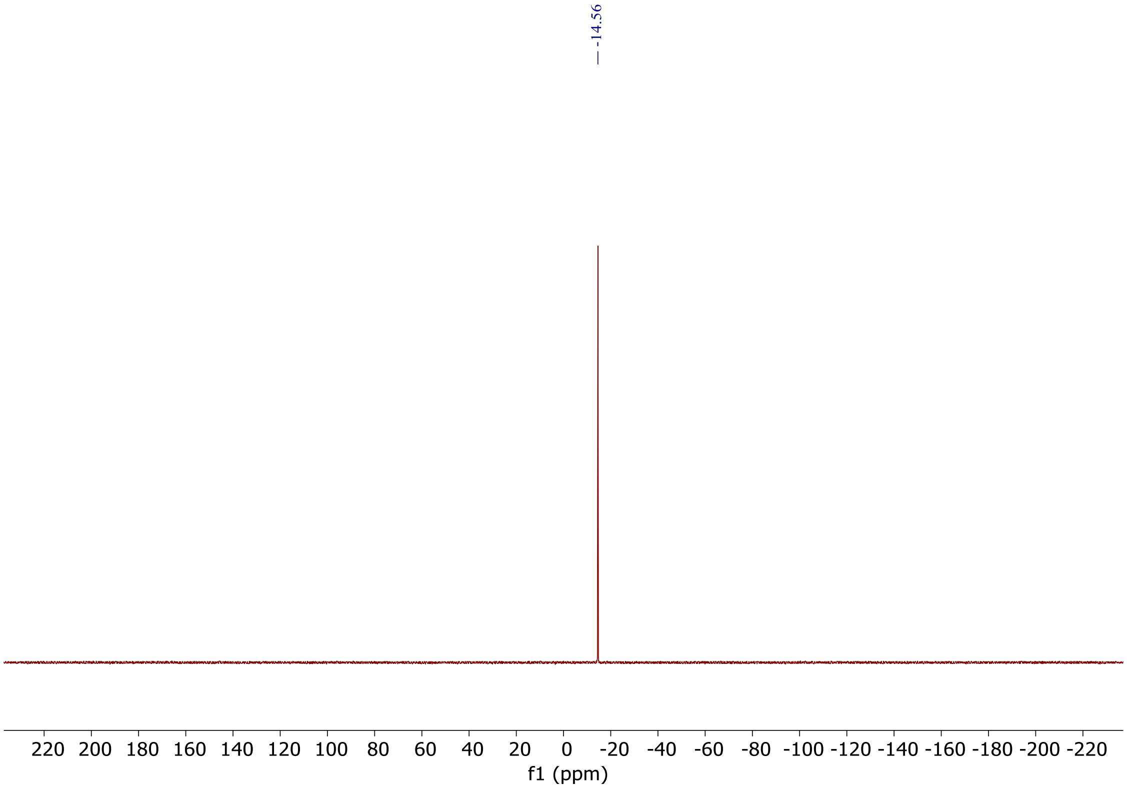
-14.56
220 200 180 160 140 120 100 80 60 40 20 0 -20 -40 -60 -80 -100 -120 -140 -160 -180 -200 -220
f1 (ppm)

^{1}H NMR, ^{13}C NMR, ^{19}F NMR and ^{31}P NMR of (*S*,*S*)-**4k**

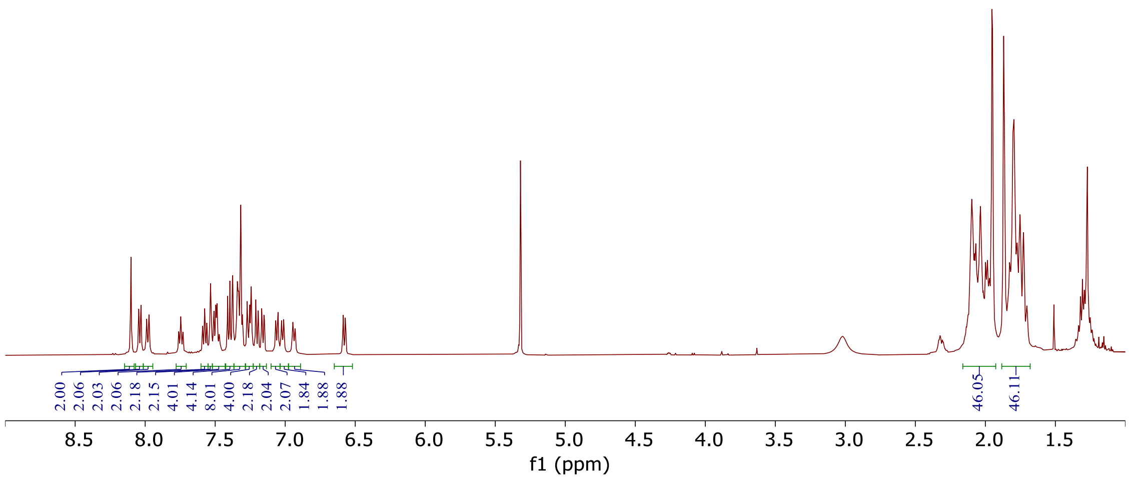

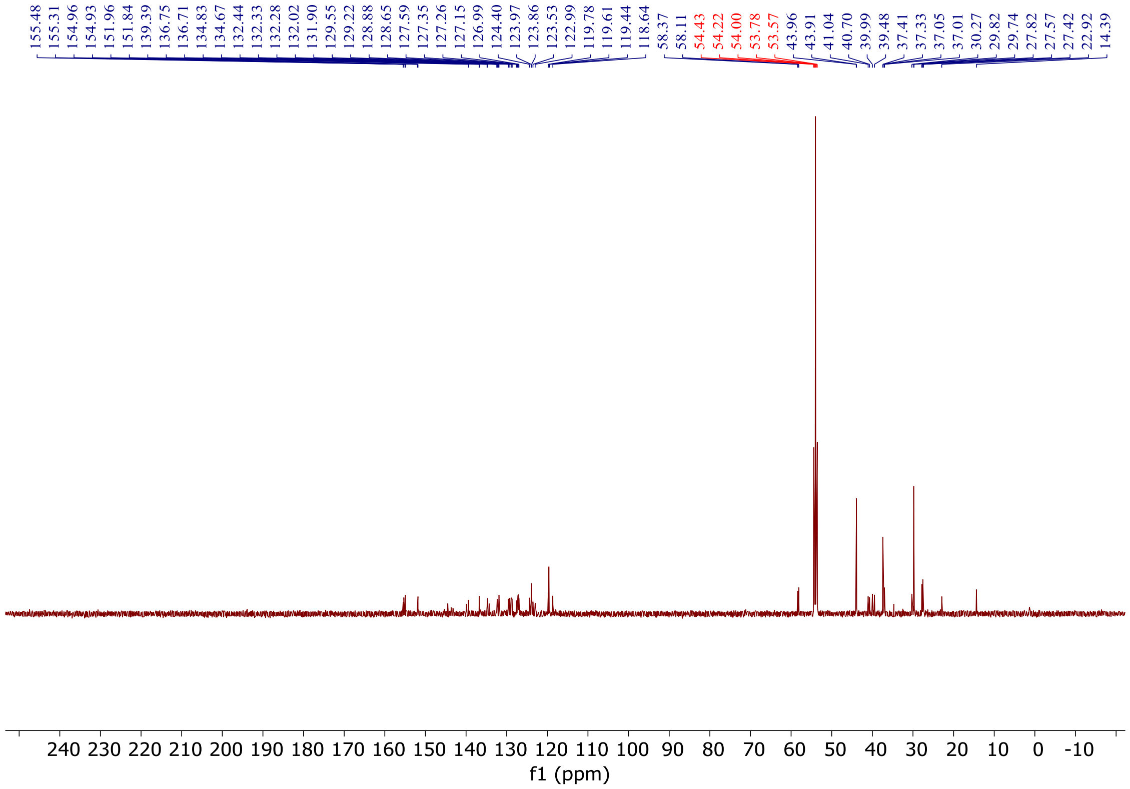

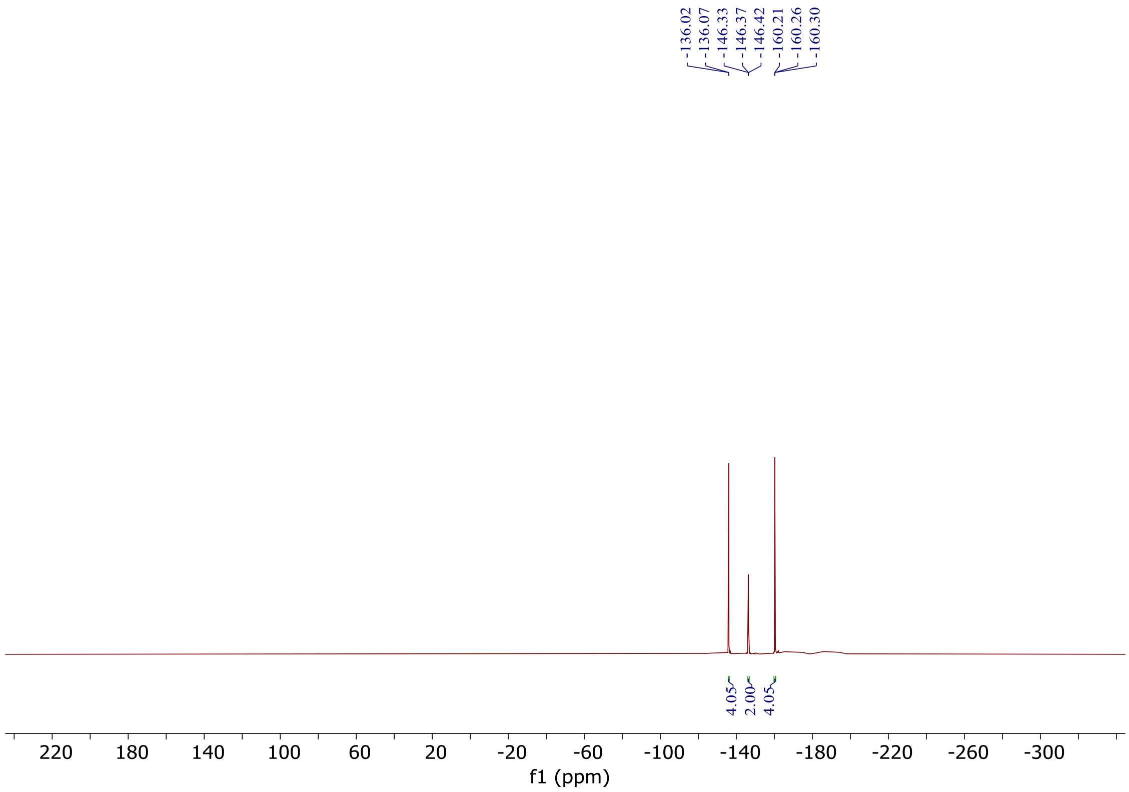
-136.02
-136.07
-146.33
-146.37
-146.42
-160.21
-160.26
-160.30
4.05
2.00
4.05
220 180 140 100 60 20 -20 -60 -100 -140 -180 -220 -260 -300
f1 (ppm)

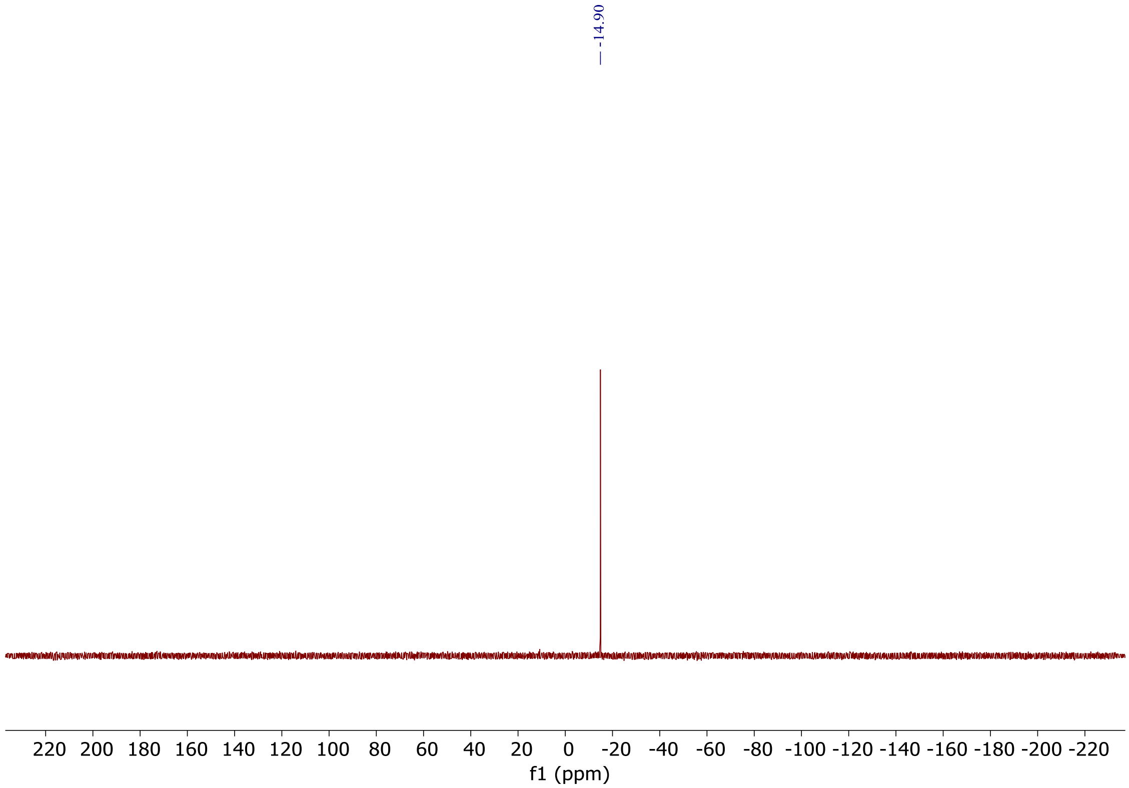
-14.90
220 200 180 160 140 120 100 80 60 40 20 0 -20 -40 -60 -80 -100 -120 -140 -160 -180 -200 -220
f1 (ppm)

^{1}H NMR, ^{13}C NMR, ^{19}F NMR and ^{31}P NMR of (*S*,*S*)-**4n**

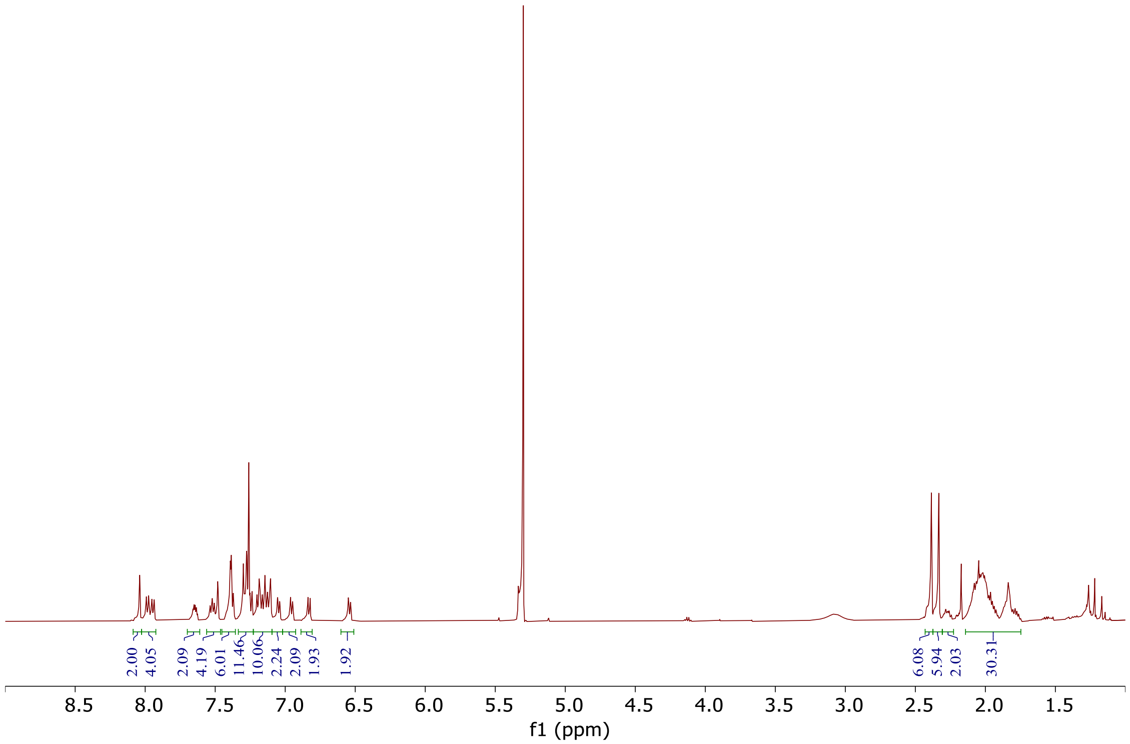

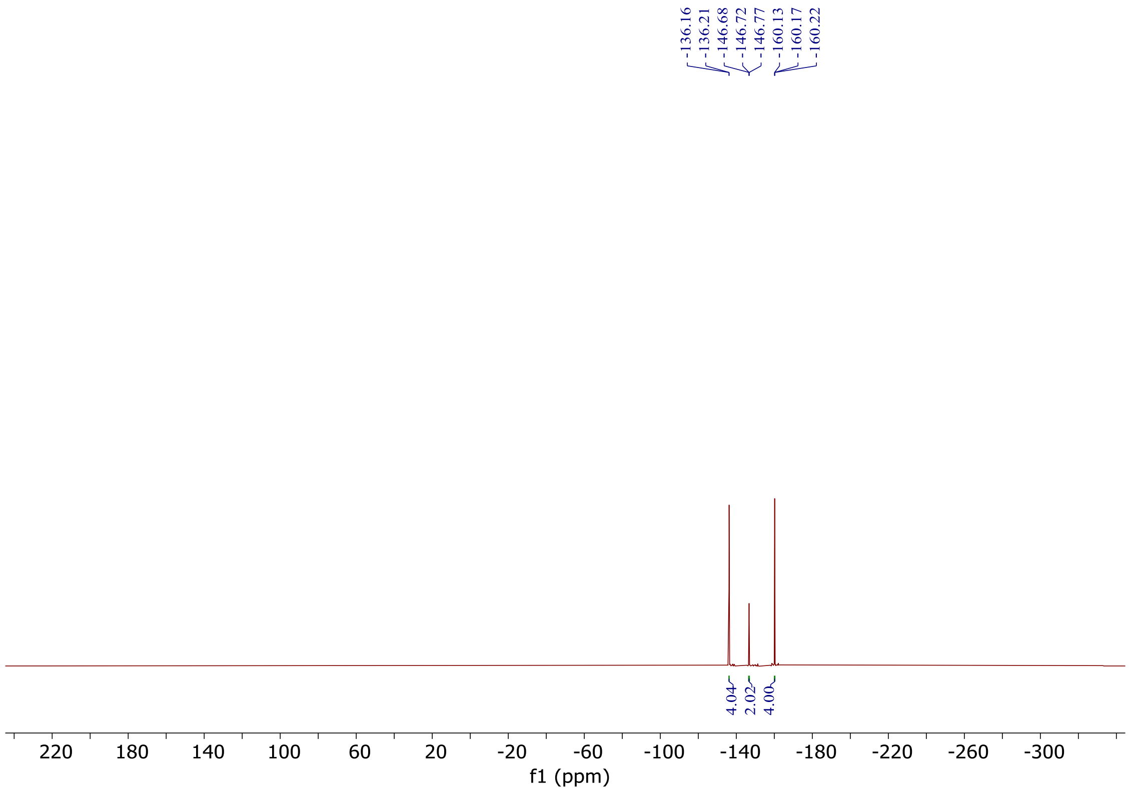
-136.16
-136.21
-146.68
-146.72
-146.77
-160.13
-160.17
-160.22
4.04
2.02
4.00
220 180 140 100 60 20 -20 -60 -100 -140 -180 -220 -260 -300
f1 (ppm)

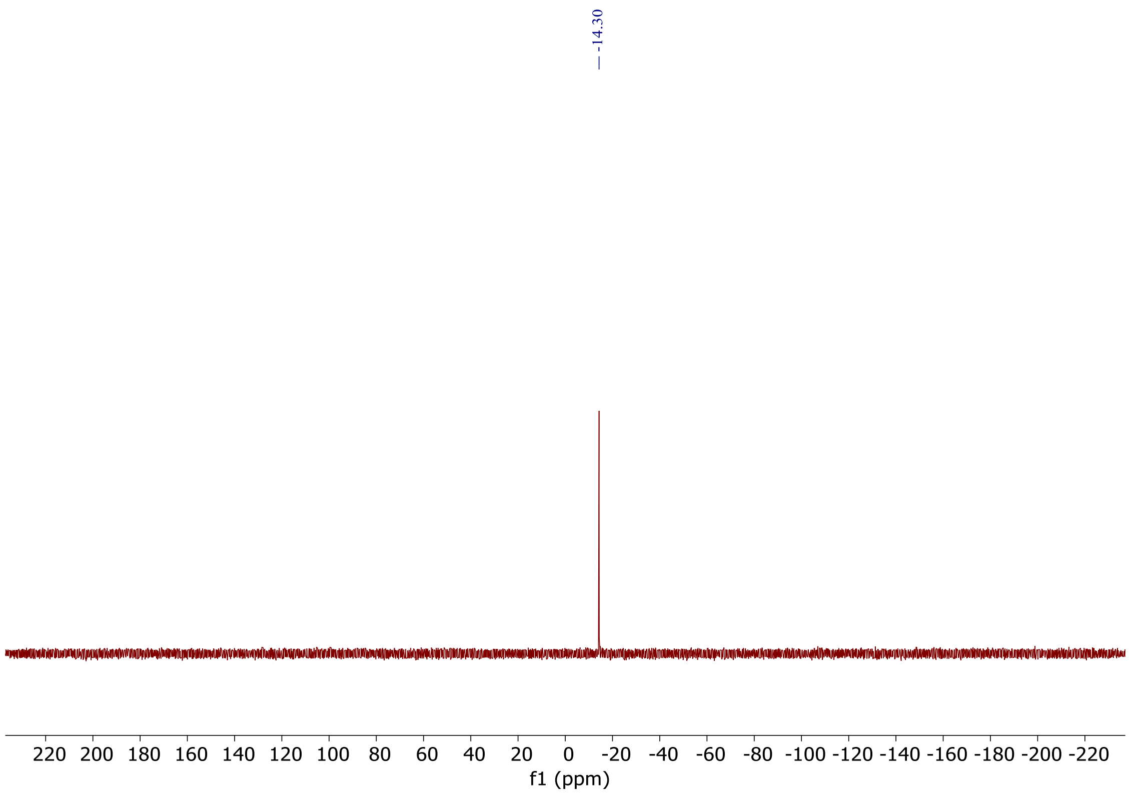
-14.30
220 200 180 160 140 120 100 80 60 40 20 0 -20 -40 -60 -80 -100 -120 -140 -160 -180 -200 -220
f1 (ppm)

^{1}H NMR, ^{13}C NMR, ^{19}F NMR and ^{31}P NMR of (*S*,*S*)-**4o**

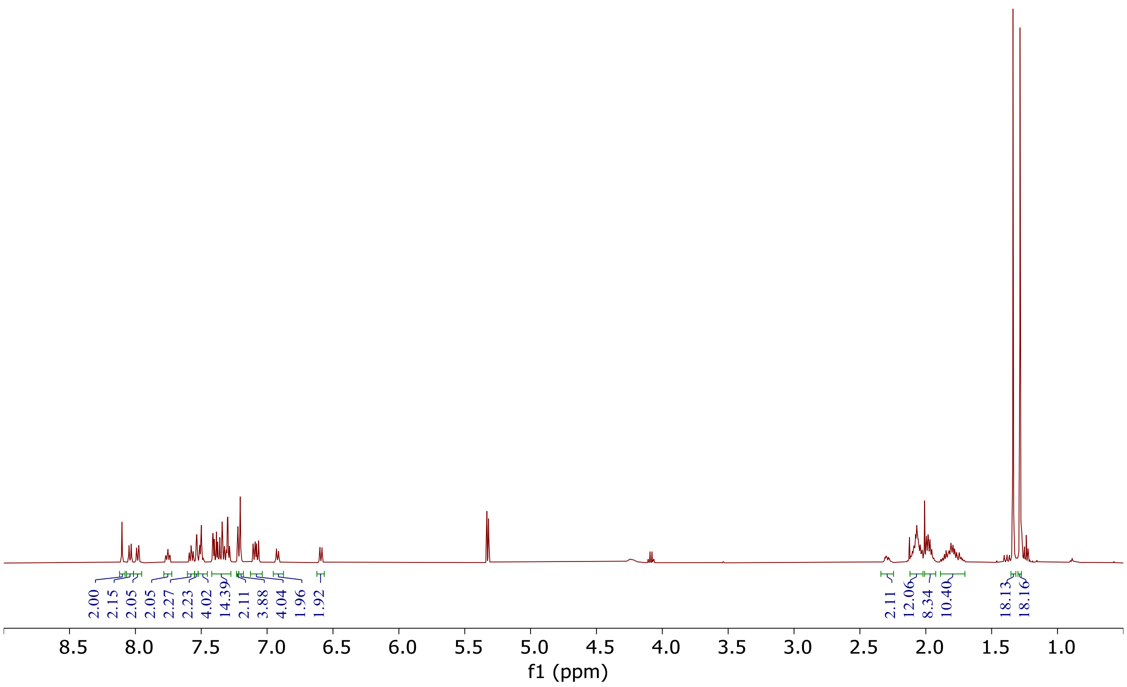

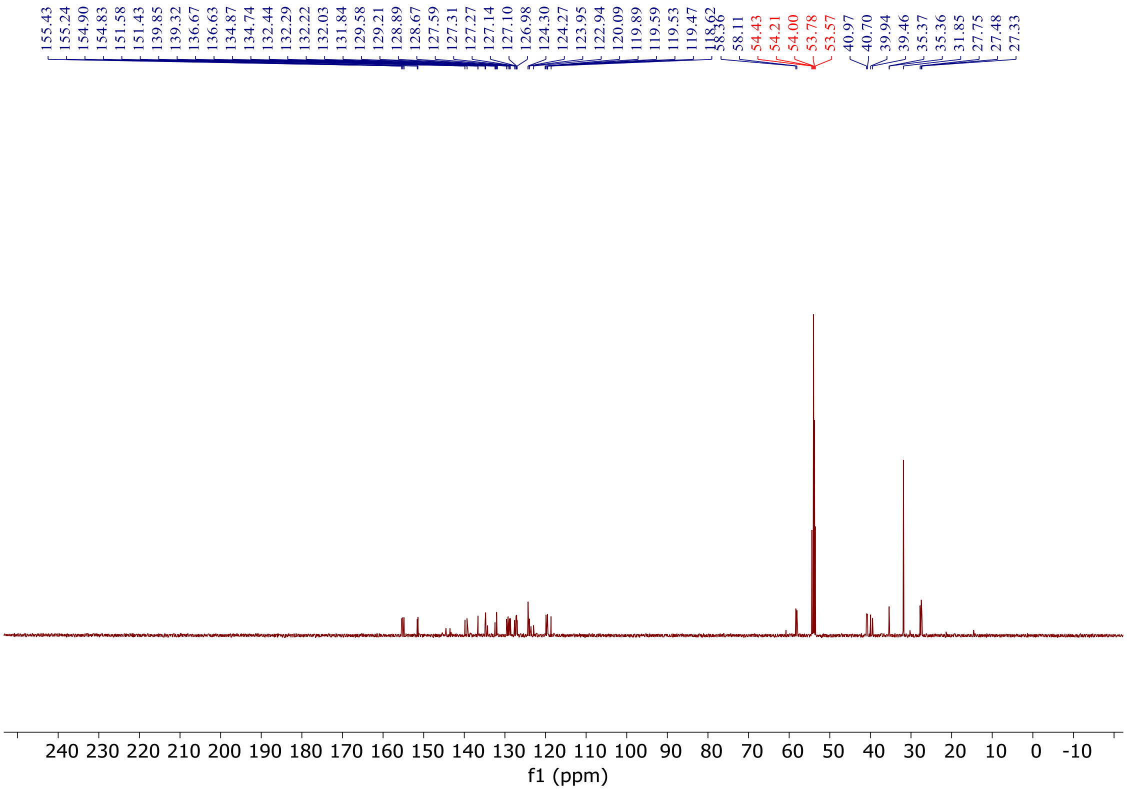

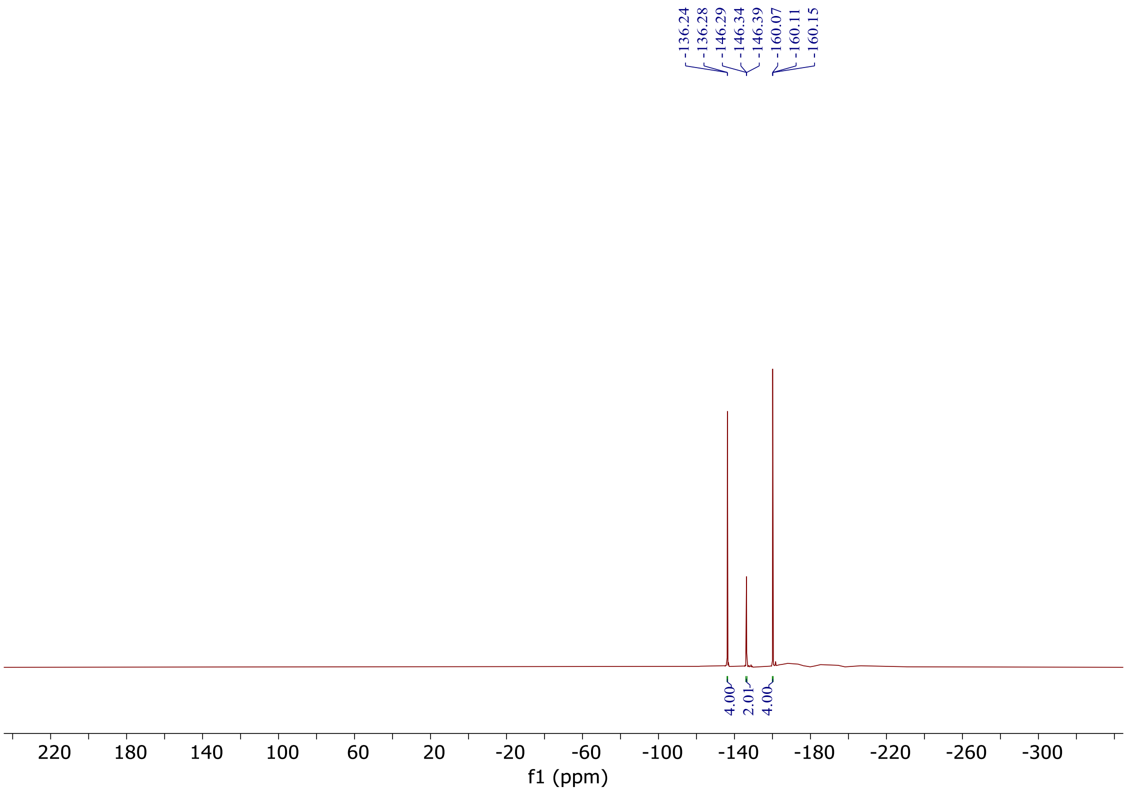

-136.24
-136.28
-146.29
-146.34
-146.39
-160.07
-160.11
-160.15
4.00
2.01
4.00
220 180 140 100 60 20 -20 -60 -100 -140 -180 -220 -260 -300
f1 (ppm)

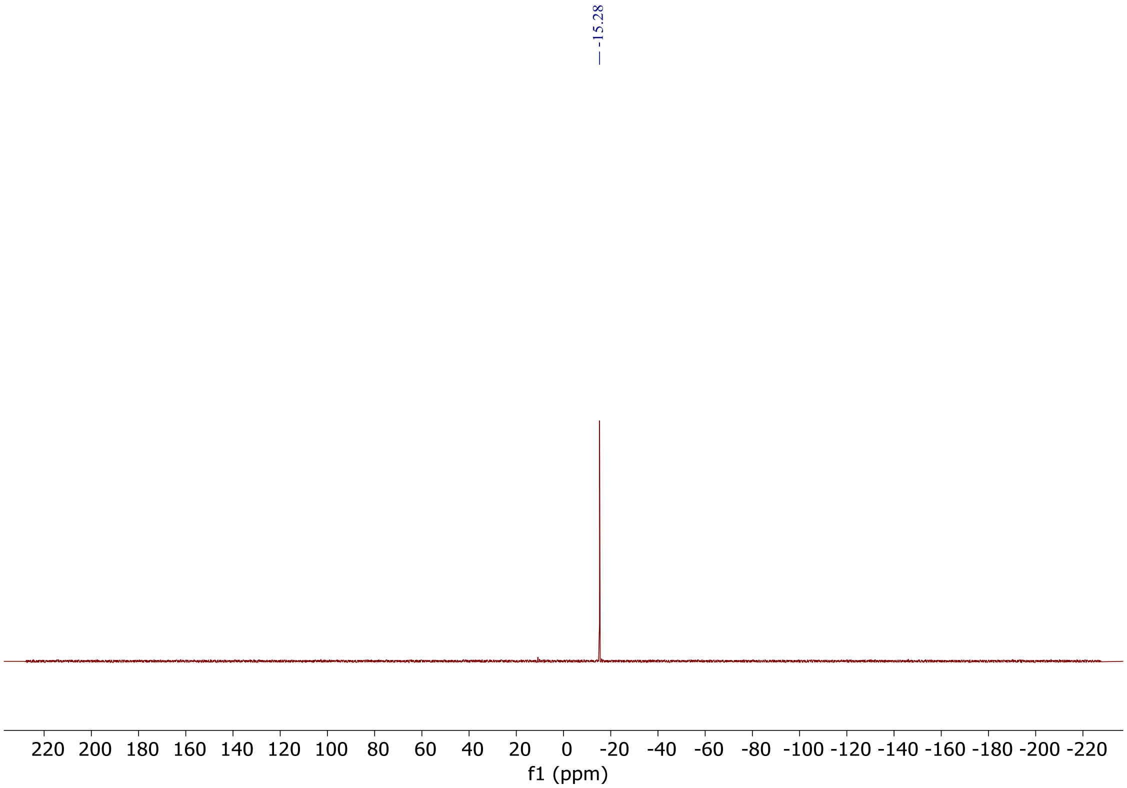

-15.28
220 200 180 160 140 120 100 80 60 40 20 0 -20 -40 -60 -80 -100 -120 -140 -160 -180 -200 -220
f1 (ppm)

^{1}H NMR of **2d**

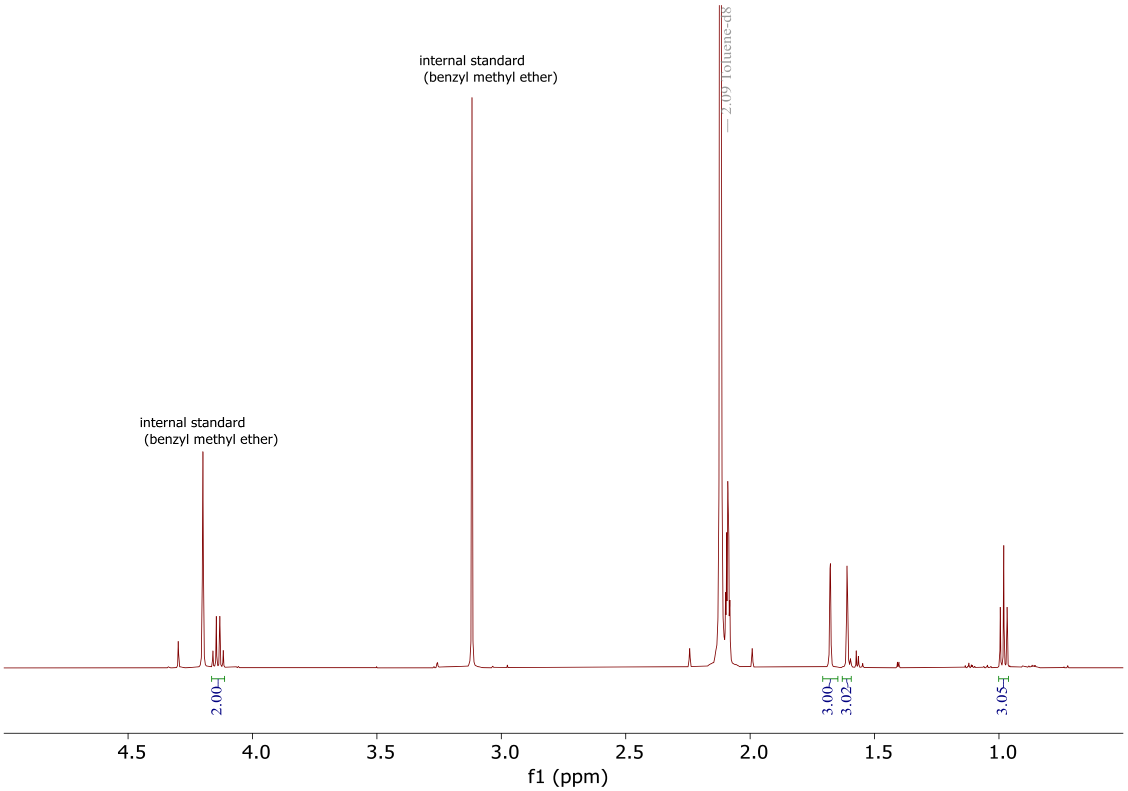

internal standard
(benzyl methyl ether)
internal standard
(benzyl methyl ether)
2.09 Toluene-d8
2.00
3.00
3.02
3.05
4.5
4.0
3.5
3.0
2.5
2.0
1.5
1.0
f1 (ppm)

^{1}H NMR of **2e**

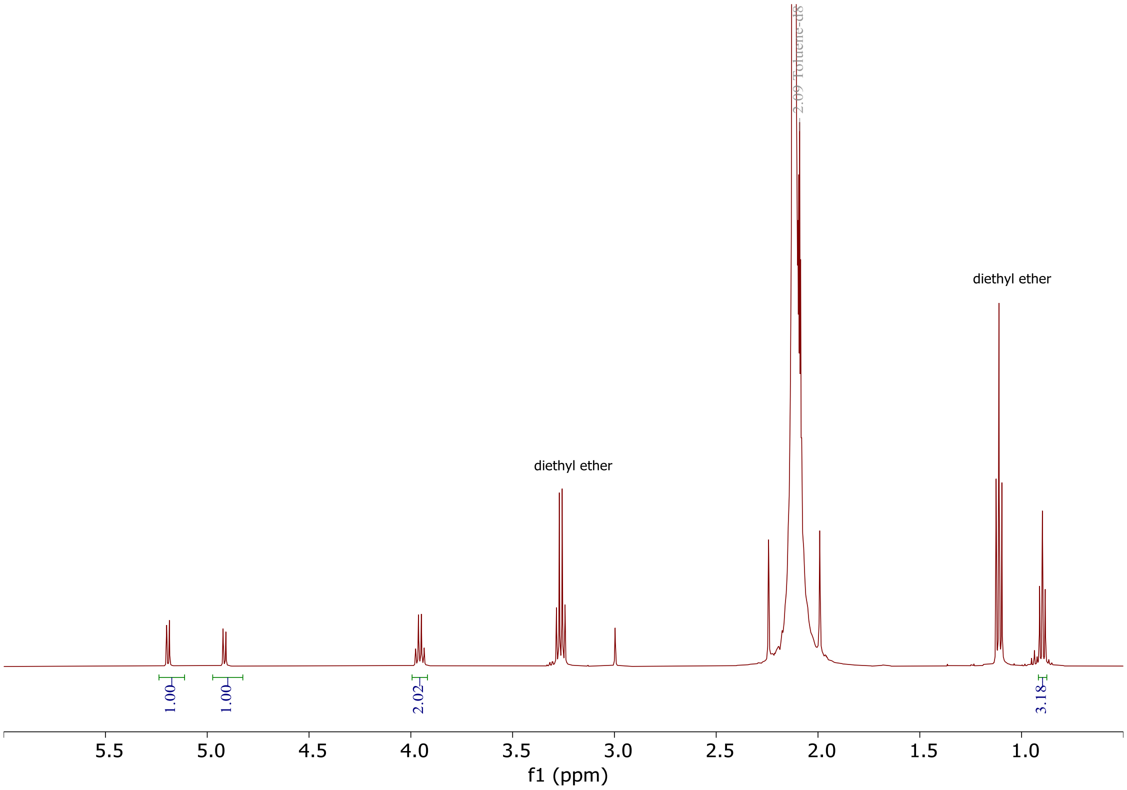

2.09 Toluene-d8
diethyl ether
diethyl ether
1.00
1.00
2.02
3.18
5.5
5.0
4.5
4.0
3.5
3.0
2.5
2.0
1.5
1.0
f1 (ppm)

^{1}H NMR of **2j**

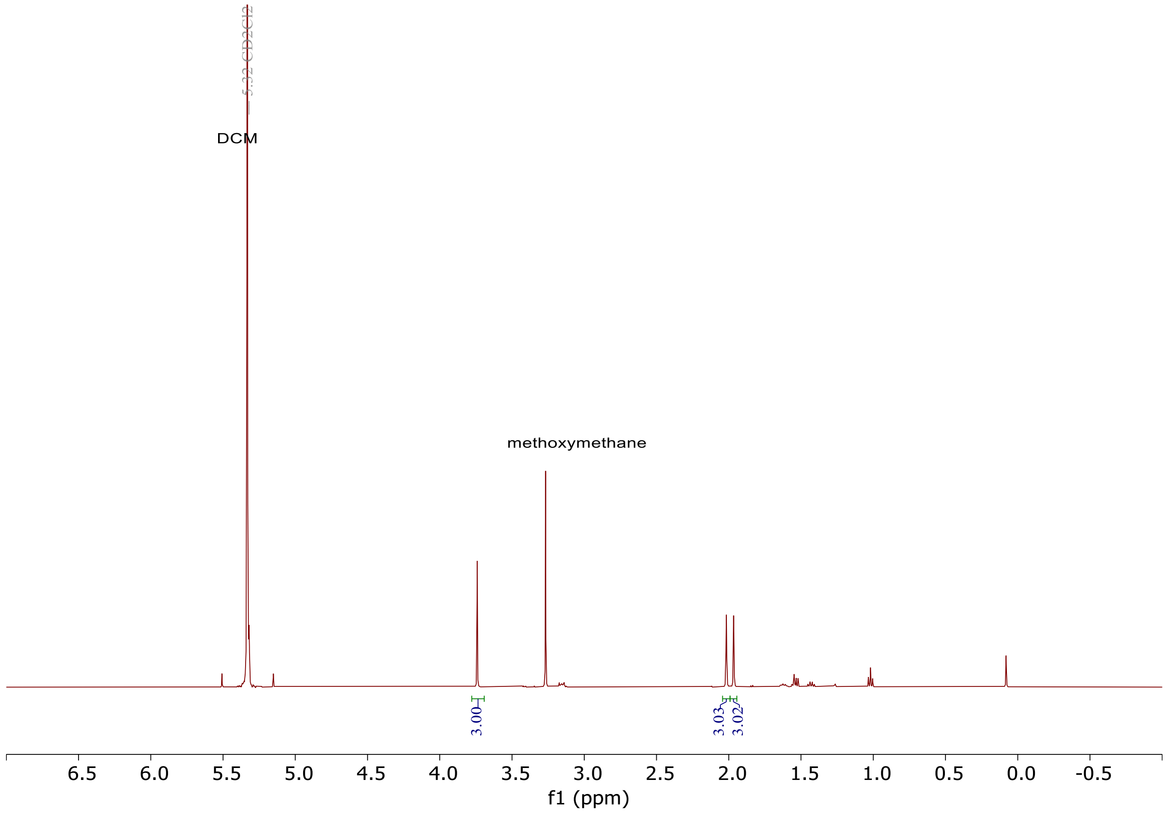

5.32 CD2Cl2
DCM
methoxymethane
3.00
3.03
3.02
6.5
6.0
5.5
5.0
4.5
4.0
3.5
3.0
2.5
2.0
1.5
1.0
0.5
0.0
-0.5
f1 (ppm)

^{1}H NMR and ^{13}C NMR of (*S*)-**1d**

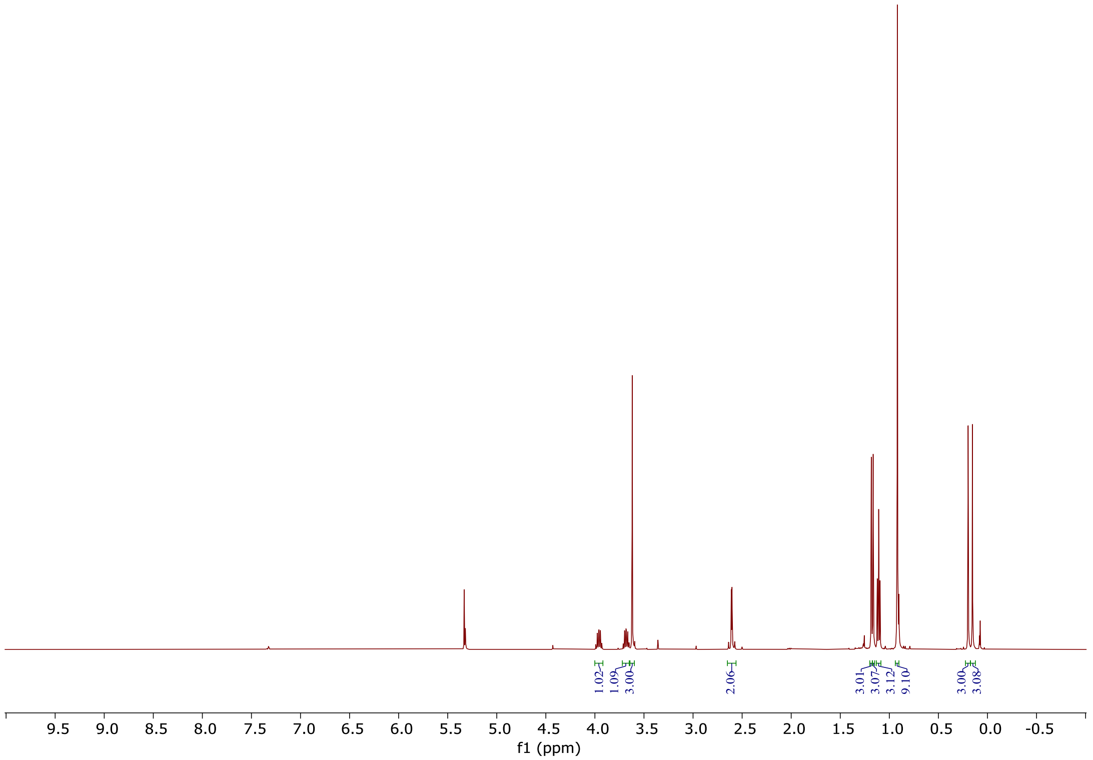

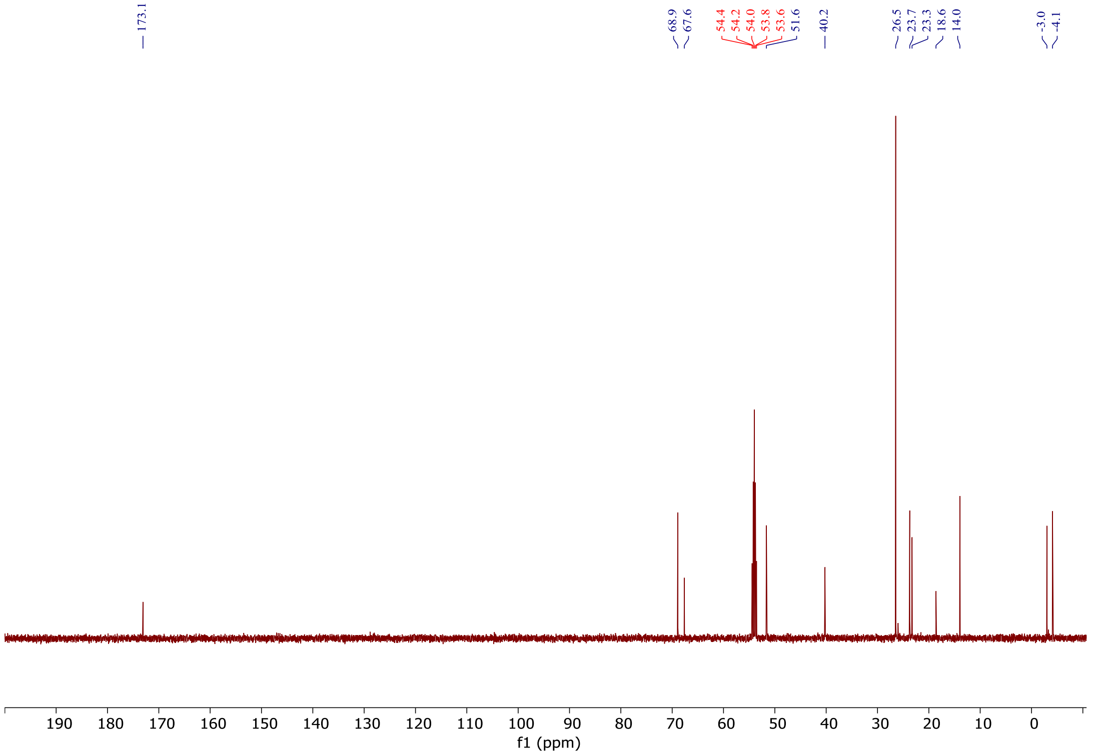

^{1}H NMR of (*S*)-**1e**

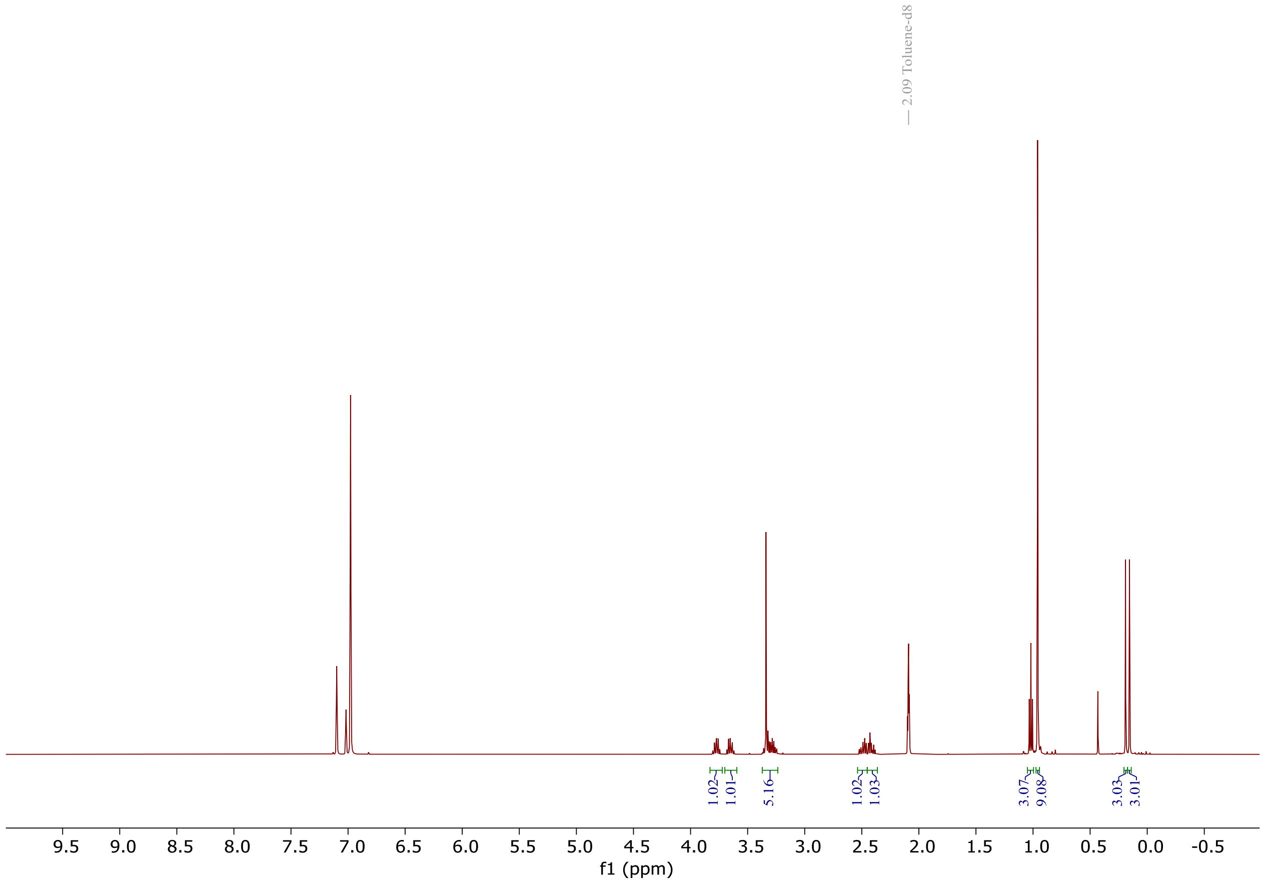

2.09 Toluene-d8
1.02
1.01
5.16
1.02
1.03
3.07
9.08
3.03
3.01
9.5 9.0 8.5 8.0 7.5 7.0 6.5 6.0 5.5 5.0 4.5 4.0 3.5 3.0 2.5 2.0 1.5 1.0 0.5 0.0 -0.5
f1 (ppm)

^{1}H NMR and ^{13}C NMR of (*S*)-**1f**

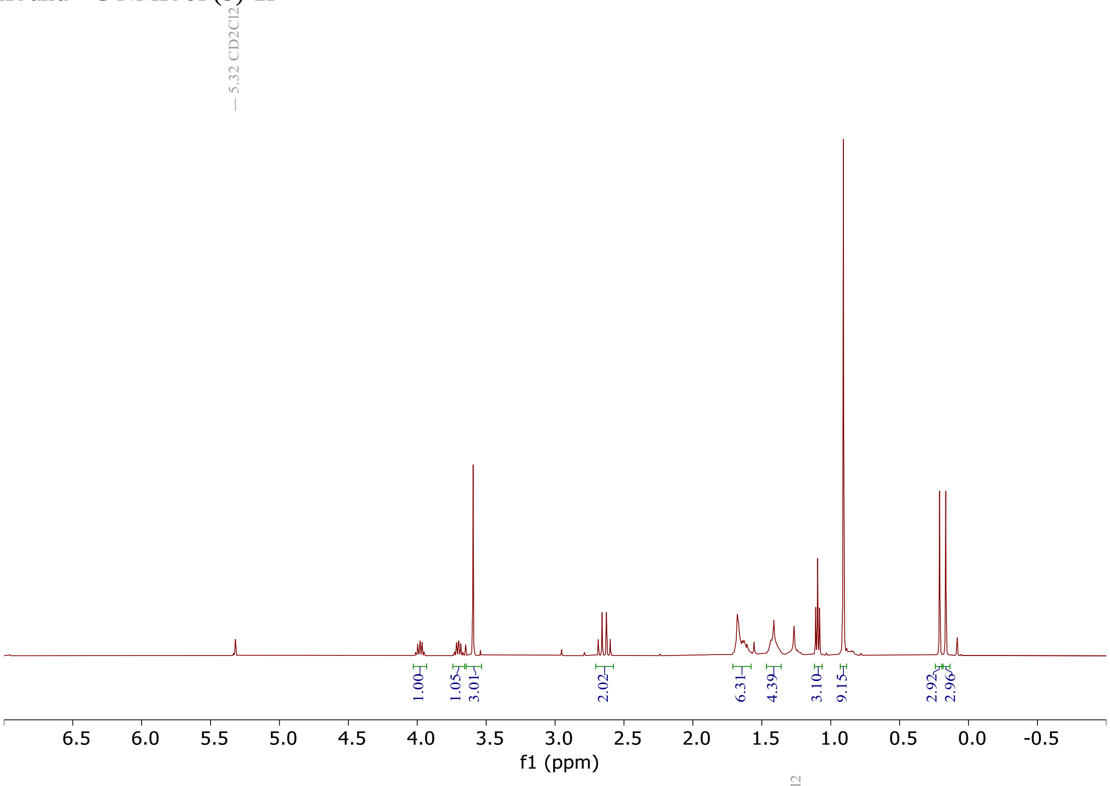

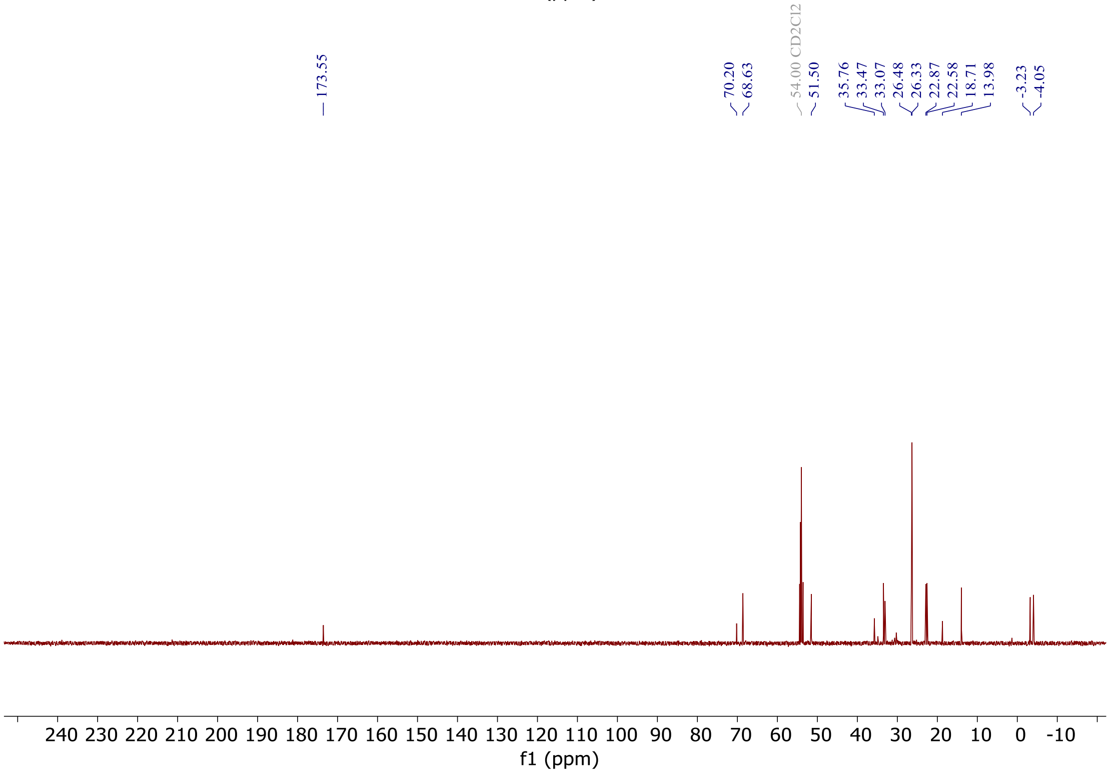

^{1}H NMR and ^{13}C NMR of (*S*)-**1g**

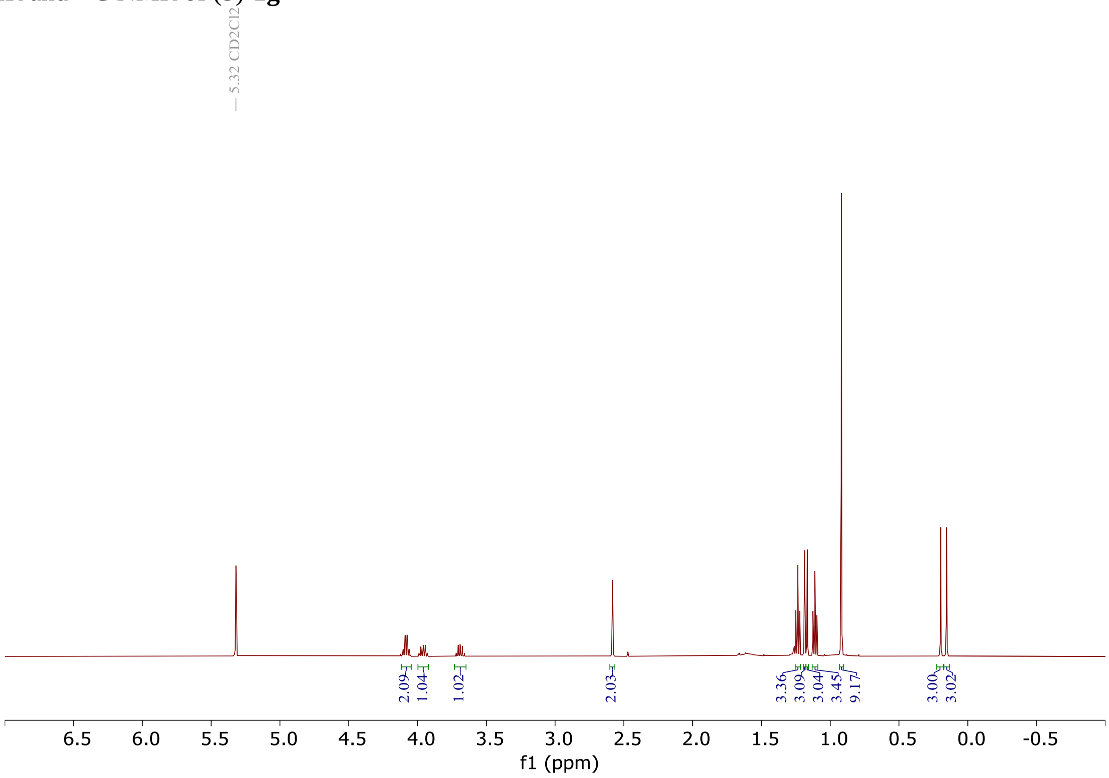

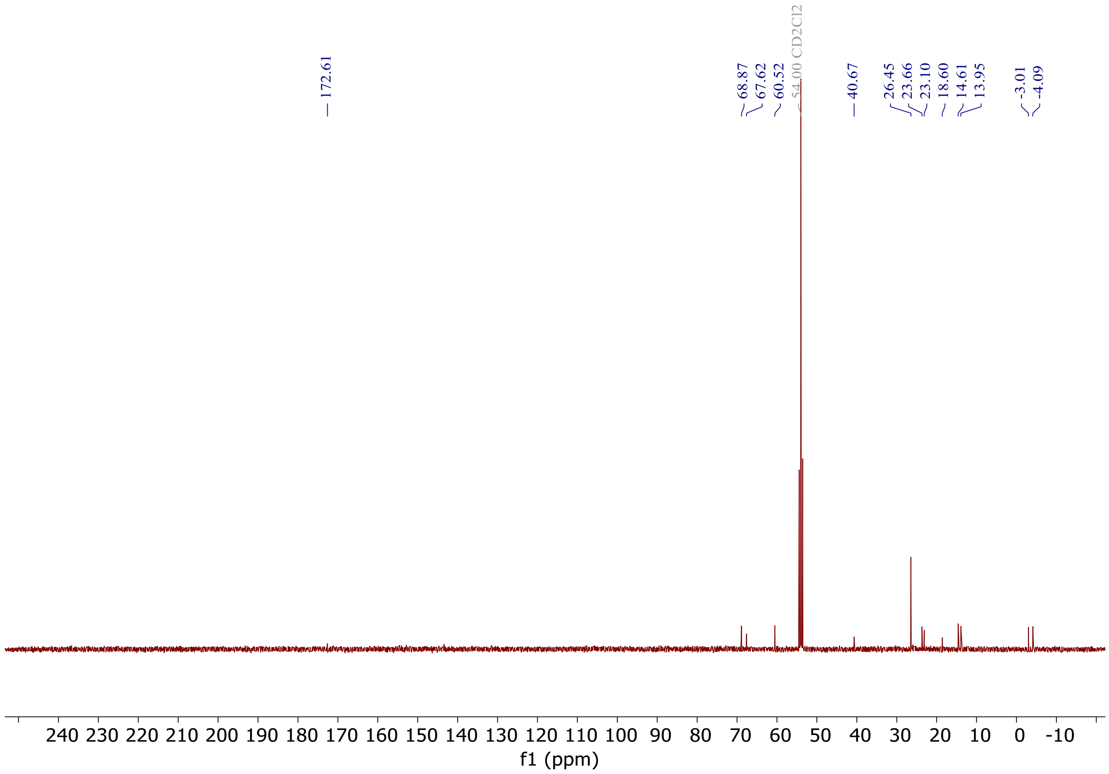

^{1}H NMR and ^{13}C NMR of (*S*)-**1h**

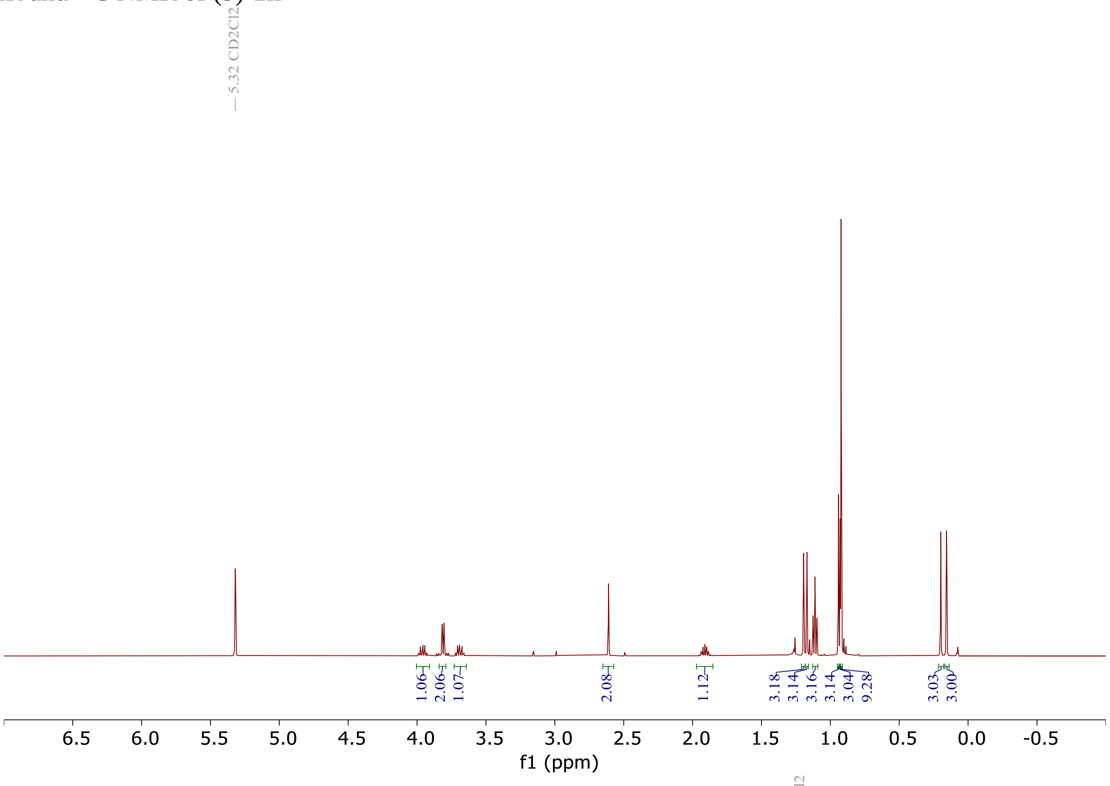

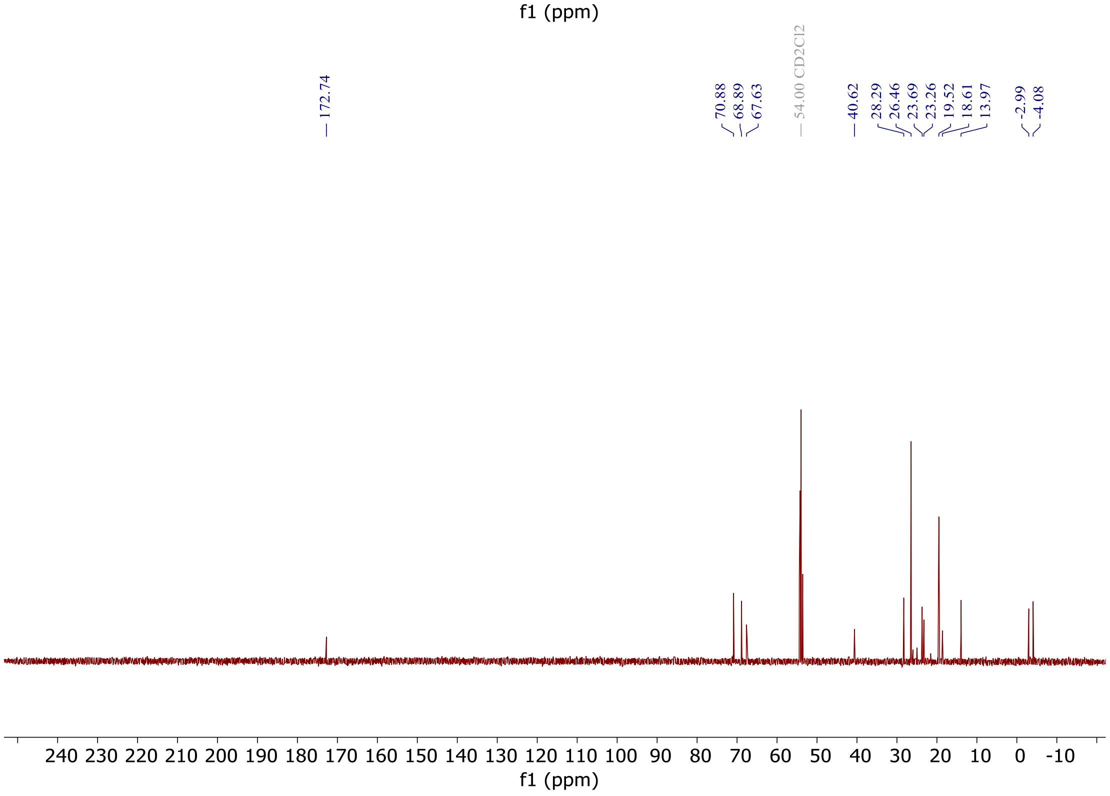

^{1}H NMR and ^{13}C NMR of (*S*)-**1i**

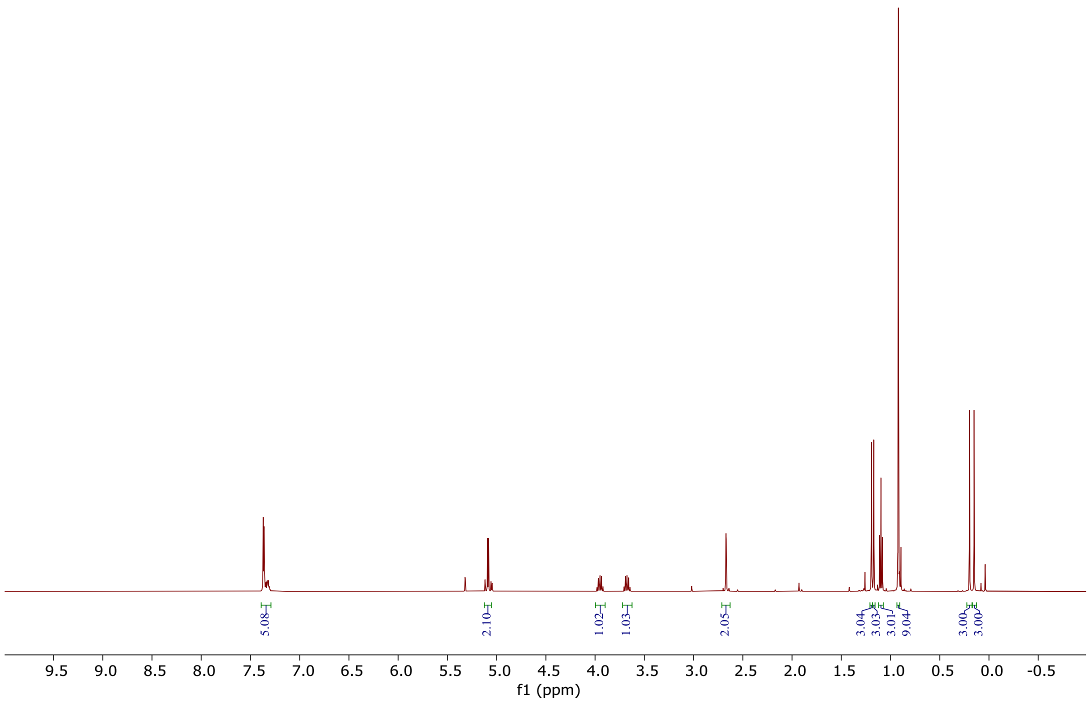

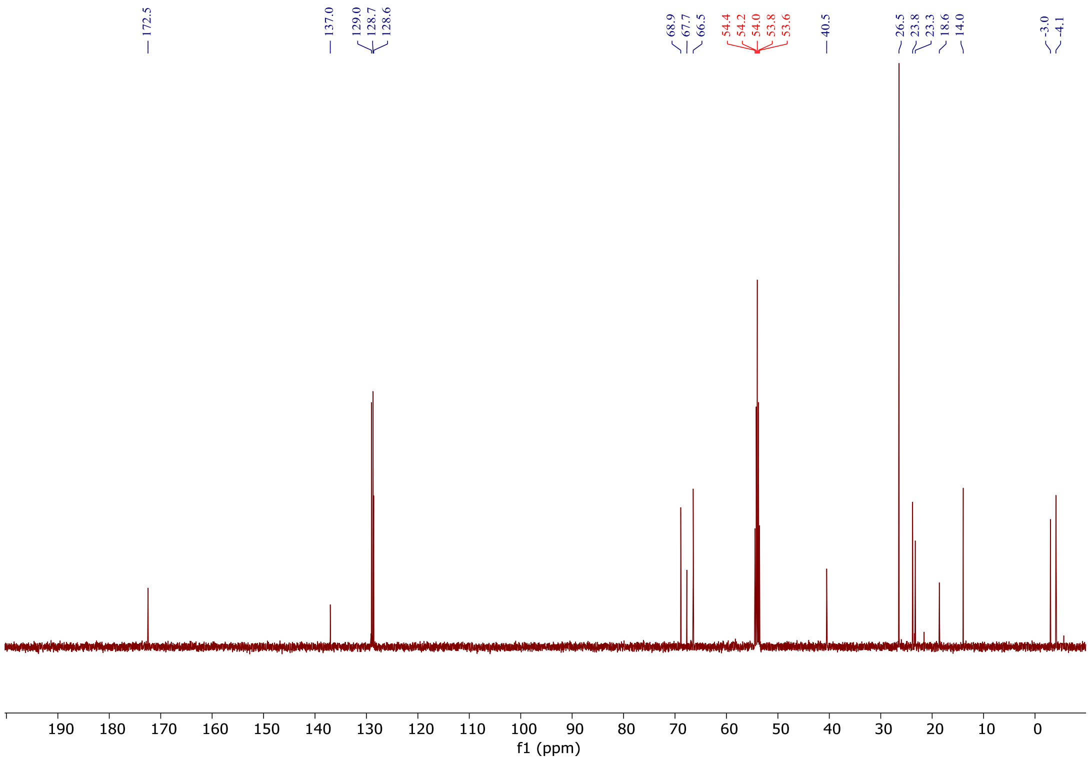

^{1}H NMR and ^{13}C NMR of (*S*)-**1j**

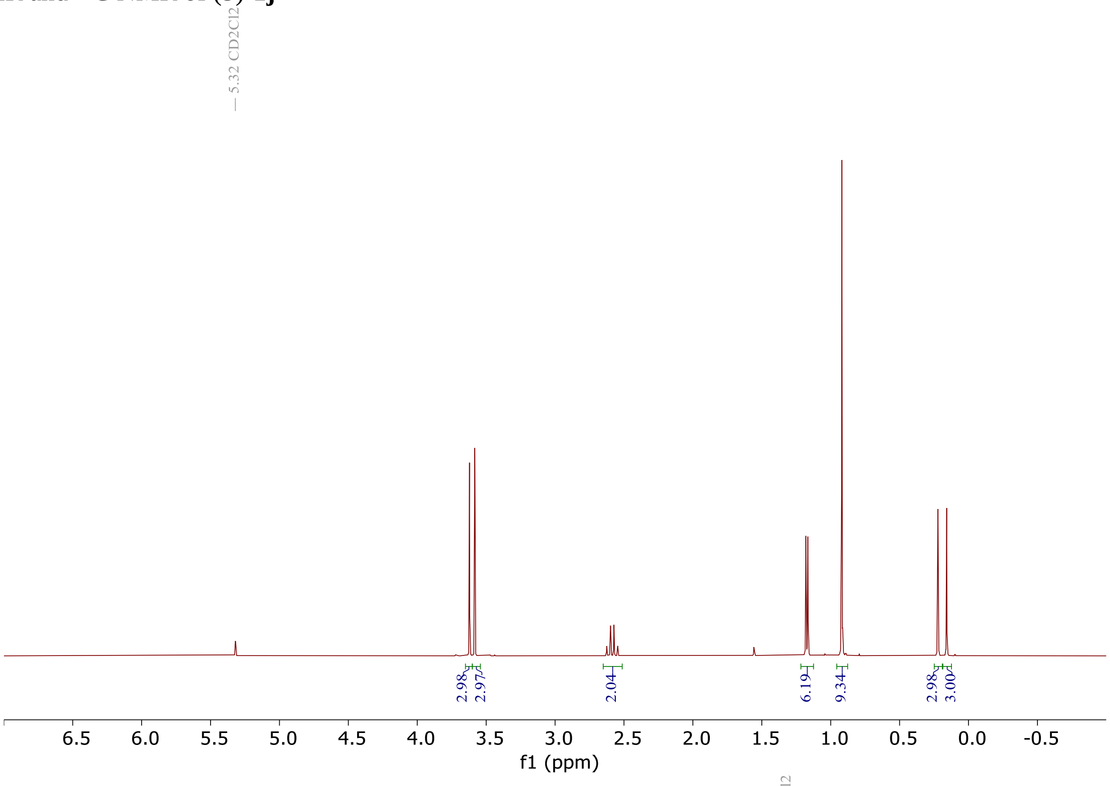

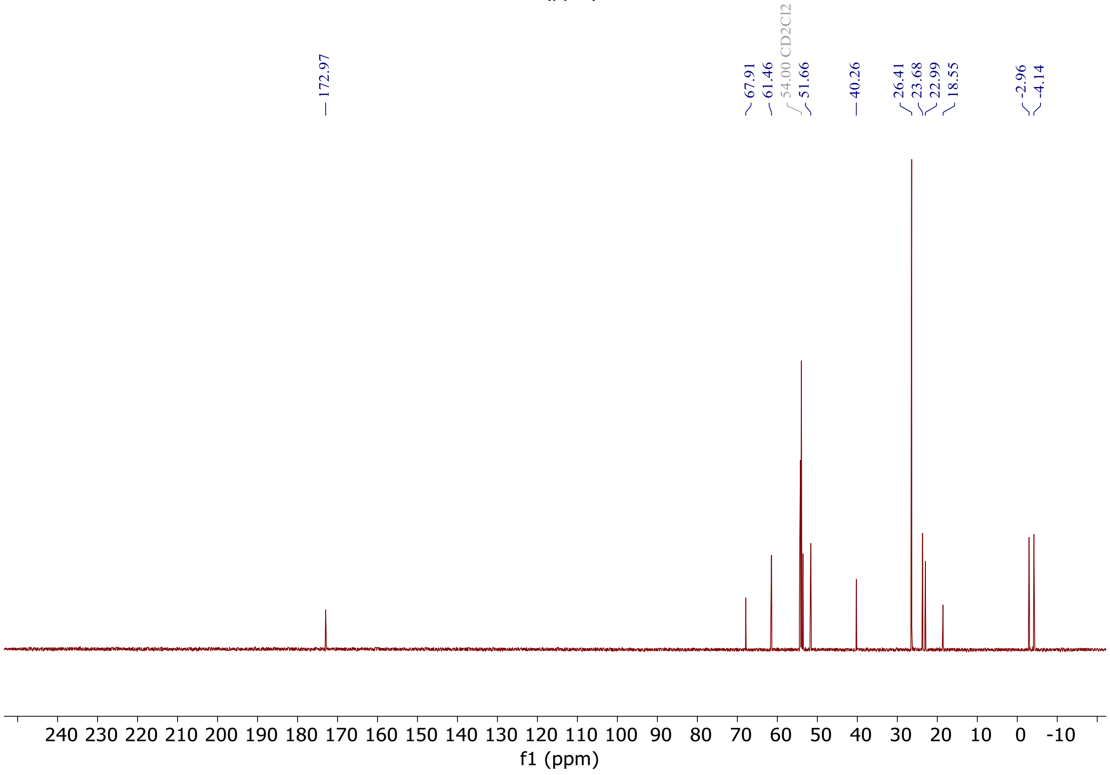

^{1}H NMR and ^{13}C NMR of (*S*)-**1k**

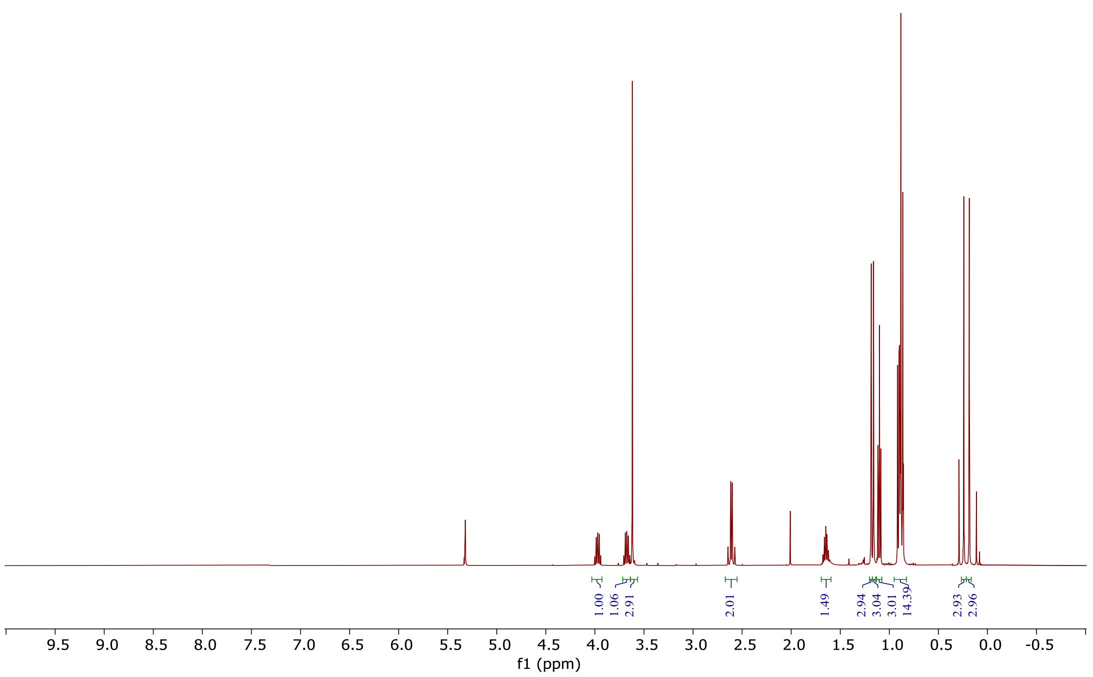

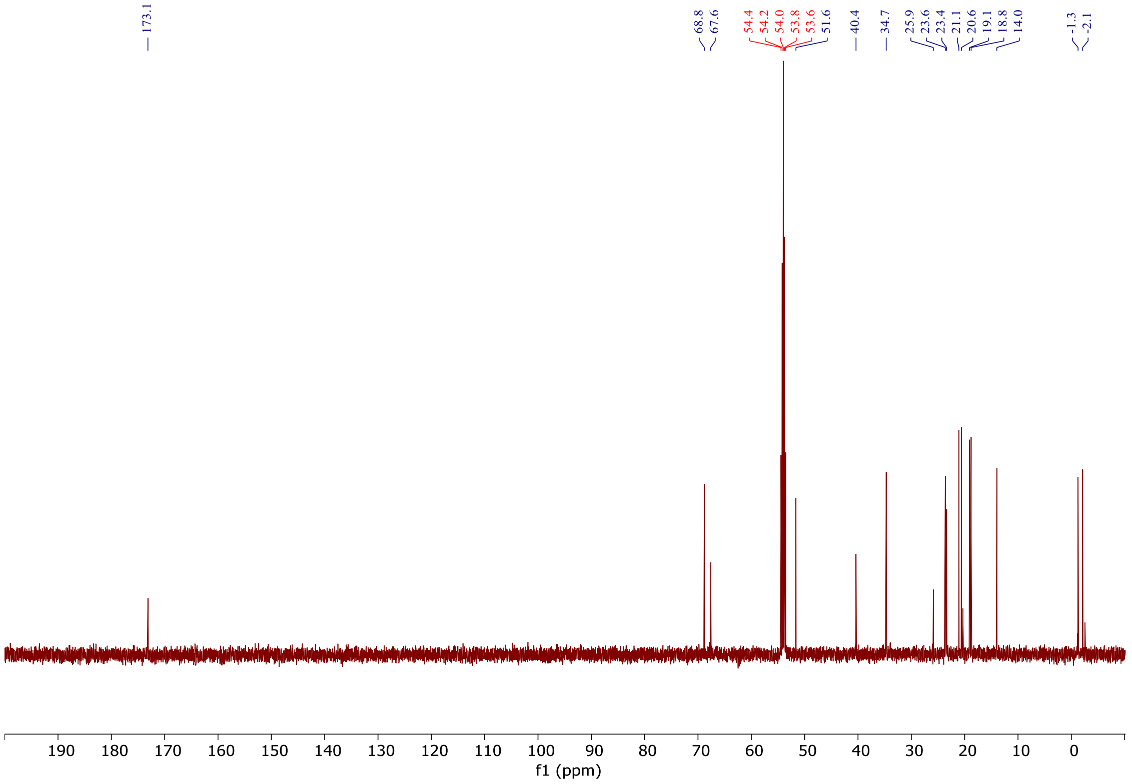

^{1}H NMR and ^{13}C NMR of (*S*)-**1l**

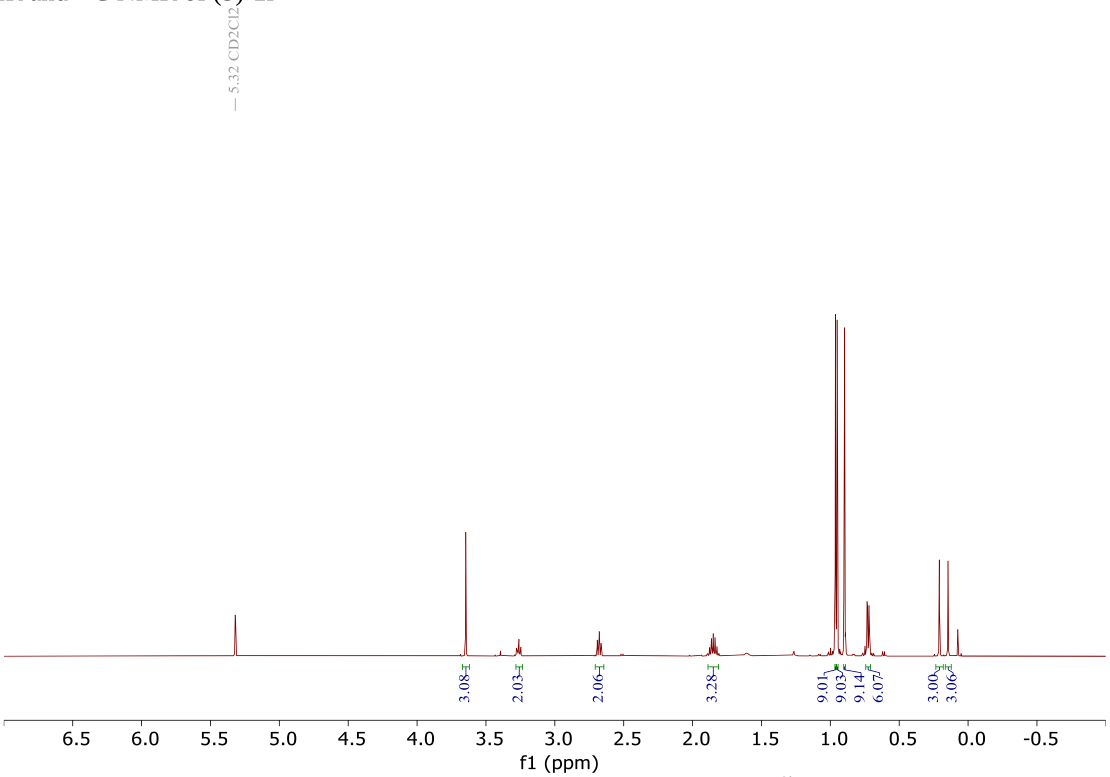

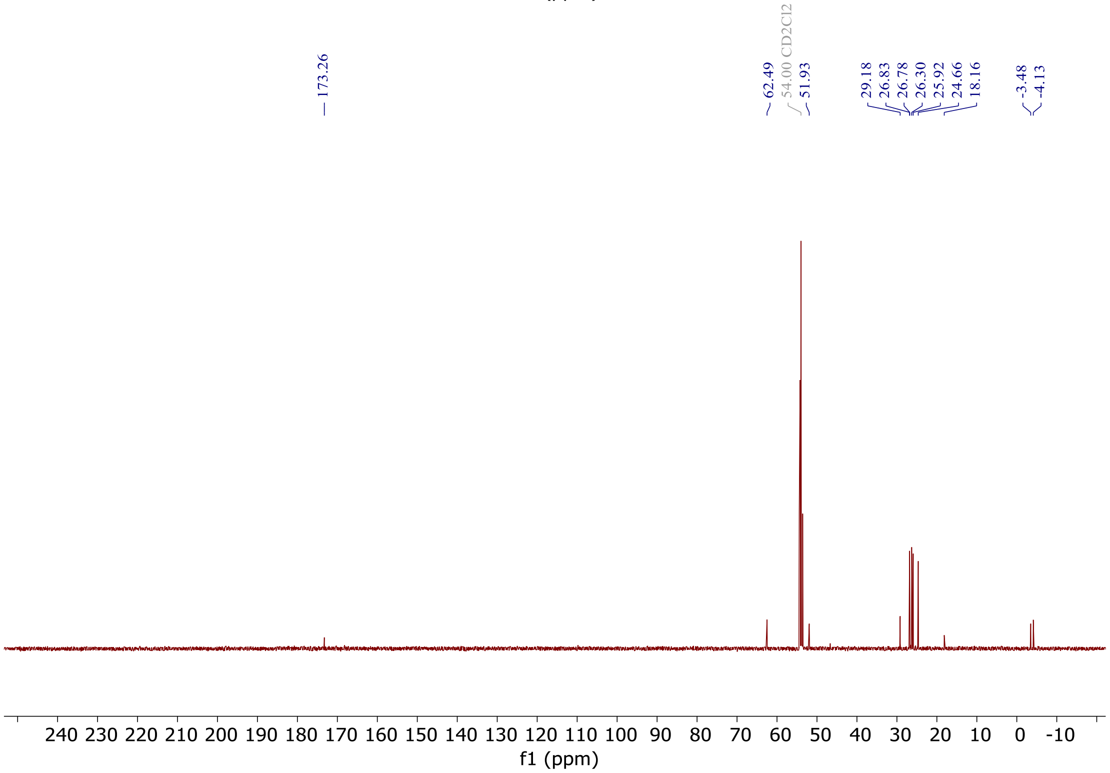

^{1}H NMR and ^{13}C NMR of (*R*)-**1m**

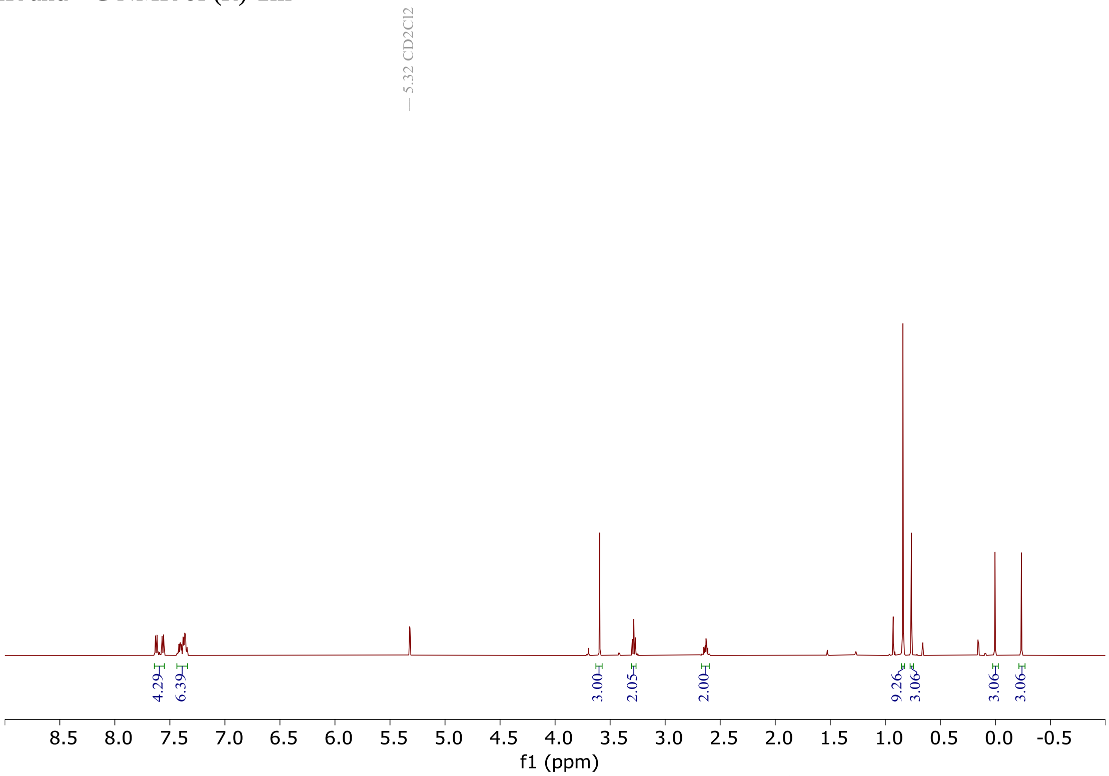

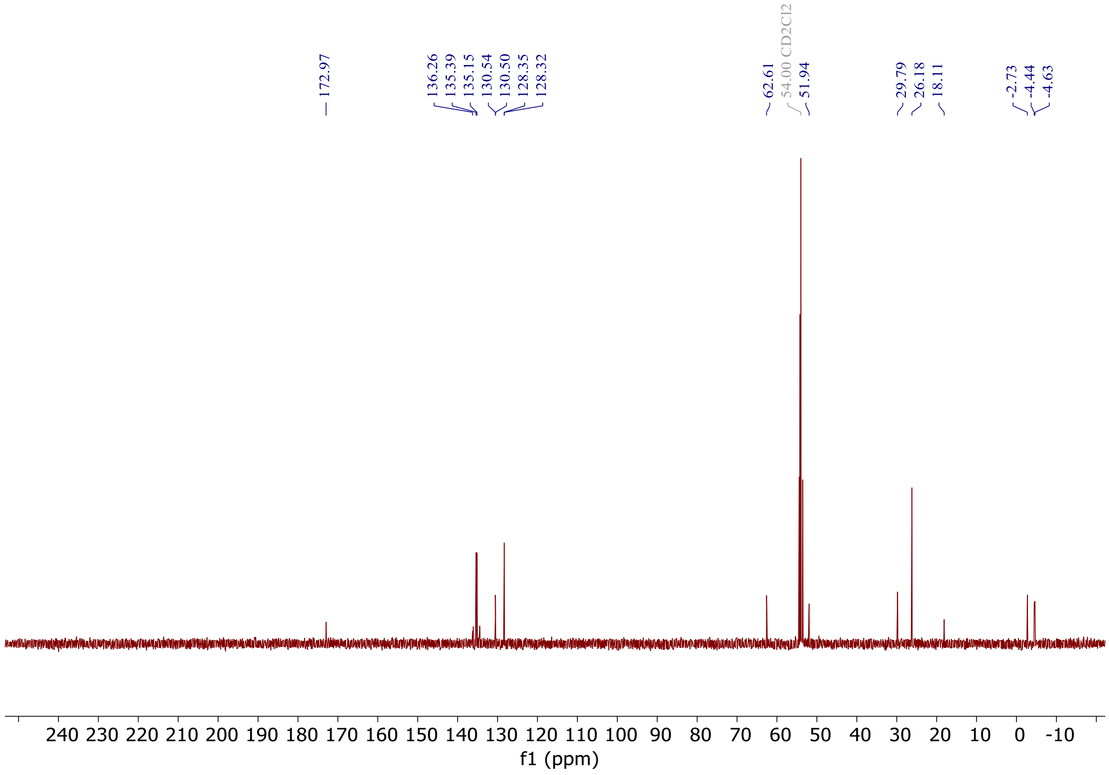

^{1}H NMR and ^{13}C NMR of *cis*-**1n**

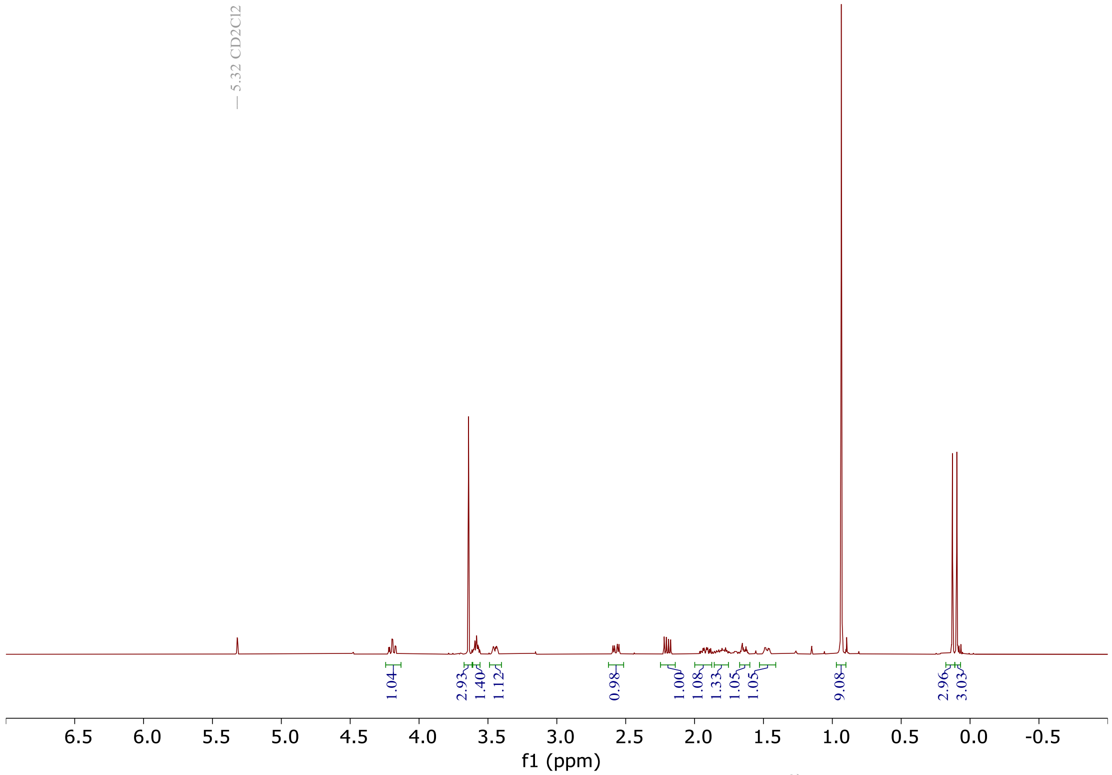

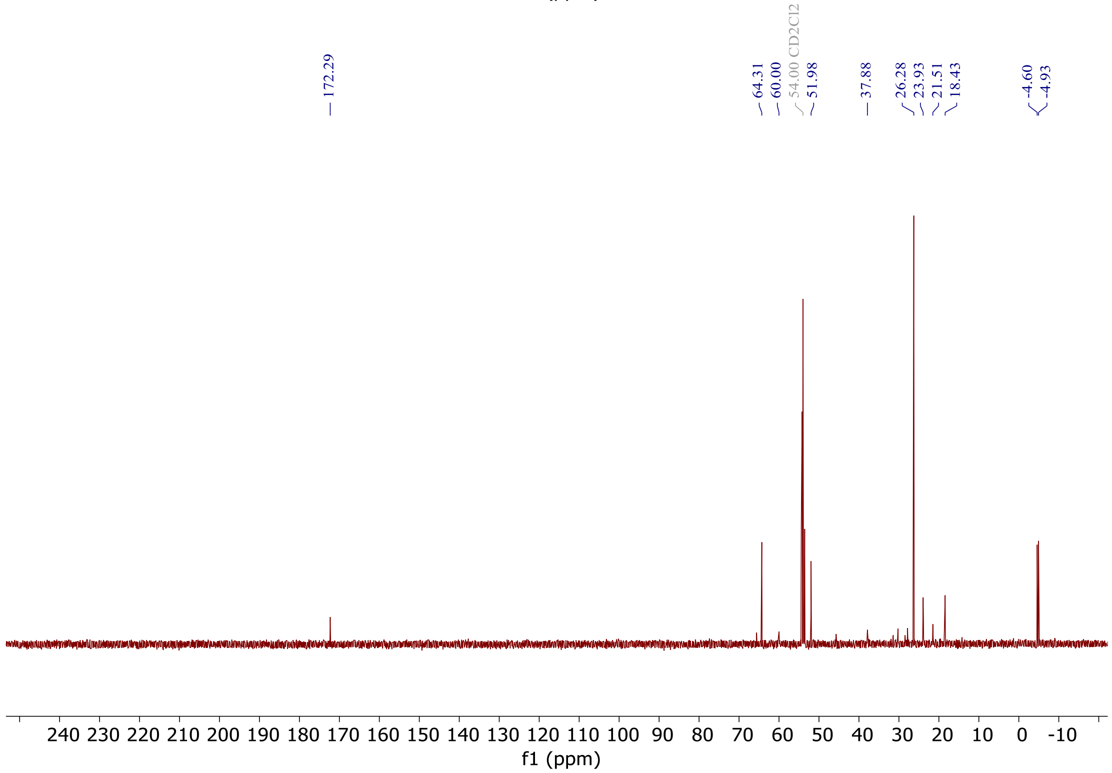

NOESY of *cis*-**1n**

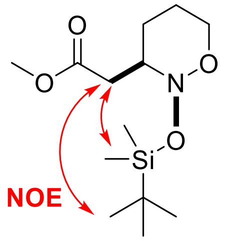

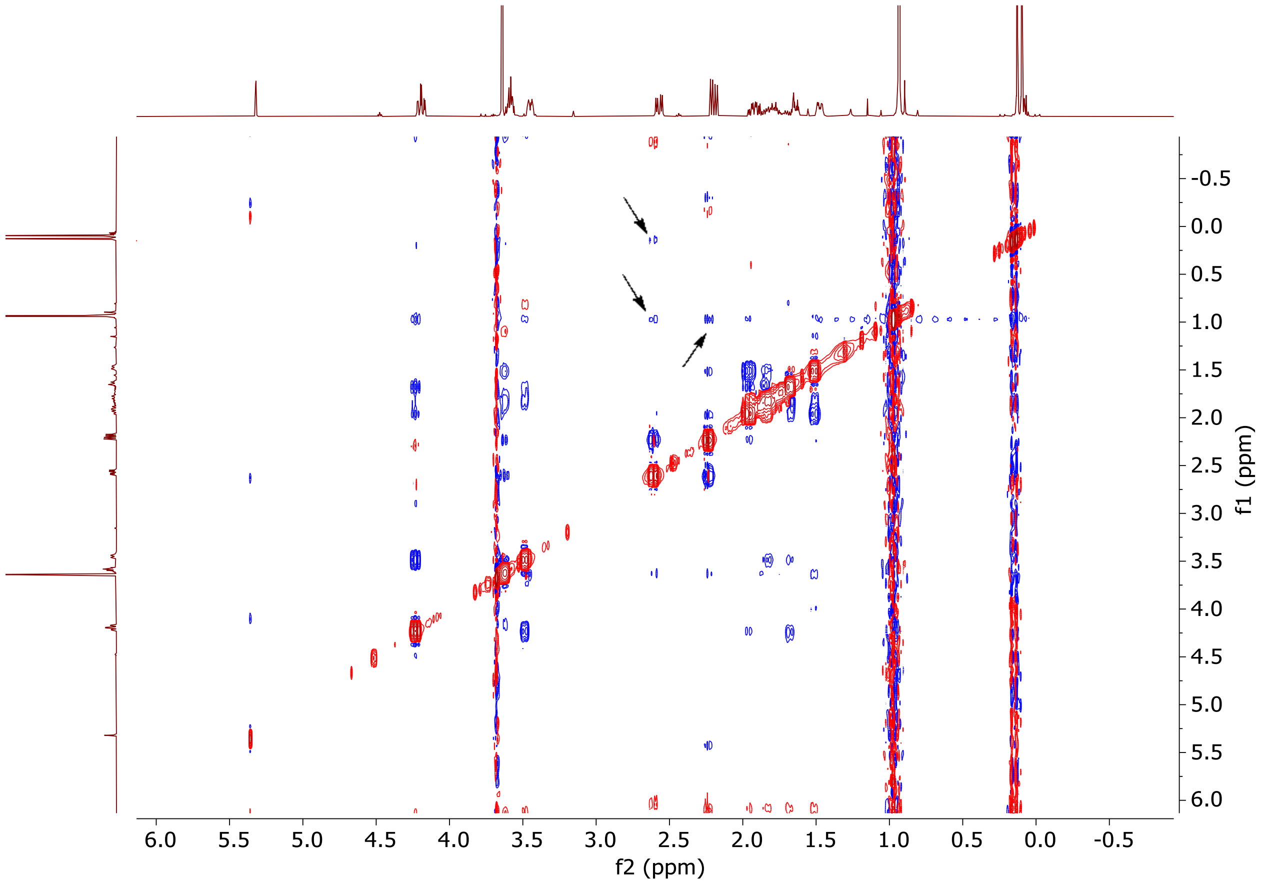

4 HPLC Chromatograms

HPLC traces of *rac*-**1d**

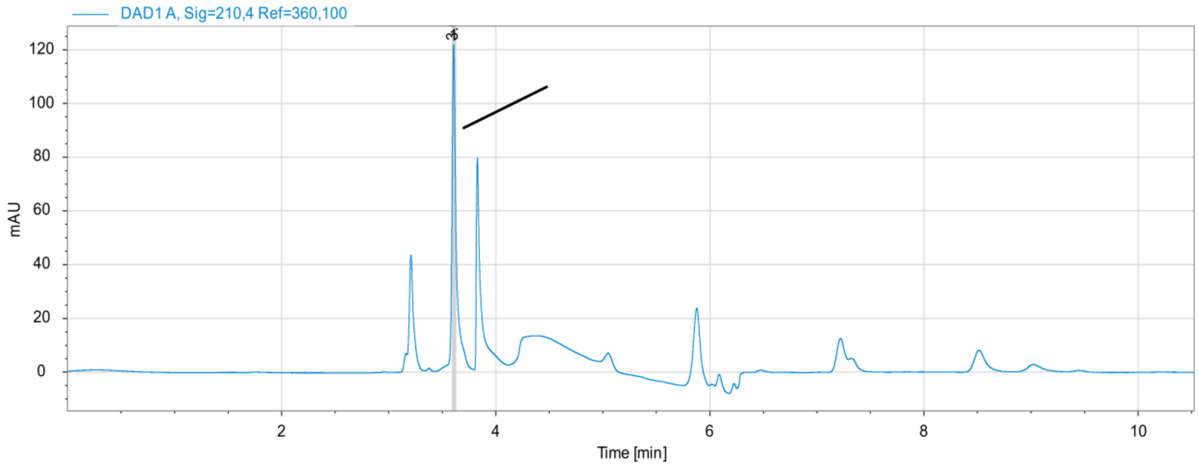

Peak	Retention time (min)
1	3.59

HPLC traces of (*S*)-**1d**

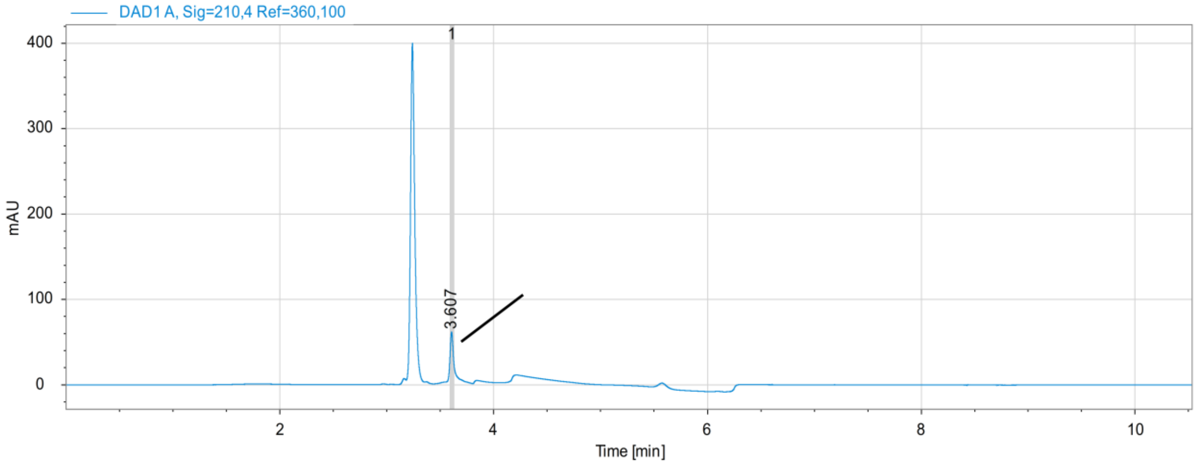

Peak	Retention time (min)
1	3.60

HPLC traces of *rac*-**1d**

HPLC traces of *rac*-**1d**

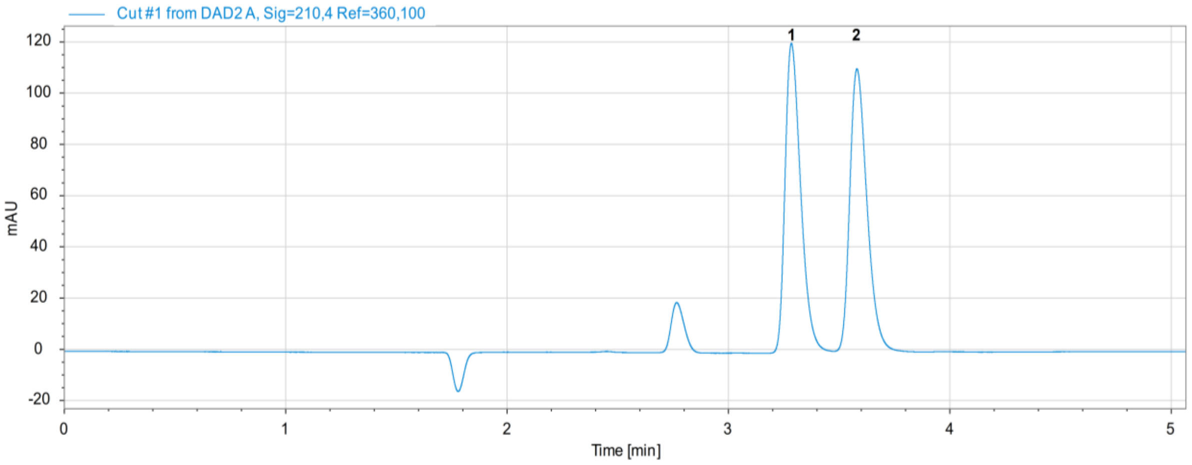

Peak	Retention time (min)	Area (%)
1	3.28	49.8
2	3.58	50.2
Total		100

HPLC traces of (*S*)-**1d**

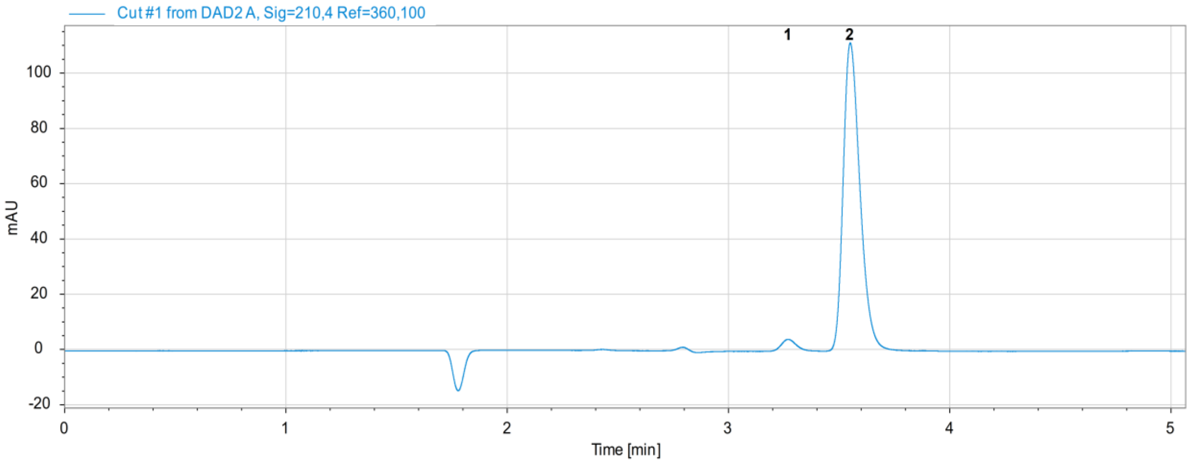

Peak	Retention time (min)	Area (%)
1	3.27	3.4
2	3.55	96.6
Total		100

HPLC traces of *rac*-**1e**

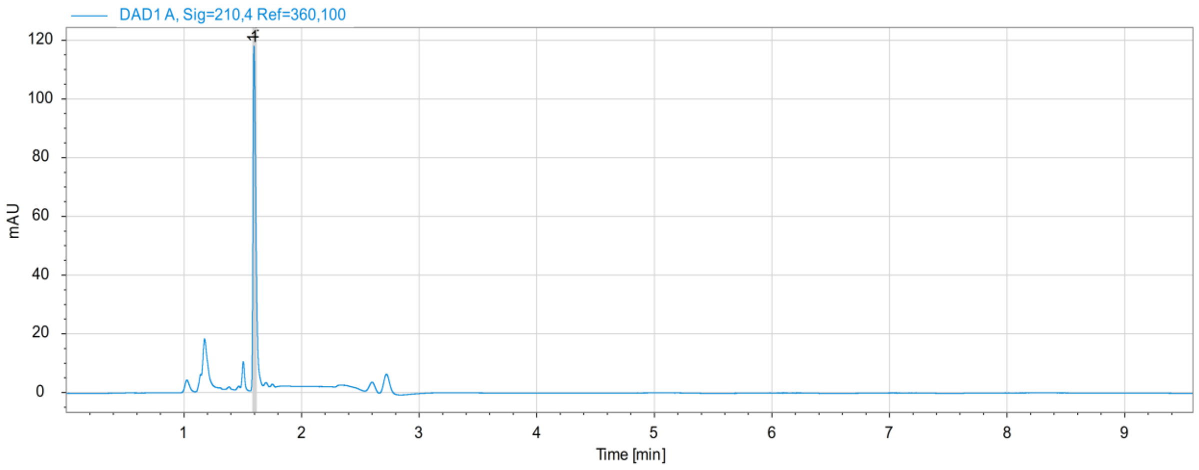

Peak	Retention time (min)
1	1.59

HPLC traces of (*S*)-**1e**

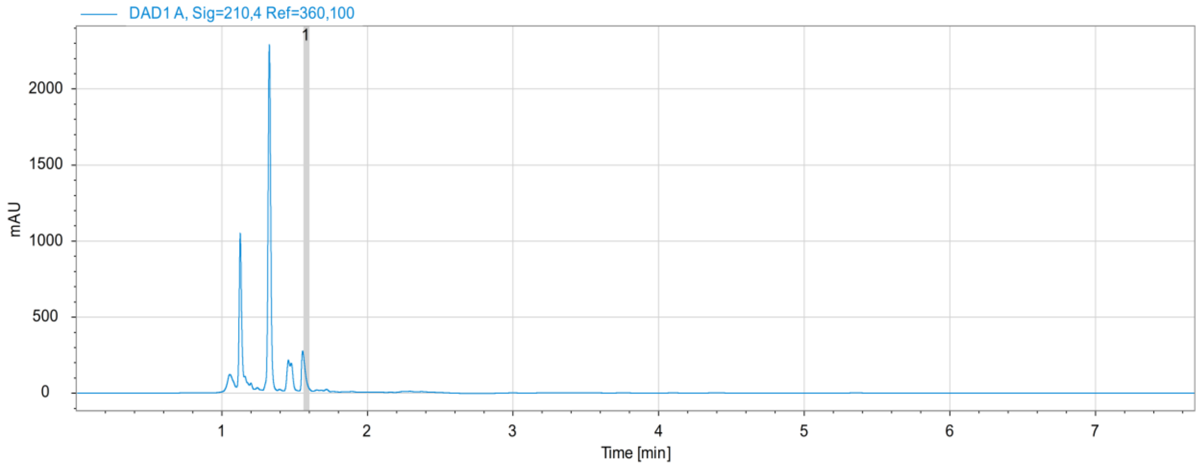

Peak	Retention time (min)
1	1.56

HPLC traces of *rac*-**1e**

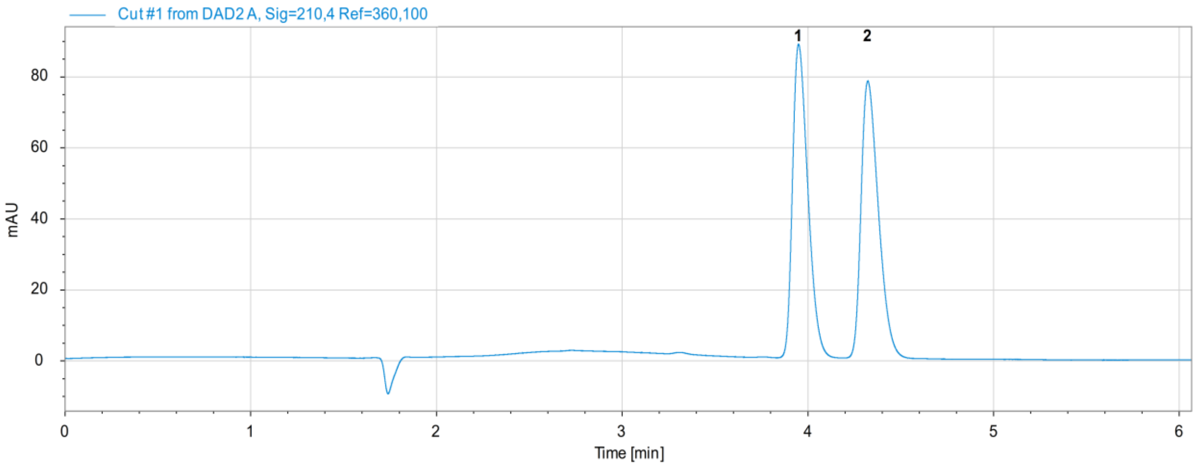

Peak	Retention time (min)	Area (%)
1	3.95	50.0
2	4.32	50.0
Total		100

HPLC traces of (*S*)-**1e**

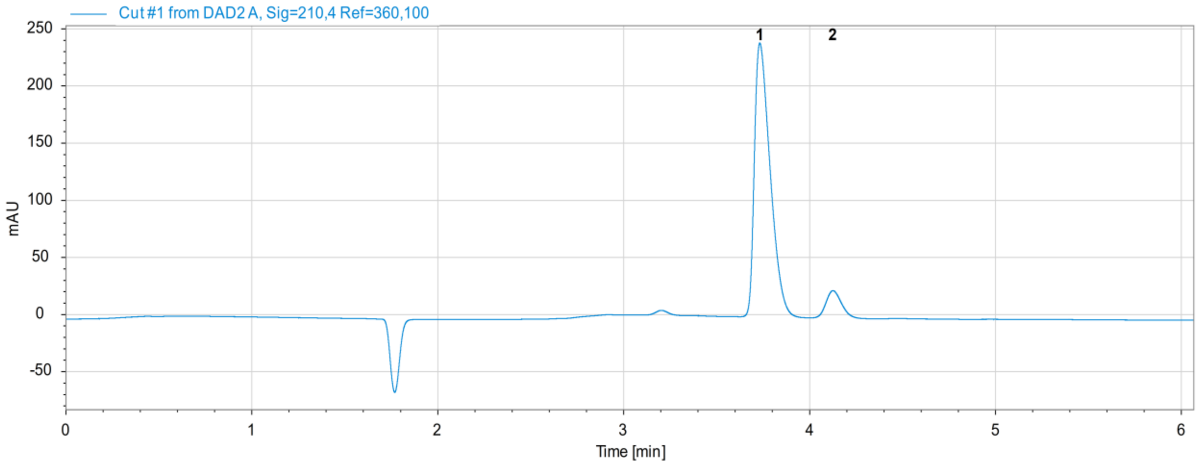

Peak	Retention time (min)	Area (%)
1	3.73	91.9
2	3.58	9.1
Total		100

HPLC traces of rac-**1f**

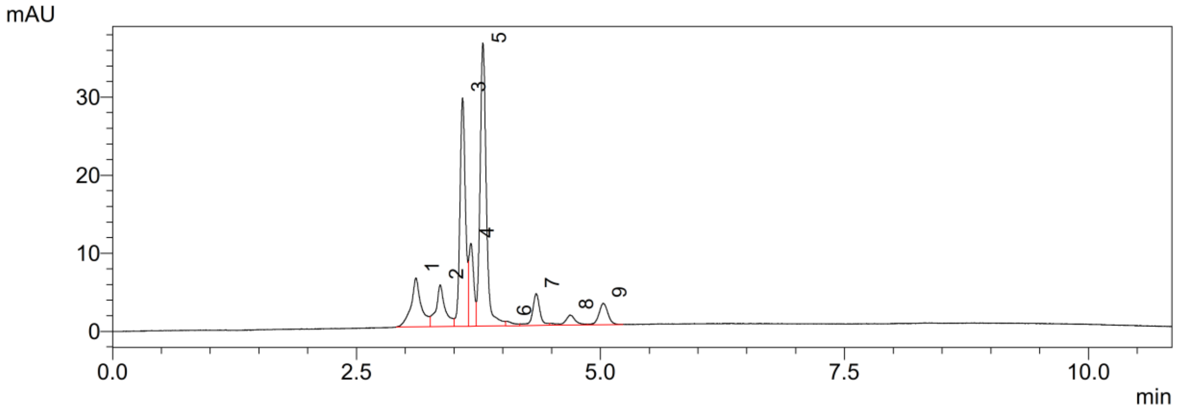

Peak	Retention time (min)
1	3.79

HPLC traces of (*S*)-**1f**

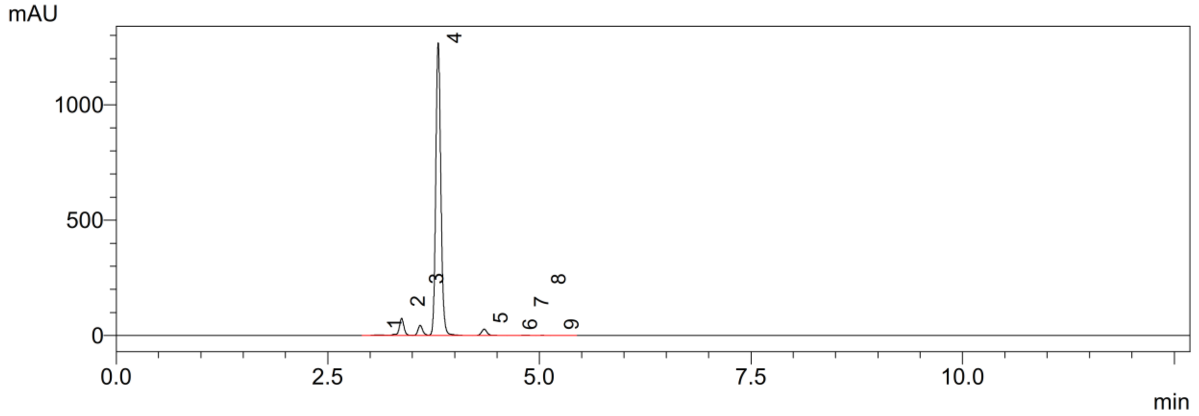

Peak	Retention time (min)
1	3.80

HPLC traces of rac-**1f**

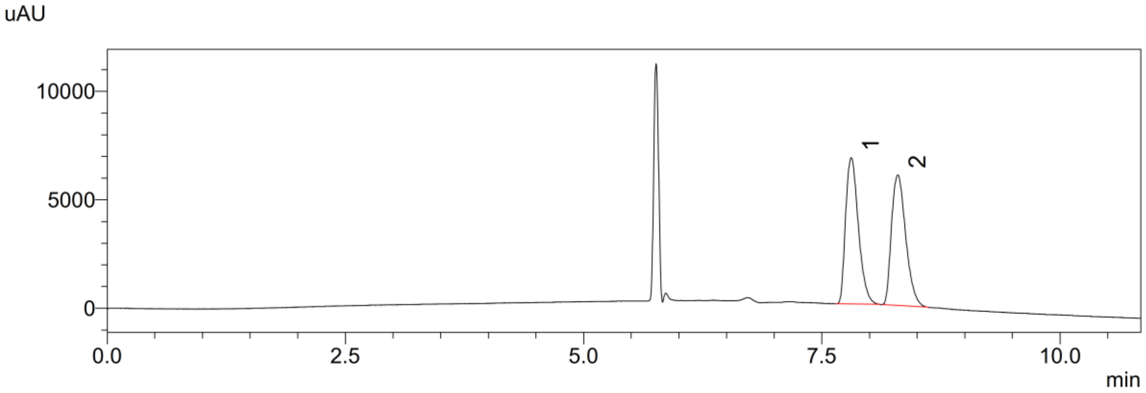

Peak	Retention time (min)	Area (%)
1	7.82	50
2	8.30	50
Total		100

HPLC traces of (*S*)-**1f**

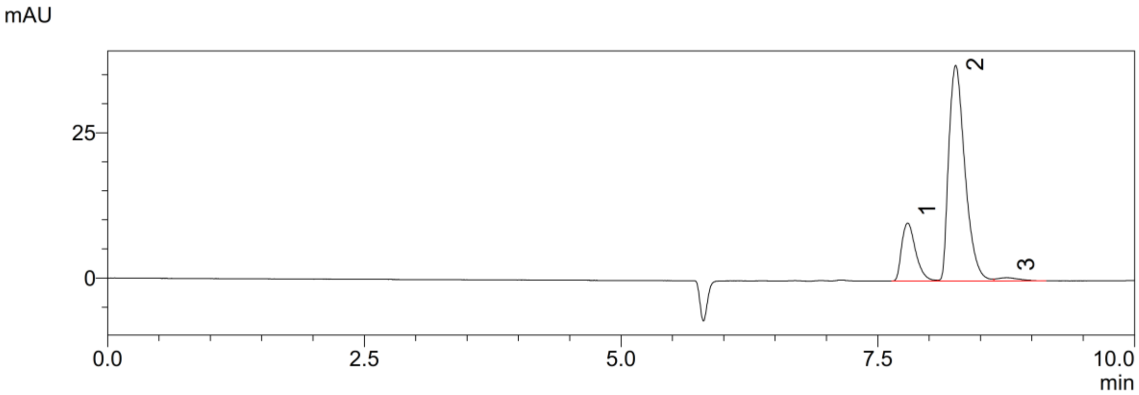

Peak	Retention time (min)	Area (%)
1	7.79	81.2
2	8.26	18.8
Total		100

HPLC traces of *rac*-**1g** and (*S*)-**1g**

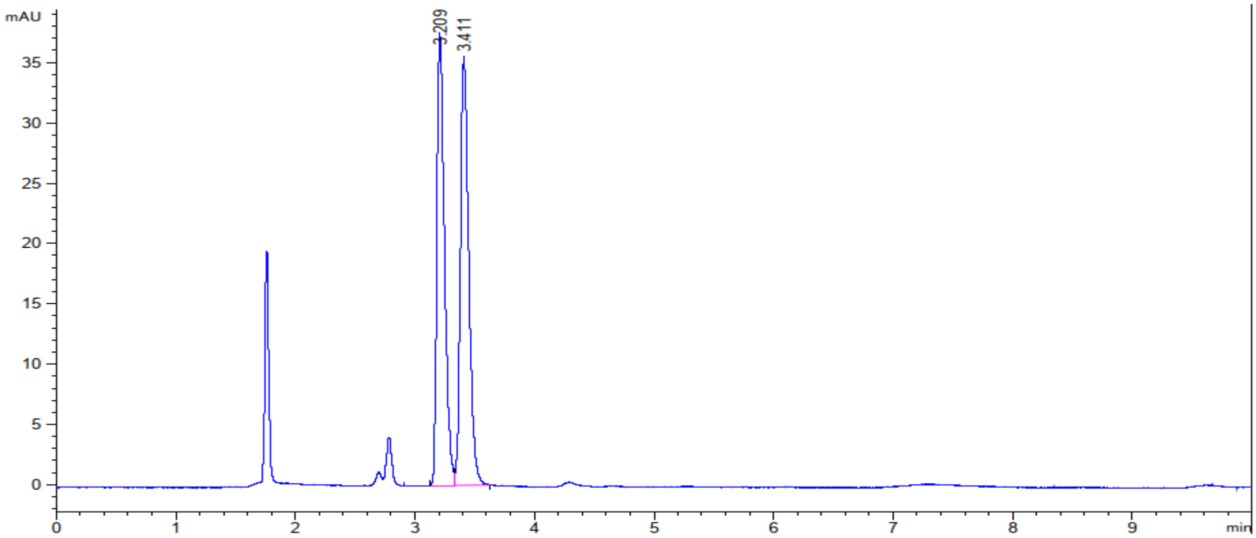

**Peak**	**Retention time (min)**	**Area (%)**
1	3.21	50
2	3.41	50
Total		100

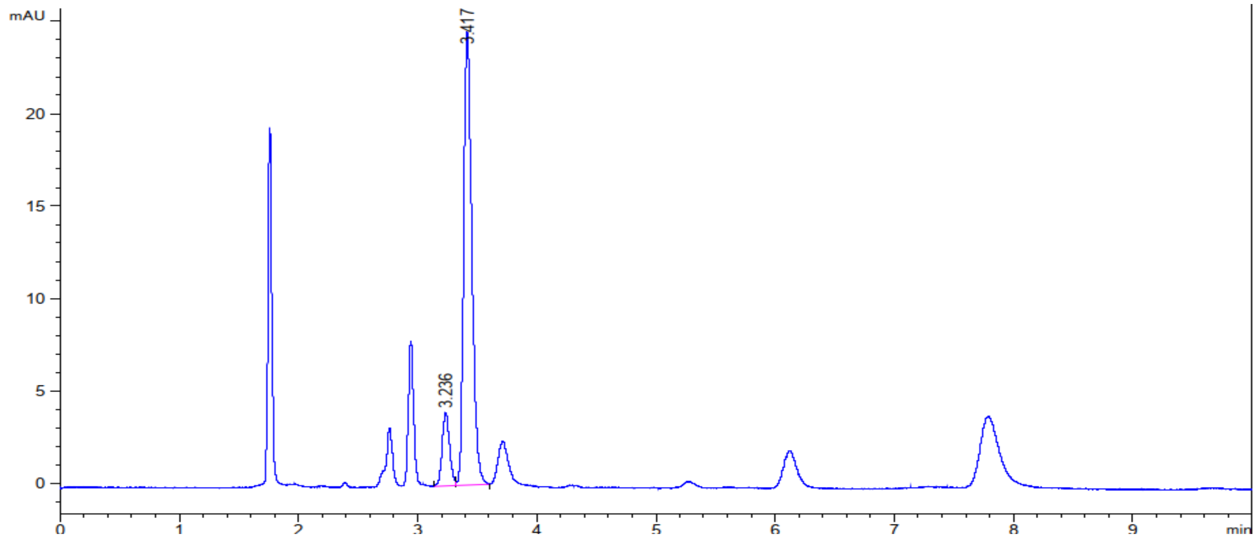

**Peak**	**Retention time (min)**	**Area (%)**
1	3.24	12.8
2	3.42	87.2
Total		100

HPLC traces of *rac*-**1h** and (*S*)-**1h**

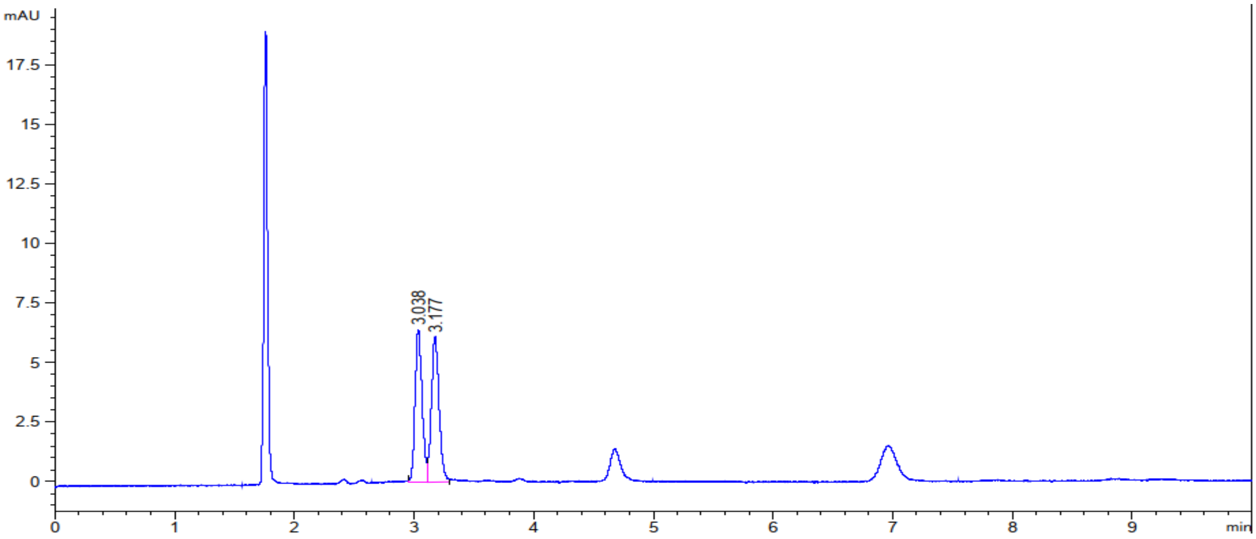

**Peak**	**Retention time (min)**	**Area (%)**
1	3.04	50
2	3.18	50
Total		100

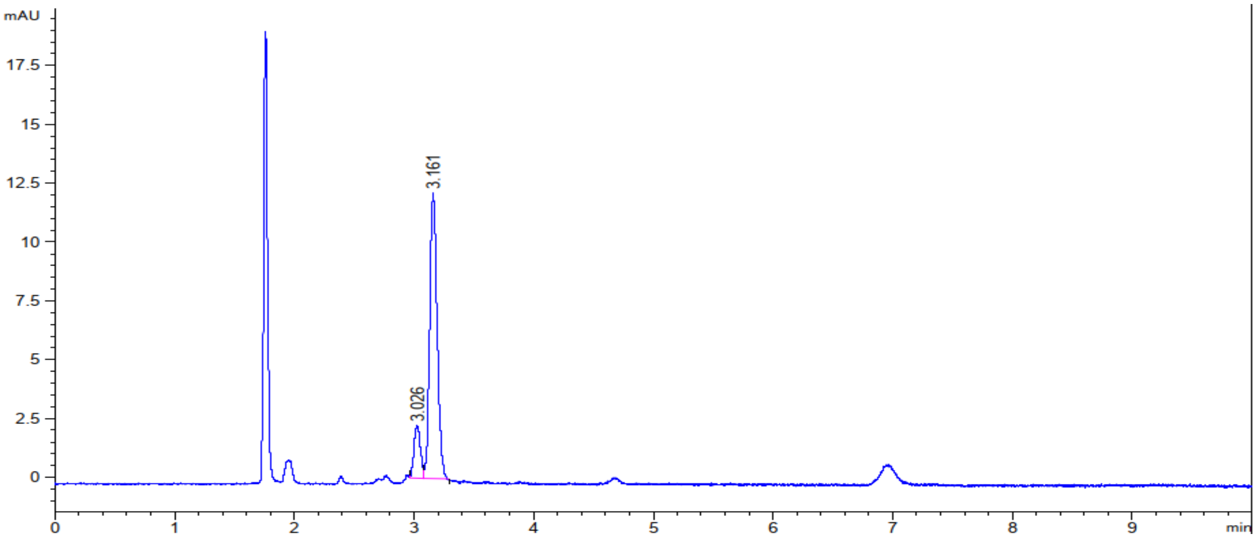

**Peak**	**Retention time (min)**	**Area (%)**
1	3.03	13.8
2	3.16	86.2
Total		100

HPLC traces of *rac*-**1i**

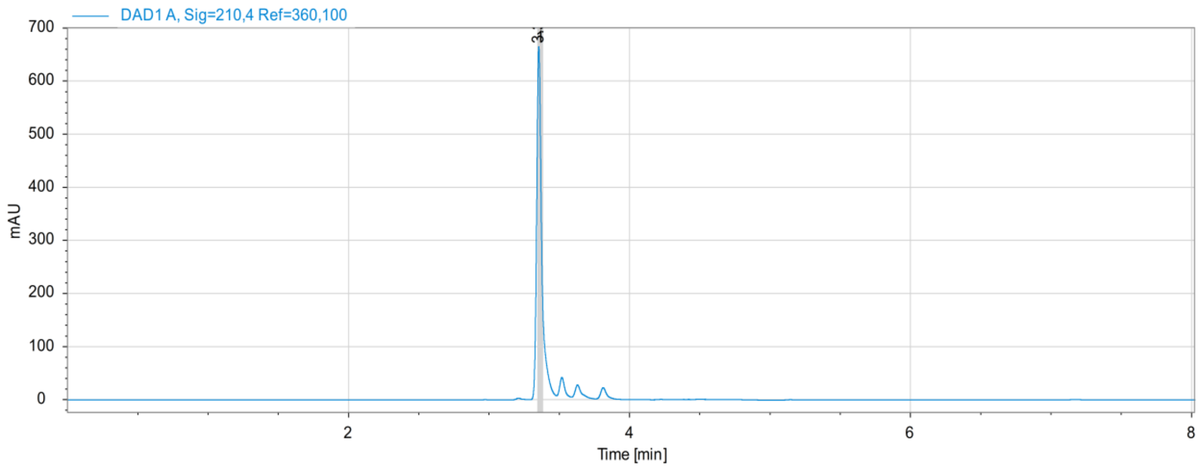

Peak	Retention time (min)
1	3.34

HPLC traces of (*S*)-**1i**

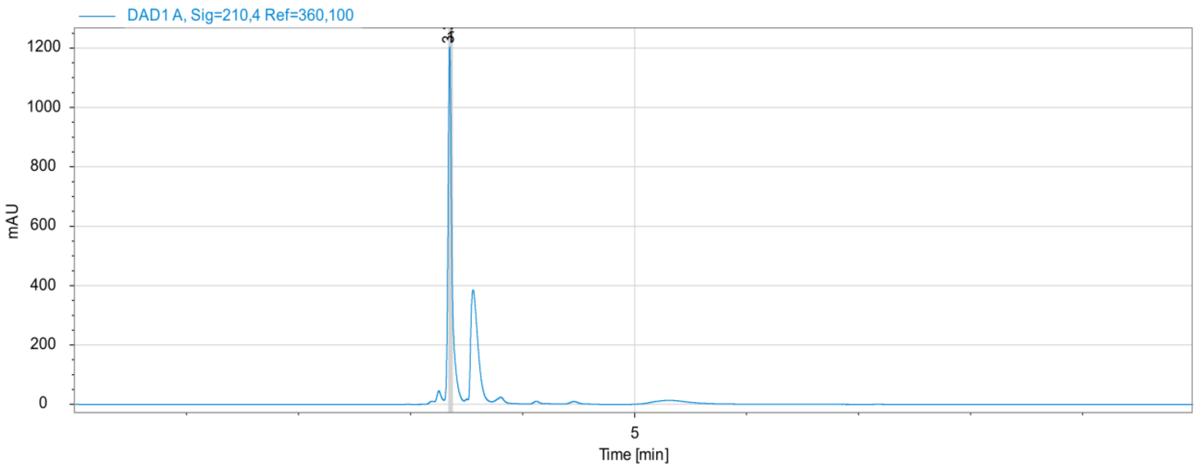

Peak	Retention time (min)
1	3.35

HPLC traces of *rac*-**1i**

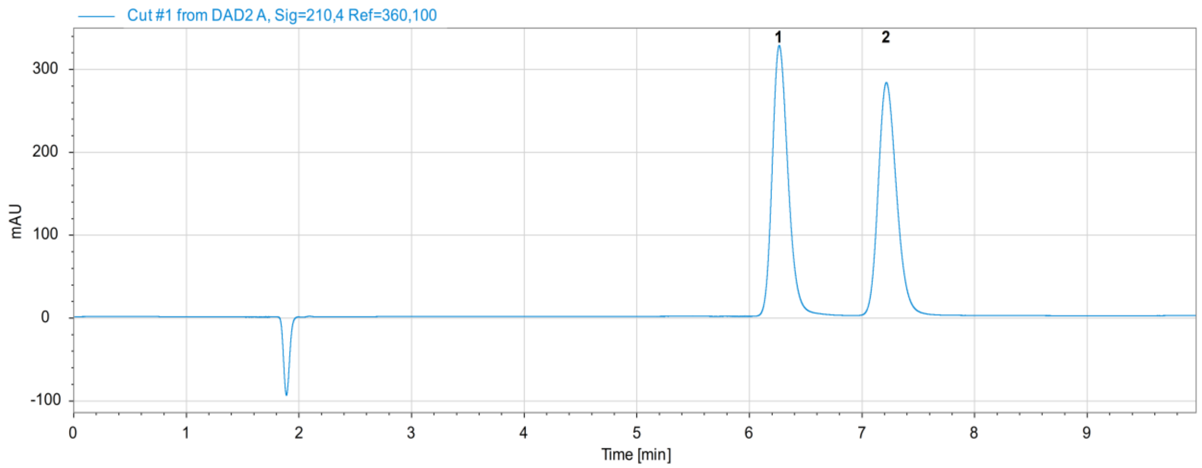

Peak	Retention time (min)	Area (%)
1	6.26	50.2
2	7.22	49.8
Total		100

HPLC traces of (*S*)-**1i**

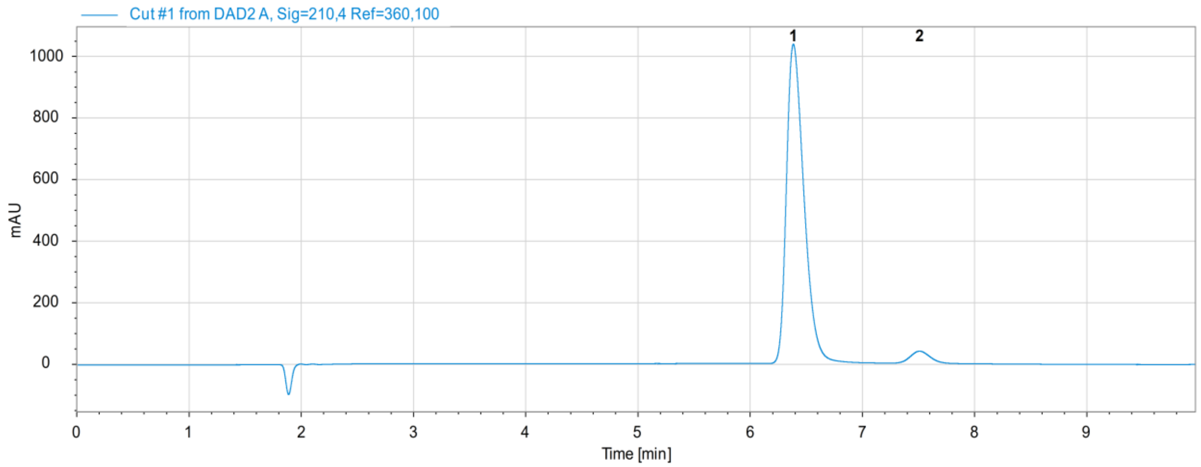

Peak	Retention time (min)	Area (%)
1	6.38	95.9
2	7.51	4.1
Total		100

HPLC traces of *rac*-**1j** and (*S*)-**1j**

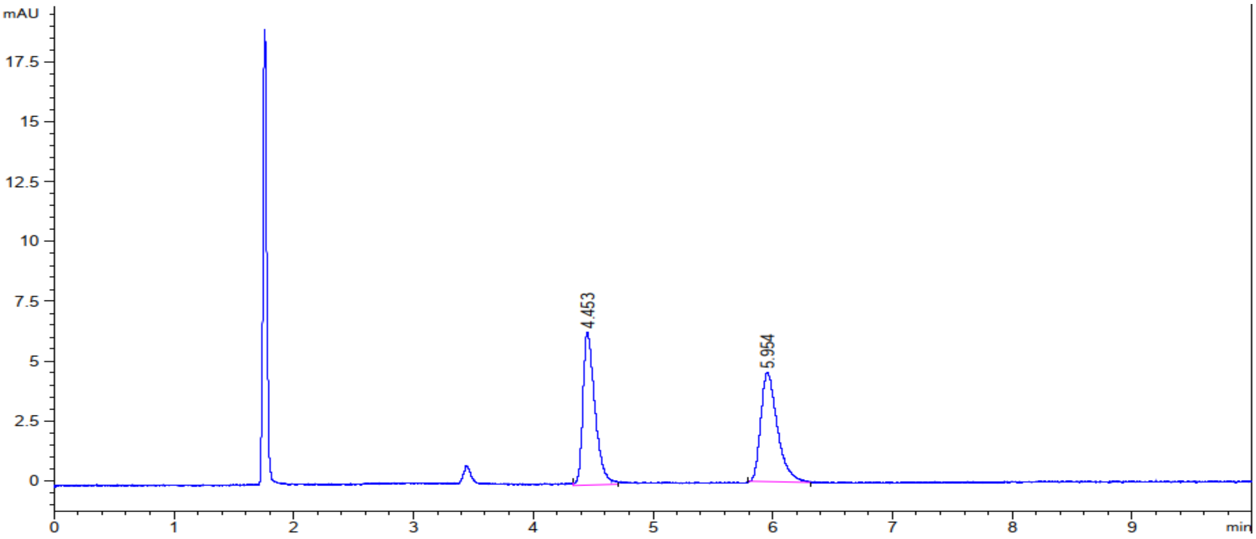

Peak	Retention time (min)	Area (%)
1	4.45	50
2	5.95	50
Total		100

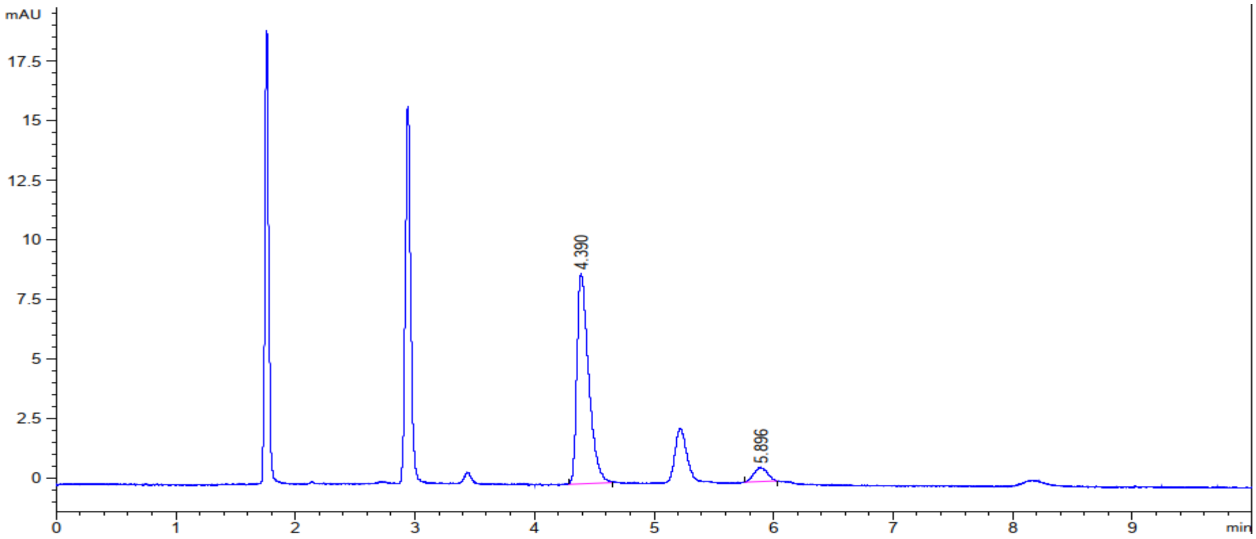

Peak	Retention time (min)	Area (%)
1	4.39	92.8
2	5.90	7.2
Total		100

HPLC traces of *rac*-**1k**

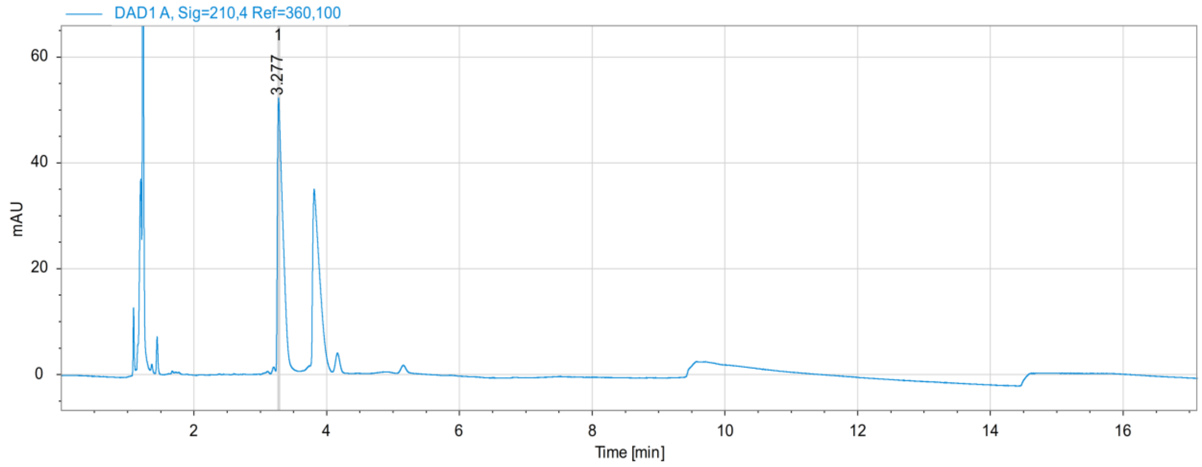

Peak	Retention time (min)
1	3.28

HPLC traces of (*S*)-**1k**

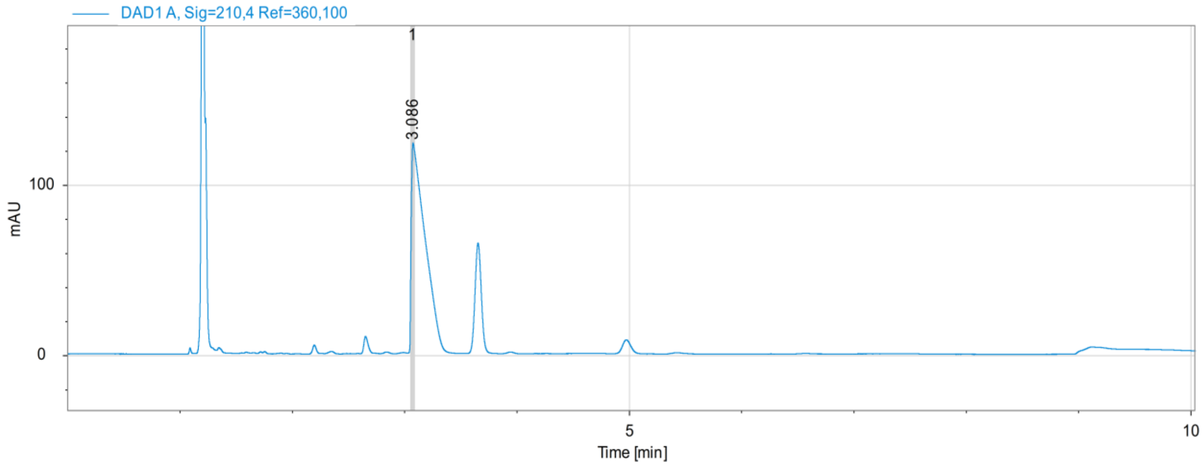

Peak	Retention time (min)
1	3.09

HPLC traces of *rac*-**1k**

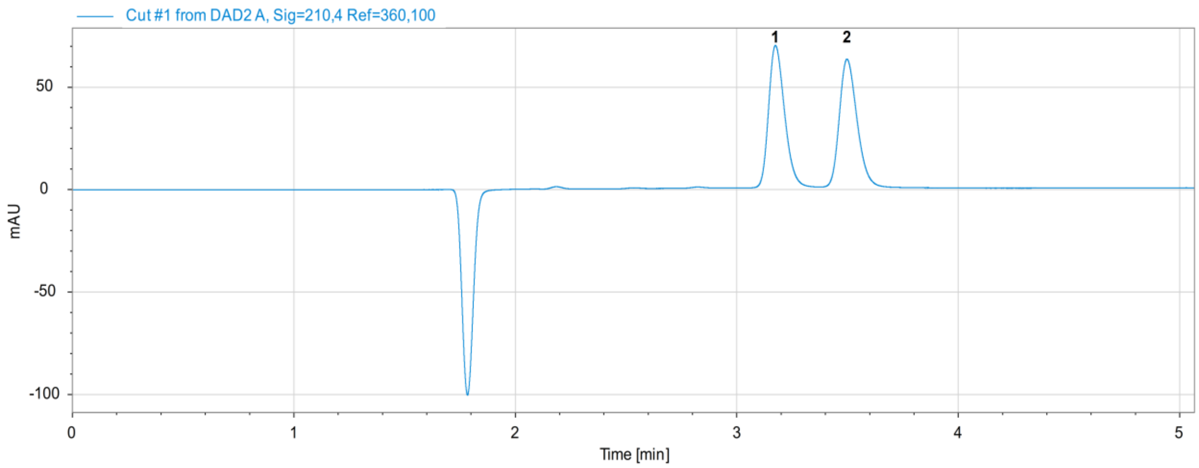

Peak	Retention time (min)	Area (%)
1	3.18	50.0
2	3.50	50.0
Total		100

HPLC traces of (*S*)-**1k**

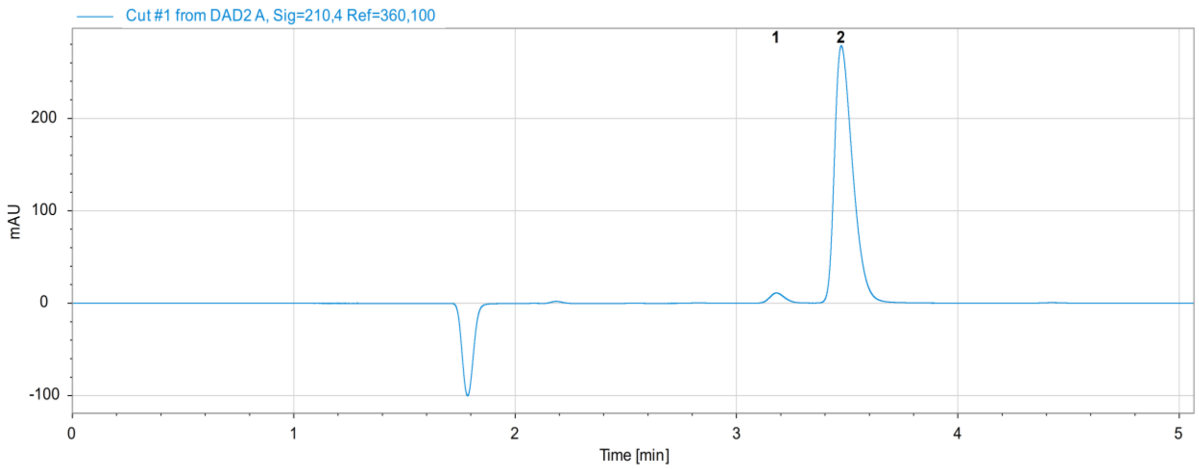

Peak	Retention time (min)	Area (%)
1	3.18	3.2
2	3.47	96.8
Total		100

HPLC traces of *rac*-**1l** and (*S*)-**1l**

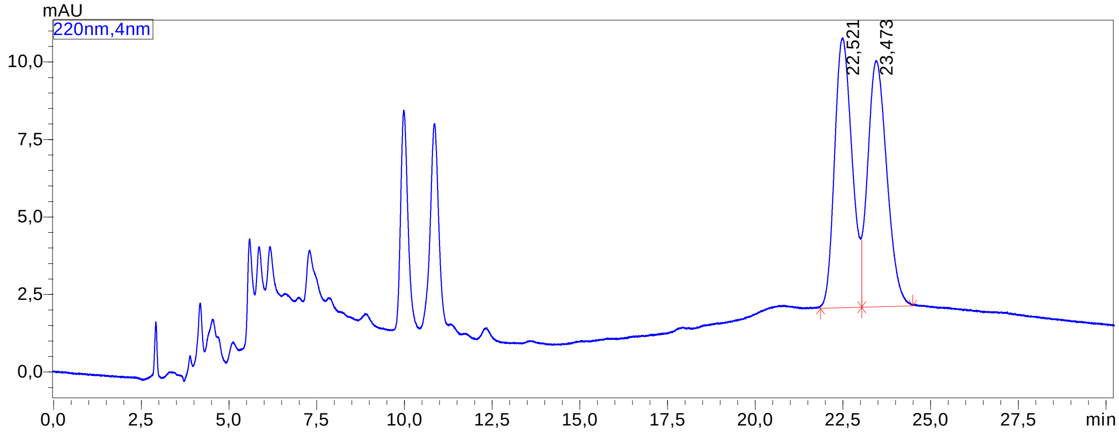

**Peak**	**Retention time (min)**	**Area (%)**
1	22.52	50
2	23.47	50
Total		100

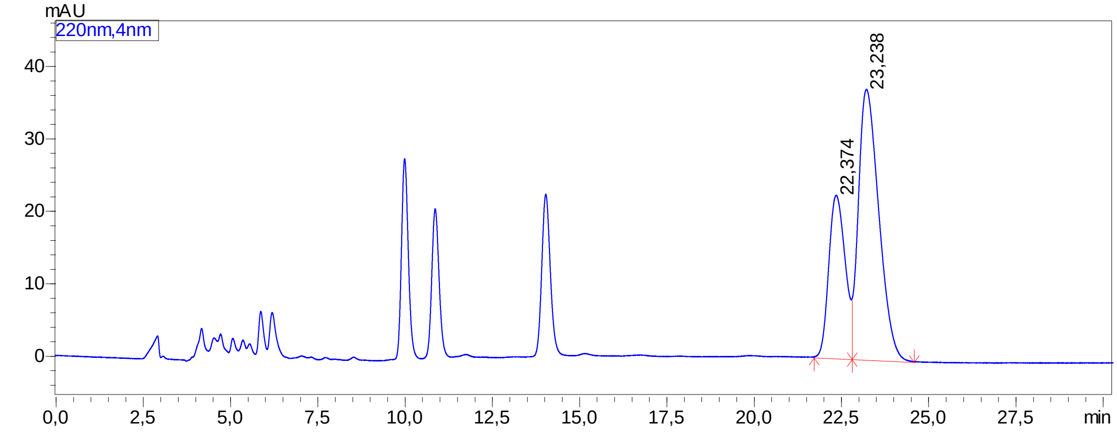

**Peak**	**Retention time (min)**	**Area (%)**
1	22.37	32.3
2	23.24	67.7
Total		100

HPLC traces of *rac*-**1m** and (*R*)-**1m**

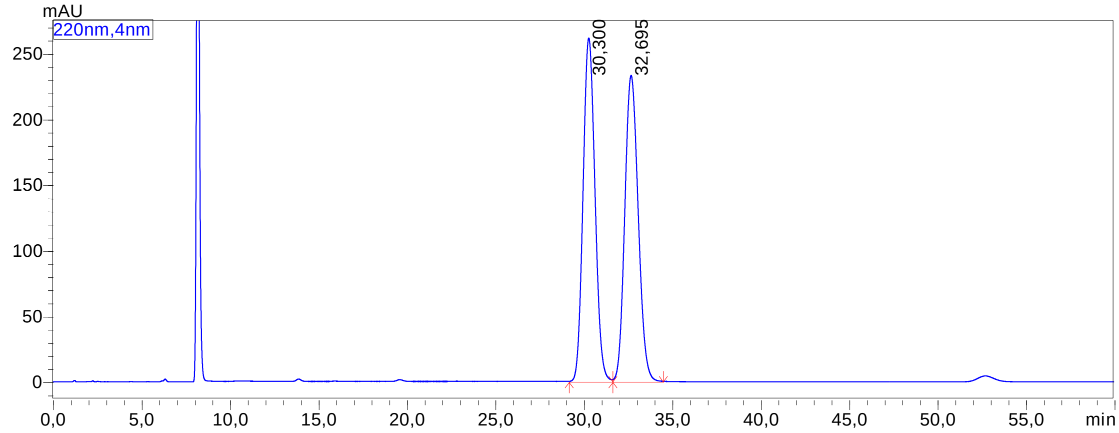

Peak	Retention time (min)	Area (%)
1	30.30	50
2	32.70	50
Total		100

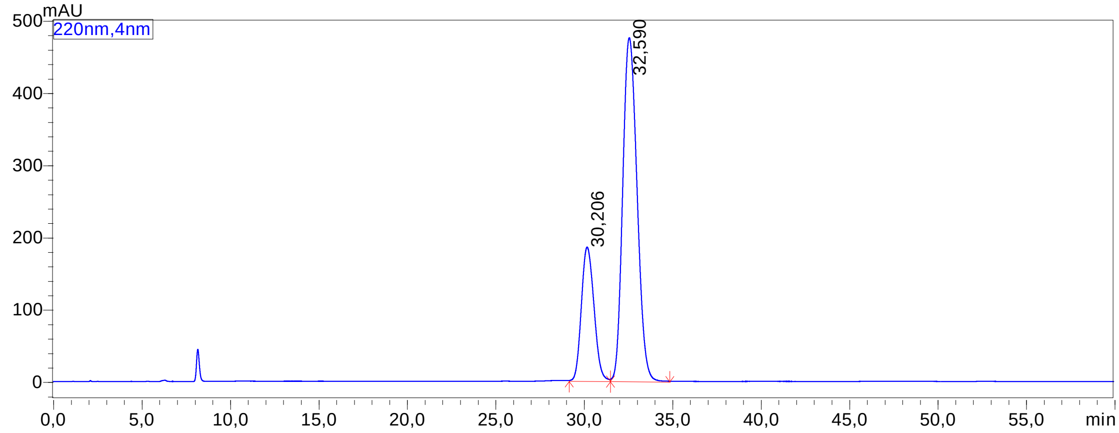

Peak	Retention time (min)	Area (%)
1	30.21	25
2	32.59	75
Total		100
